# Human Peripheral Blood Mononucleocyte Derived Myeloid Committed Progenitor Cells Mitigate H-ARS by Exosomal Paracrine Signal

**DOI:** 10.3390/ijms23105498

**Published:** 2022-05-14

**Authors:** Rishi Man Chugh, Payel Bhanja, Ximena Diaz Olea, Fang Tao, Kealan Schroeder, Ryan Zitter, Tanu Arora, Harsh Pathak, Bruce F. Kimler, Andrew K. Godwin, John M. Perry, Subhrajit Saha

**Affiliations:** 1 Departments of Radiation Oncology, University of Kansas Medical Center, Kansas City, MO 66160, USA; rchugh@kumc.edu (R.M.C.); pbhanja@kumc.edu (P.B.); xdiazolea@kumc.edu (X.D.O.); rzitter@kumc.edu (R.Z.); tarora2@kumc.edu (T.A.); bkimler@kumc.edu (B.F.K.); 2 Departments of Pediatrics, Children’s Mercy Kansas City, Kansas City, MO 64108, USA; ftao@cmh.edu (F.T.); kjschroeder@cmh.edu (K.S.); jmperry@cmh.edu (J.M.P.); 3 Department of Cancer Biology, University of Kansas Medical Center, Kansas City, MO 66160, USA; hpathak@kumc.edu (H.P.); agodwin@kumc.edu (A.K.G.); 4 Department of Pathology and Laboratory Medicine, University of Kansas Medical Center, Kansas City, MO 66160, USA; 5 Department of Pediatrics, University of Kansas Medical Center, Kansas City, MO 66160, USA; 6 Departments of Pediatrics, University of Missouri Kansas City School of Medicine, Kansas City, MO 64108, USA

**Keywords:** PBMC, bone marrow, myeloid, irradiation, stem/progenitor cells, mouse models

## Abstract

Radiation-induced loss of the hematopoietic stem cell progenitor population compromises bone marrow regeneration and development of mature blood cells. Failure to rescue bone marrow functions results in fatal consequences from hematopoietic injury, systemic infections, and sepsis. So far, bone marrow transplant is the only effective option, which partially minimizes radiation-induced hematopoietic toxicities. However, a bone marrow transplant will require HLA matching, which will not be feasible in large casualty settings such as a nuclear accident or an act of terrorism. In this study we demonstrated that human peripheral blood mononuclear cell-derived myeloid committed progenitor cells can mitigate radiation-induced bone marrow toxicity and improve survival in mice. These cells can rescue the recipient’s hematopoietic stem cells from radiation toxicity even when administered up to 24 h after radiation exposure and can be subjected to allogenic transplant without GVHD development. Transplanted cells deliver sEVs enriched with regenerative and immune-modulatory paracrine signals to mitigate radiation-induced hematopoietic toxicity. This provides a natural polypharmacy solution against a complex injury process. In summary, myeloid committed progenitor cells can be prepared from blood cells as an off-the-shelf alternative to invasive bone marrow harvesting and can be administered in an allogenic setting to mitigate hematopoietic acute radiation syndrome.

## 1. Introduction

Radiological terrorism and nuclear threat are ongoing concerns due to their potential to cause acute radiation syndrome (ARS) [1,2]. High doses of radiation can cause irreparable harm to the bone marrow, initiating leukopenia and increased risk of infection [3,4,5]. Granulocyte-colony-stimulating factor (G-CSF), which stimulates the bone marrow to produce granulocytes, is the only FDA-approved drug for protection of radiation-induced tissue damage, but showed limited efficacy in a non-human primate model [6]. Moreover, it is not effective in cases of complete myeloablation and may be associated with thromboembolic events. As such, there is no known strategy of post-exposure therapeutic intervention to rescue/salvage organs at risk in ARS within days after the radiation event has occurred.

ARS is very much dose-dependent, as evident in multiple organs including skin [7], the hematopoietic system [8], and the gastrointestinal tract [9]. Bone marrow is one of the most susceptible tissues for radiation toxicity [10,11]. Bone marrow suppression is evident even with a sub-lethal dose of radiation [11]. Due to a high rate of self-renewal, hematopoietic stem cells (HSC) are very sensitive to radiation, which leads to the failure of bone-marrow [12]. Even with a low dose of whole-body irradiation (1 Gy) to mice there was reduced engraftment capacity of bone marrow derived cells to about 50% compared with non-irradiated control bone marrow [13]. Bone marrow suppression by a sub-lethal irradiation can cause a patient to be immunosuppressed due to a reduced number of functional blood cells. A high dose of radiation will lead to irreversible bone marrow failure and eventually to death in the absence of intervention. Transplantation of bone marrow will partially restore hematopoiesis in lethally irradiated subjects, but it is not feasible to perform this in large casualty settings. Therefore, there is an unmet need to develop a countermeasure to mitigate hematopoietic acute radiation syndrome (h-ARS) that can be applicable in a mass casualty setting even when initiated 24 h after radiation exposure.

Previous studies have reported radiation countermeasures against ARS ranging from small molecules to growth factors and cytokines [14]. There are several bioengineered analogs of erythropoietin (Aransep, Epoetin, Epogen, Darbepoetin, and Procrit) that are commonly used for various hematologic indications. The primary indication of these agents is for the treatment of severe anemia via stimulation of erythropoiesis following intense chemotherapy or radiotherapy [15,16]. However, these recombinant agents are still not approved as a medical countermeasure for use in radiation casualties. Furthermore, leridistim (a chimeric dual G-CSF and IL-3 receptor agonist), which shows efficacy in ameliorating severe, radiation-induced neutropenia within large, experimental animals, despite promising initial results, is still not being developed as a countermeasure [17]. Moreover, considering the complexity of h-ARS, a polypharmacy approach may be a more suitable option [18]. However, proper design and optimization will be a major challenge. Cell-based therapy can be considered as a natural polypharmacy solution as it delivers multiple paracrine signals including cytokines and growth factors [19] critical for radio-mitigation.

Cell-based therapy has now been extended to stem cell therapy and/or stromal cell-based therapy [20]. However, these approaches are not feasible in an unplanned, large-scale allogenic setting. Stem/progenitor cell therapy such as mesenchymal stem cells (MSCs) has been used to mitigate radiation injury [21,22]. Although preclinical evidence suggests that MSCs developed from bone marrow, adipose-derived cells, or induced pluripotent stem cells (iPSCs) are promising candidates for regenerative cell therapy, clinical evidence indicates a clear discrepancy between expected and actual outcomes of MSCs taken from bench to bedside [23,24]. So far there is no cell-based therapy available to mitigate ARS when applied beyond 24 h post-irradiation in an allogeneic setting. Autologous cell transplantation can be an alternative option but is only applicable to first responders and/or military personnel if their own tissue is bio banked in advance.

In the present study, we demonstrate that human peripheral blood mononuclear cells derived of a lineage-negative population enriched in myeloid-committed progenitor cells (hMCPs) can mitigate h-ARS in mice even in an allogenic recipient. hMCPs have an advantage over bone-marrow-derived MSCs because they can be cultured from blood obtained via a minimally invasive collection method compared with the painful collection and tedious isolation and processing of cells from bone marrow. In summary, our studies demonstrate that hMCP transplantation at 24 h or later can mitigate h-ARS in mice. Extracellular vesicles derived from hMCPs mediate the release of paracrine signals, which play a significant role in the amelioration of radiation-induced reduction of HSCs, maintaining bone marrow function and preventing radiation-induced lethality via h-ARS.

## 2. Results

### 2.1. Transplantation of hMCP Cells Mitigates h-ARS and Improves Survival

**Figure 1:**
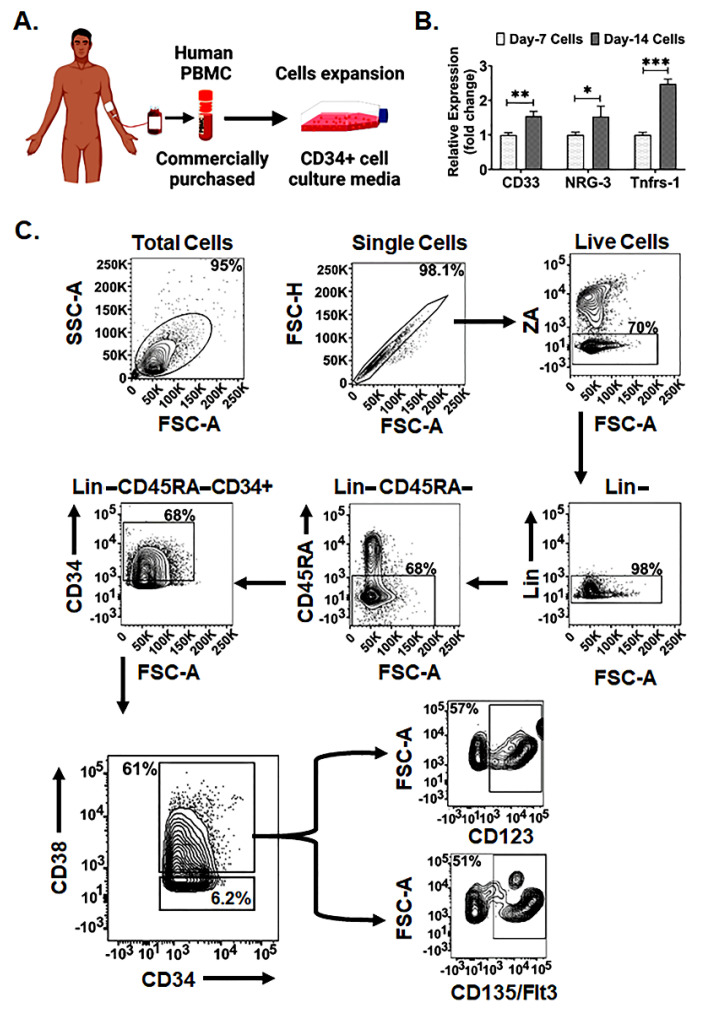
Characterization of PBMC-derived human myeloid-committed progenitor cells (hMCPs). (**A**) Schematic representation of hMCP cells expansion from a frozen vial of PBMC. (**B**) Fold change relative expression of myeloid-committed progenitor cells marker expression between day 7 vs day 14 collected cells. Data are presented as the mean ± SD. (Significance level, *: *p* < 0.05, **: *p* < 0.005, ***: *p* < 0.0005). (**C**) Flow cytometric characterization of ex vivo expanded cells depicts 98% Lin−cells population in the single live cells analyzed, which give rise to 68% of Lin−CD45RA−cells being CD34+ and 61% of Lin−CD45RA−CD34+ cells being CD38+, while the percentage of Cd34+Cd38− was 6.2%. The phenotypic characterization of Lin−CD45RA−CD34+CD38+ cells expressed myeloid cell markers: 57% being CD123+ and 51% being CD135/Flt3+.

Human PBMC-derived Lin-CD34+ cells were cultured in myeloid progenitor expansion medium to enrich Lin-CD45RA-CD34+CD38+ cells (Figure 1A–C). The expanded cells collected at day 14 demonstrated significantly higher expression of myeloid-committed progenitor cell markers; 1.5 fold for *CD33* (*p* < 0.005), 1.5 fold for *NRG-3* (*p* < 0.05), and 2.5 fold for *Tnfrs-11a* (*p* < 0.0005) then the cells collected at day 7 (Figure 1B). Therefore, in all the experiments of this study we used cells collected after 14 days of expansion.

**Figure 2:**
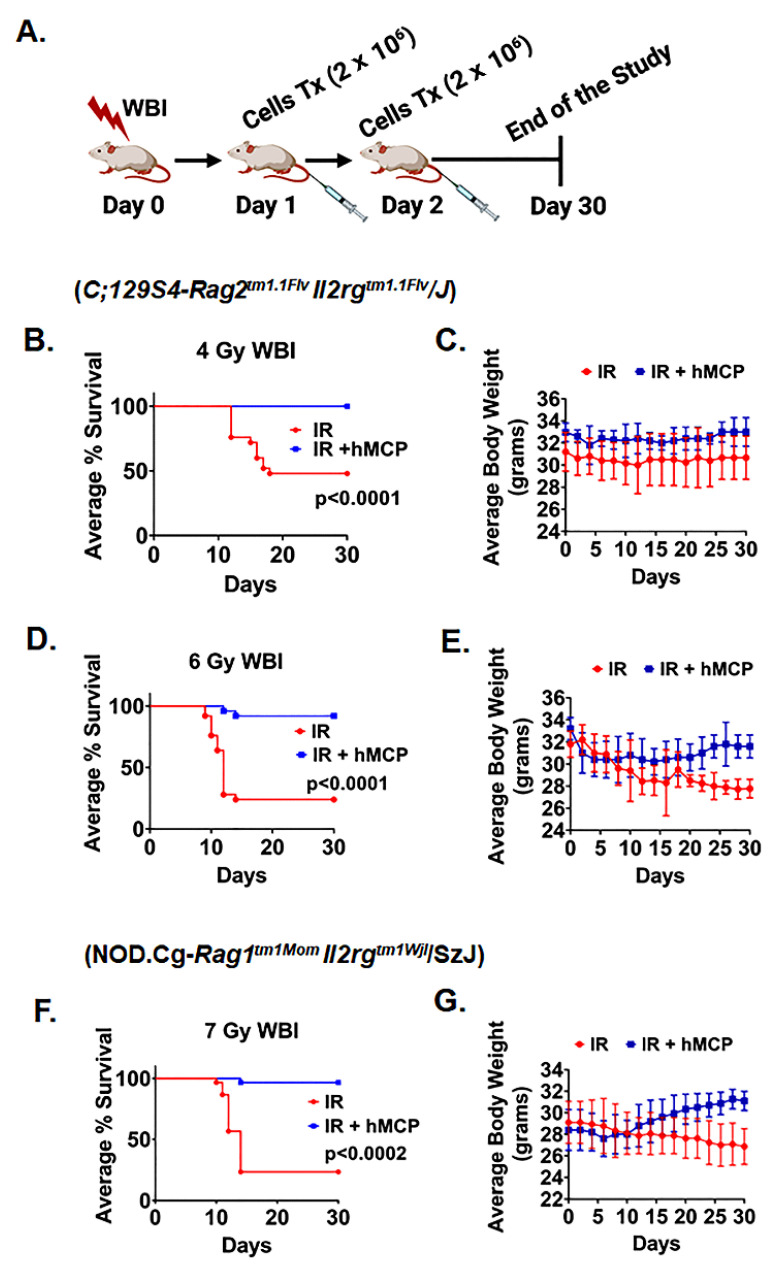
hMCP cell transplantation improves the survival of mice following whole-body irradiation. (**A**) Schematic representation of the survival experimental plan (radiation doses and timeline for cell transplantation). Mice receiving hMCP cells 24 and 48 h after irradiation and Kaplan–Meier survival (Mantel–Cox test) analysis of (**B**) *C;129S4-Rag2^tm1.1Flv^ Il2rg^tm1.1Flv^/J* (*Rag2-γC*) mice showed 100% survival with 4 Gy WBI (*p* < 0.0001) in the IR + hMCP group and (**C**) no change in body weight of the IR and IR + hMCP group (*n* = 25 mice per group) (**D**) Rag2-γC mice with 6 Gy WBI also showed 92% survival in the IR + hMCP group compared with the IR group (*p* < 0.0001) (**E**) shows improvement in body weight in IR + hMCP group compared with the IR group (n = 25 mice per group). (**F**) *NOD.Cg-Rag1^tm1Mom^ Il2rg^tm1Wjl^/SzJ* (*NRG*) mice showed 96.6% survival in the IR + hMCP group compared with the IR group (*p* < 0.0002) and (**G**) shows recovery in body weight in the IR + hMCP-transplanted group compared with the IR group (*n* = 30 mice per group).

To test the potential radio-mitigating effect of hMCPs, immunocompetent *C;129S4-Rag2^tm1.1Flv^ Il2rg^tm1.1Flv^/J* (*Rag2-γc*-) mice (*n* = 25 per group) were exposed to whole-body irradiation (Figure 2A). First, we exposed *Rag2-γc*- mice to a dose range of 4–6 Gy where 6 Gy represented LD70/30 dose level. Mice receiving two doses of intravenous cells transfusion (2 × 10^6^ cells/mice/transfusion) at 24 h and 48 h post irradiation demonstrated significant improvement in survival (4 Gy *p* < 0.0001; 6 Gy; *p* < 0.0001) compared with irradiated untreated controls (Figure 2B,D). To validate the radio-mitigating role of hMCPs in a second strain, immunocompetent *NOD.Cg-Rag1^tm1Mom^ Il2rg^tm1Wjl^/SzJ* (*NRG*) mice (*n* = 30 per group) were exposed to 7 Gy (LD70/30) and then treated with same dose and schedule of hMCP cell transfusion. Please note that the radiation dose level was increased to achieve LD70/30 due to differences in radiosensitivity in *NRG* mice compared with *Rag2-γc* mice. *NRG* mice receiving cell transfusion demonstrated a significant improvement in survival compared with irradiated controls (7 Gy; *p* < 0.0002) (Figure 2F). Mice with hMCP cell transplantation also demonstrated better restitution of body weight compared with irradiated untreated controls (Figure 2C,E,G). In a separate study, *NRG* mice receiving a single dose of hMCP transfusion at 24 h after irradiation also showed significant improvement in survival (*p* < 0.024) (*n* = 12 mice per group) (Supplementary Figure S1) compared with irradiated controls. As whole-body irradiation with an LD70/30 dose primarily involves hematopoietic syndrome, our results clearly suggested that PBMC-derived hMCPs can be considered as potential mitigators against h-ARS.

Optimization of cryopreservation and minimization of cell preparation time are very important to consider any cell-based therapy for point-of-care treatment against acute radiation syndrome. In the current study, hMCPs were cryopreserved in CS5 cryopreservation media at liquid nitrogen temperature. Before treatment, cryopreserved hMCPs were quickly thawed to 37 °C in a water bath followed by incubation with culture media at 37 °C for 10 h. Mice receiving two doses of this cell preparation (2 × 10^6^ cells/mice/transfusion) at 24 h and 48 h post irradiation demonstrated a significant improvement in survival (*p* < 0.005) (Supplementary Figure S2).

### 2.2. hMCP Rescues HSCs and Induces Regenerative Potential against Radiation Injury

**Figure 3:**
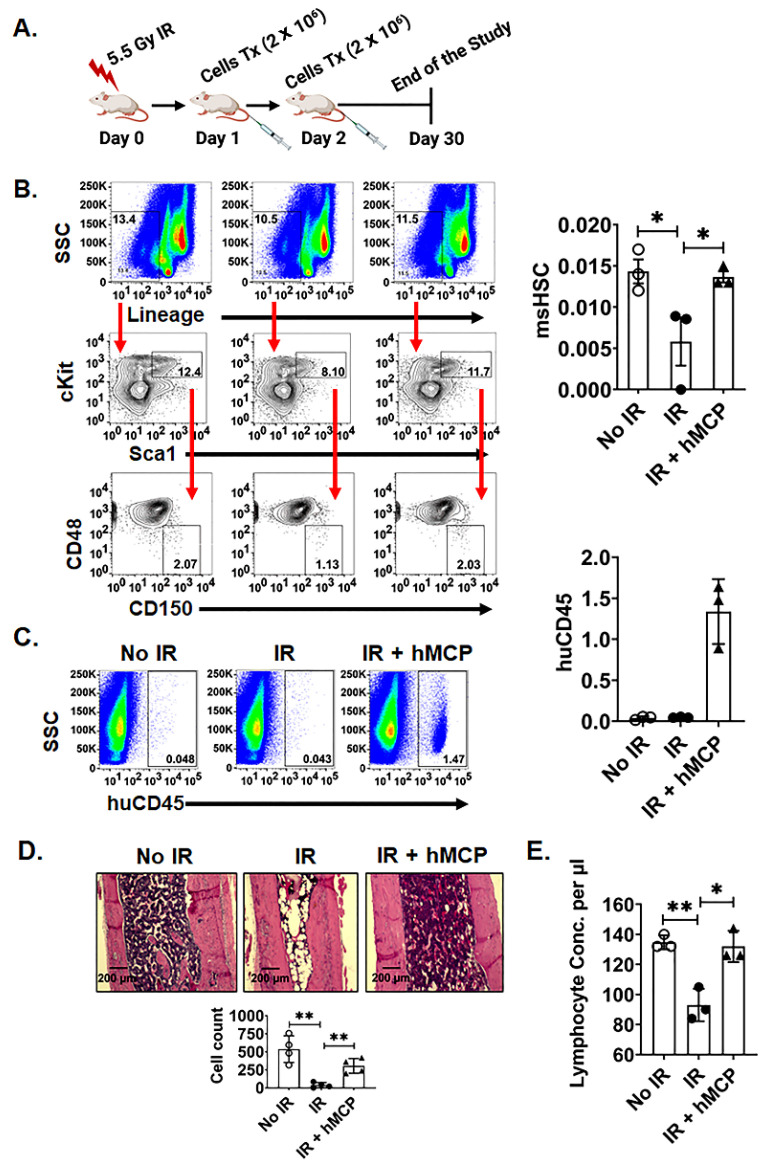
hMCP cell transplantation rescues HSCs and induces regenerative potential against radiation injury. (**A**) Schematic representation of the experimental plan (radiation dose and timeline for cells transplantation). (**B**) Recovery of mouse bone-marrow HSCs after hMCP transplantation in comparison with the IR group (*p* < 0.05) (**C**) Presence of transplanted human cells in mouse bone-marrow after four weeks of transplantation, identified using CD45 marker expression (**D**) Histopathological analysis with cell counts of bone-marrow shows the recovery of bone-marrow after hMCP transplantation compared with the IR group (*p* < 0.005) (**E**) Blood analysis revealed that the lymphocyte concentration was significantly improved in the IR + hMCP group compared with IR alone (*p* < 0.05). No IR group represented by hollow circle, IR group represented by filled circle, IR + hMCP group represented by triangle. Data are presented as the mean ± SD. (Significance level, *: *p* < 0.05, **: *p* < 0.005).

Radiation-induced loss of HSCs significantly impairs bone marrow regeneration [25,26]. As hMCPs rescued bone marrow and improved survival post-irradiation, we further examined the effect of hMCP transfusion on HSC survival and regenerative function in irradiated mice [12,27]. Radiation reduced the number of mouse HSCs; however, transplantation of hMCPs resulted in recovery of mouse HSCs (*p* < 0.034) (Figure 3A,B). We also detected the presence of human cells in xenograft-recipient bone marrow even after 28 days post irradiation (Figure 3C), suggesting their involvement in mouse bone marrow regeneration. To further validate bone-marrow recovery, we analyzed bone histology in these mice and found that radiation caused a significant loss of cellularity in bone marrow, which recovered after transplantation of hMCP cells (*p* < 0.005) (Figure 3D). Analysis of blood shows that lymphocyte titers were significantly less in the radiation group, which also recovered after hMCP cell transplantation (*p* < 0.01) (Figure 3E). These data demonstrate that hMCPs specifically enhance the recovery of HSCs following irradiation.

### 2.3. hMCPs Are Suitable for Allogenic Transplant

**Figure 4:**
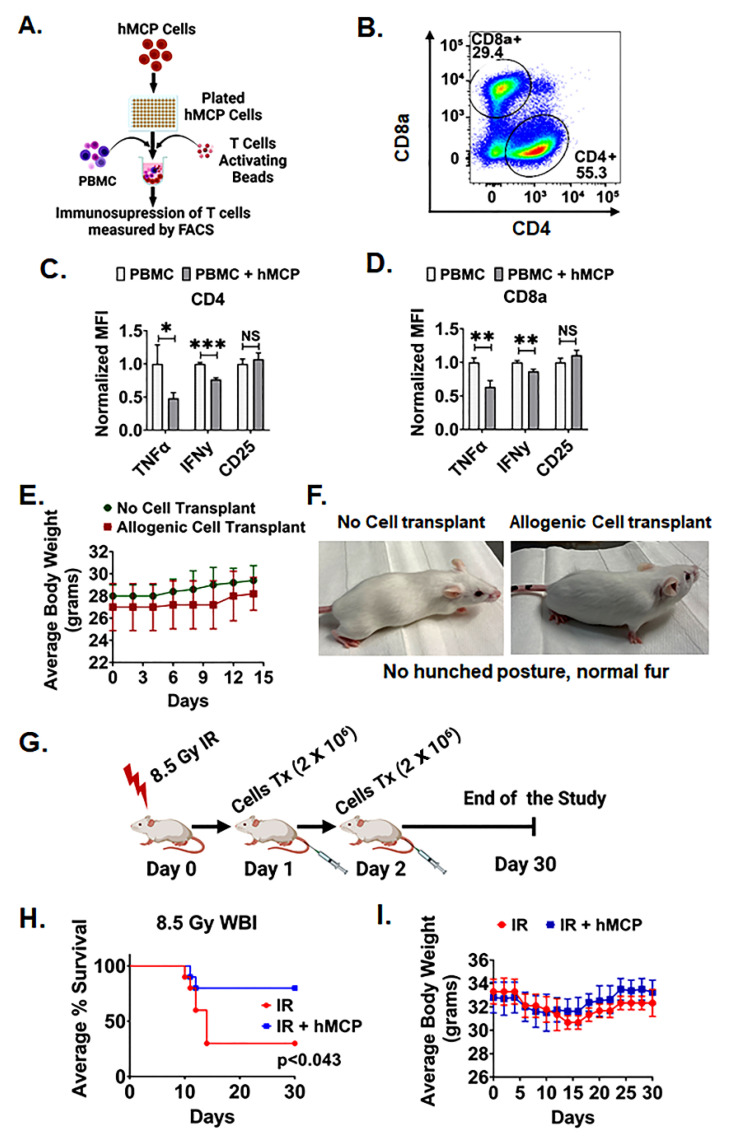
hMCP cells are suitable for allogenic cell transplantation. (**A**) Schematic representation of experimental plan to elucidate immunosuppressive role of hMCP cells. (**B**) Flow plot representing two different CD8a and CD4 cell populations. (**C**,**D**) hMCP cells co-cultured with activated T cells suppress the expression of *TNFα* and *IFNγ* in both CD4+ (*TNFα*: *p* < 0.05 and *IFNγ*: *p* < 0.005) and CD8a (*TNFα*: *p* < 0.005, and *IFNγ*: *p* < 0.005). T cells in comparison to control group. No significant changes in the expression of CD25 in both CD8+ and CD4+ T cell population. (**E**) Allogenic cell-transplanted mice group shows no change in average body weight, (**F**) no hunched posture, and normal fur structure compared with the no cell transplant group mice (n = 10 mice per group). (**G**) Schematic representation of the timeline for the hMCP cell transplantation in FVB mice after 8.5 Gy irradiation. (**H**) Kaplan–Meier survival (Mantel–Cox) analysis of allogenic hMCP transplanted FVB mice (cross species cells transplantation) after irradiation shows 80% survival of the mice after allogenic cell transplantation. (**I**) The change in average body weight is comparable to the irradiated control mice (n = 10 mice per group). Data are presented as the mean ± SD. (Significance level, *: *p* < 0.05, **: *p* < 0.005, ***: *p* < 0.0005, NS: Not significant).

To examine the immune-modulatory function of hMCPs, we developed a co-culture system containing human PBMC, T cells activation beads with/without hMCP cells (Figure 4A). Presence of hMCPs significantly suppressed the expression of *TNFα* and *IFNγ* in CD4+ and CD8+ T cell populations. Presence of hMCP reduced the expression of *TNFα* (PBMC: 1 ± 0.03 fold vs PBMC + hMCP: 0.48 ± 0.09 fold, *p* < 0.0005) and *IFNγ* was (PBMC: 1 ± 0.02 fold vs PBMC + hMCP: 0.76 ± 0.02 fold, *p* < 0.0005) in CD4 T cells (Figure 4B,C). A similar observation was made in CD8a+ T cells where hMCPs suppressed the expression of *TNFα* (PBMC: 1 ± 0.26 fold vs PBMC + hMCP: 0.64 ± 0.10 fold, *p* < 0.005) and *IFNγ* (PBMC: 1 ± 0.03 fold vs PBMC + hMCP: 0.87 ± 0.03 fold, *p* < 0.005) in CD8a T cells (Figure 4B,D). We did not observe a significant change in the expression of the T cell marker CD25 in either the CD4 or CD8a T cell populations. These results suggest that the hMCP cells have an immunomodulatory function that makes them suitable studies of for allogenic cell transplantation.

Next, we examined whether hMCP can be transplanted in an allogenic setting without triggering a GVHD-like symptom. hMCPs were transplanted into non-immunocompetent FVB mice. FVB mice receiving the hMCP transplant did not show any GVHD-like symptoms such as weight loss, hunched posture, or hair loss (Figure 4E,F). The survival study of irradiated mice receiving hMCPs demonstrated 80% survival as compared with the 30% survival in the control irradiated group (*p* < 0.043) with no sign of GVHD until end of the study (Figure 4G), while no significant changes in body weight of the mice in either group was observed (Figure 4H). 

**Figure 5:**
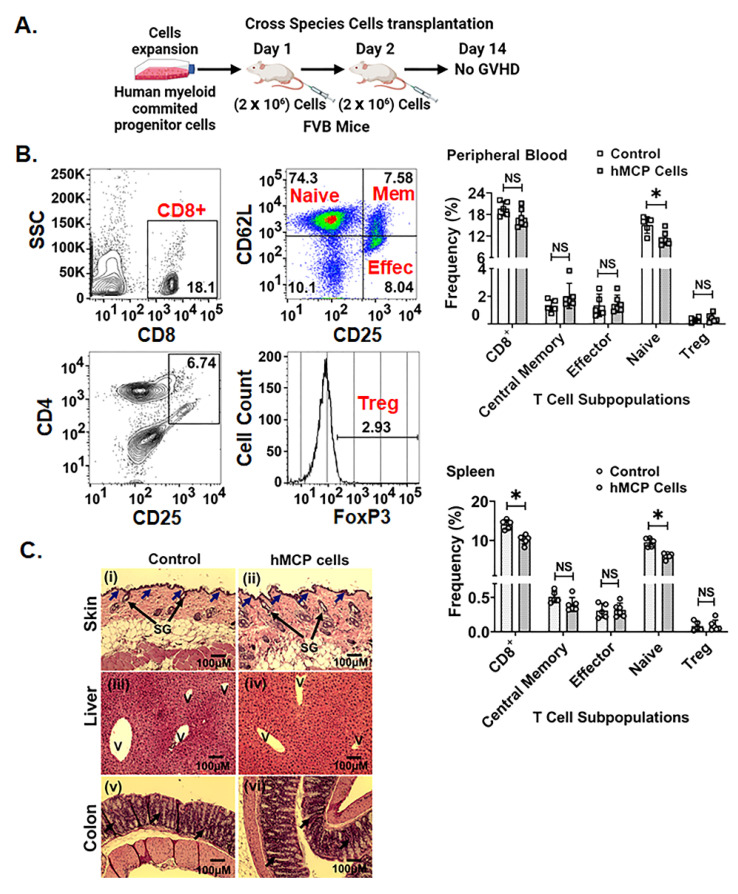
Cross species transplantation of hMCP does not produce graft-versus-host disease: (**A**) schematic representation of timeline for the hMCP cell transplantation in mice, (**B**) peripheral blood and spleen analysis of the mice 14 days post cells transplantation shows no immune cell activation in both the tissues. Besides that, hMCP suppresses the CD8+ (*p* < 0.0004) subpopulation in the spleen, while Naïve T cells in the spleen (0.0001) and the peripheral blood sample (*p* < 0.02) (**C**) in both the skin groups (i,ii) showed no damage at the dermal and epidermal junctions (shown by blue arrow) with normal sebaceous glands (large black arrow); (iii,iv) the liver showed no cell infiltration in the liver vein (V) and (v,vi) the colon showed no damage or necrotic changes in the crypt (black arrow). Data are presented as the mean ± SD. (Significance level, *: *p* < 0.05, NS: Not significant, SG: sebaceous gland, V: vein).

The peripheral blood and splenic cell analysis of the *FVB* mice receiving hMCPs did not demonstrate any immune activation (Figure 5A). In fact, hMCP cells transplantation significantly suppressed CD8+ (*p* < 0.05) and Naïve T cells (*p* < 0.05) subpopulation in the spleen (Figure 5B), and naïve T cell subpopulation in peripheral blood samples (*p* < 0.02) (Figure 5B). No significant change was observed in other analyzed T cell subpopulations (Central memory T cells, Tregs and Effector T cells) in spleen in response to hMCP treatment (Figure 5B). This further confirms that the immunosuppressive function of hMCP cells makes them suitable for allogenic cell transplantation.

GVHDs induces significant pathological changes in organs like skin, liver, and colon [28,29]. In response to hMCP transplant, absence of any GVHD-related tissue damage was confirmed by histopathological analysis of skin, liver, and colon. hMCP transplant did not induce any vacuolization in the epidermal region in the skin (Figure 5C(i,ii)). Analysis of liver sections in hMCP-treated mice did not show any damage or change in immune cell infiltration compared to irradiated mice receiving no cell transplant (Figure 5C(iii,iv)). Mice with hMCP transplant did not show any cryptal abscess and necrosis in colon, a typical pathology commonly observed in GVHD (Figure 5C(v,vi)). The absence of any evidence for GVHD indicated that hMCPs are suitable for allogenic cell transplantation.

### 2.4. hMCP-Derived Paracrine Signals Are Enriched with Exosomal Proteins

Our previous reports as well as reports from other groups suggested that following intravenous transplantation, cells are initially docked in the lung [30,31,32]. Therefore, the radio-mitigating function of these cells to the distant injury site such as bone marrow was primarily dependent on cell-derived paracrine signals. As we observe that transfused hMCPs primarily lodged in lung and bone marrow (Supplementary Figure S3), it is very possible that hMCP-derived paracrine signals play an important role in rescue of recipient mHSCs. We performed a proteomics analysis of hMCP-conditioned medium to determine paracrine signals. Out of a total of 1447 identified proteins, a significant presence of exosomal proteins (315) (Supplementary Figure S4 and Supplementary Table S2a,b) was observed. The majority of these proteins are involved in the regulation of biological functions such as cell metabolic process, regulation of biological process, cell organization, cell growth, and transport, which are important for regenerative processes, further suggesting possible the involvement of exosomal cargo for the radiomitigating role of hMCPs.

### 2.5. hMCP-Derived sEVs Mitigates h-ARS

**Figure 6:**
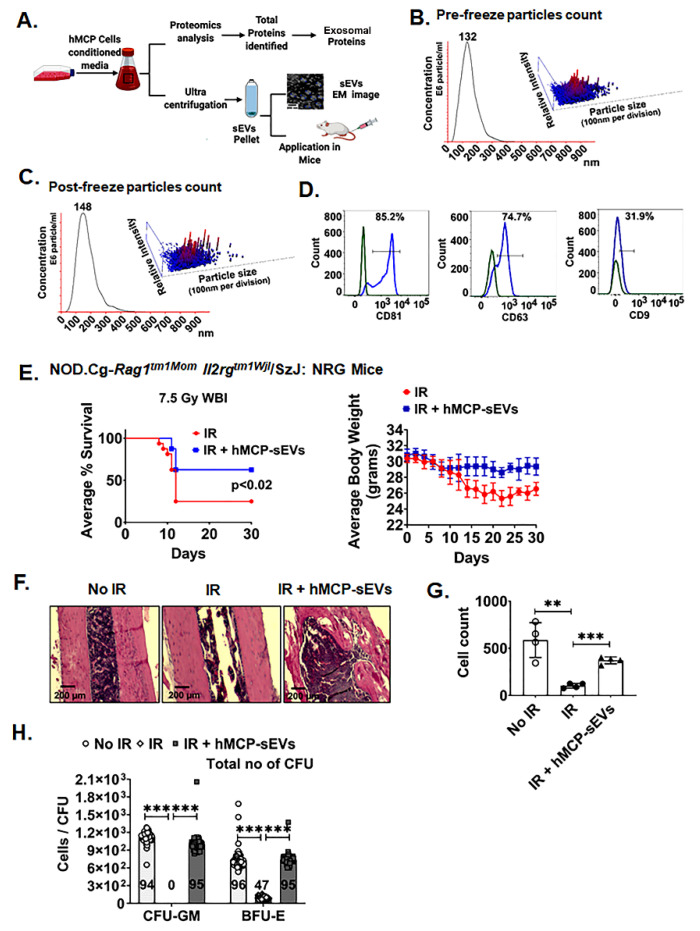
Transplantation of hMCP-derived sEVs promotes regenerative responses. (**A**) Schematic representation of proteomics analysis and sEVs isolation from hMCP-conditioned media and application in mice. (**B**) Pre-freeze and (**C**) Post-freeze sEVs particle count shows that particle counts are more stable even after freeze and thaw cycle of sEVs. (**D**) sEVs characterization by flowcytometric analysis shows presence of exosomal marker CD81 (85.2%), CD63 (74.7%), and CD9 (31.9%) respectively (**E**) Kaplan–Meier survival (Mantel–Cox) analysis of sEVs-transplanted *NOD.Cg-Rag1^tm1Mom^ Il2rg^tm1Wjl^/SzJ* (*NRG*) mice shows 62.5% mice survival in the sEVs-treated group compared with the control non-treated group (*p* < 0.02), The body weight in the sEVs group fell until day 12 and remained uniform until the end of the study (n = 16 mice per group). (**F**) Histopathological analysis of bone-marrow of the mice shows recovery of bone-marrow after IR + hMCP-exosome transplantation compared with the IR group (*p* < 0.0005). (**G**) Cell counts of bone-marrow shows recovery of bone-marrow after hMCP-sEVs transplantation compared with the IR group (*p* < 0.0005) (n = 10 mice per group) (**H**) hMCP-sEVs promoted regenerative response in irradiated HSCs. The sEVs-treated irradiated CD34+ cells give rise to a significantly higher number of CFU-GM (*p* < 0.0005) and BFU-E (*p* < 0.0005) colonies compared with the control irradiated group. Data are presented as the mean ± SD. (Significance level, **: *p* < 0.005, *** *p* < 0.0005).

EVs are one of the major carriers of cell-derived paracrine signals including nucleotides and proteins [33,34]. Due to enrichment in cell-derived cargos and efficient transport to target tissues due to their smaller size, EV-based therapies are now being prioritized as an alternative to cell transplantation [35]. In the present study, our proteomics data demonstrated that hMCP-conditioned media are enriched with exosomal protein (Figure 6A). To validate the involvement of EVs as a major carrier of hMCP-derived paracrine signals, we examined the radio-mitigating role of EVs in h-ARS. Considering the different types of extracellular vesicles with variable particle size, we validated the hMCP-derived EVs populations based on surface marker and particle size. We demonstrated that hMCP-derived extracellular vesicle particle sizes are primarily representing small EVs (sEVs) with modal range particle sizes of 132–148 nm [36,37]. sEVs counts were maintained even after a freeze–thaw cycle for over 72 h (Figure 6B,C). For subsequent experiment, cryopreserved exosomes were thawed prior to use. The flowcytometric analysis of purified sEVs demonstrated the presence of common exosomal surface markers expression of CD81 (85.2%), CD63 (74.7%), and CD9 (31.9%) (Figure 6D). Mice exposed to Whole Body Irradiation with 7.5 Gy were treated with purified sEVs at 24 h and 48 h post-irradiation (200 μg of sEVs per mouse at each time) by tail vein injection; 62.5% of mice receiving sEVs treatment survived beyond 30 days post-irradiation as compared with the 25% survival in the control irradiated group (*p* < 0.02). The body weight of the mice also showed less weight loss in the sEVs-treatment group compared with the untreated, irradiated mice (Figure 6E). Histological analysis of bone-marrow demonstrated damage of the cellular content in the irradiated group, which recovers in mice receiving hMCP sEVs treatment (Figure 6F). The total cell counts also showed recovery in the bone-marrow cell number after sEVs treatment (*p* < 0.0005) (Figure 6G). These data confirm that the hMCP-derived sEVs carry the important regenerative factor(s) which mitigate h-ARS.

### 2.6. hMCP sEVs Promote Regenerative Response of HSCs

To determine the regenerative effect of hMCP-derived sEVs in HSCs, a CFU assay was performed using CD34+ HSCs. CD34+ cells were exposed to 6 Gy of radiation and incubated with/without hMCP-derived sEVs. Cellular colonies representing colony forming unit–granulocyte, macrophage (CFU-GM) and burst forming unit–erythroid (BFU-E) were quantified at 14 days post-treatment. Presence of all these colonies in higher numbers was noted in irradiated cohorts receiving sEVs compared with irradiated controls (CFU-GM: IR + sEVs vs. IR, 95 vs. 0.0) (*p* < 0.0005) (BFU-E: IR + sEVs vs. IR, 95 vs. 47) (*p* < 0.0005) (Figure 6H). These results suggest that hMCP-derived sEVs carry important cargo to maintain the stemness of the irradiated HSCs. Therefore, hMCP-derived sEVs can also be an alternate therapeutic approach to mitigate radiation-induced injury.

### 2.7. hMCP-Derived sEVs miRNAs-Associated Regenerative and Immune-Modulatory Process

The sEVs miRNAs have properties to alter the biological pathways involved in cellular homeostasis, repair and regeneration, immunomodulation, etc. [38]. It is reported that miRNAs present in the sEVs can influence target cell function [39,40]. hMCP sEVs analysis identified miRNAs associated with HSC proliferation, regeneration, homeostasis, and differentiation (Table 1 and Supplementary Figures S5–S11). Moreover, these sEVs also carry immunomodulatory miRNAs as well. The GO analysis also identified different molecular functions such as protein binding, RNA binding, RNA polymerase binding, enzyme regulator activity, etc. being modulated by exosomal miRNA. Further, the KEGG analysis was used to predict the target signaling pathway for HSCs proliferation, regeneration, and differentiation being regulated by hMCP sEVs miRNA. Various signaling pathways related to bone marrow regeneration were identified such as the *TGF-β* signaling pathway [41], PI3K-Akt signaling pathway [42], chemokine signaling pathway [43], Hedgehog signaling pathway [44], Wnt signaling pathway [45], FoxO signaling pathway [46], etc. (Supplementary Figure S12–S15). Although further mechanistic and validation study is needed, our current observations clearly shed light on the hMCP paracrine activity responsible for the tissue-specific regenerative properties of hMCPs.

## 3. Discussion

The present study addressed a gap in cell-based therapy to mitigate H-ARS. Efforts have been made to use cell replacement therapy to ameliorate radiation-induced bone marrow syndrome and multiple sources such as bone marrow or adipose tissue have been recognized to develop MSCs or HSCs as major candidates for cell-based therapy [47]. However, several logistical issues, such as harvesting the cells through invasive approaches, recovery of cryopreserved cells, and HLA testing to avoid graft vs host disease, limit the potential of these candidates as radiation countermeasures in mass casualty settings. MSCs developed from bone marrow or adipose tissue or induced pluripotent stem cells (iPSCs) are promising candidates for regenerative cell therapy; however, clinical evidence indicates a clear discrepancy between expected and actual outcomes of MSCs taken from bench to bedside [23,24]. Therefore, an alternative cell candidate is needed for radiation countermeasure efforts. In the present study, we showed that hMCPs can overcome all the major challenges of cell-based therapy against acute radiation syndrome. hMCPs developed from human blood PBMCs are prepared from peripheral blood that can be drawn from the donor multiple times non-invasively, vs harvesting by biopsy of the hip or sternum. After expansion, these cells can be stored and recovered even from a cryopreserved state without compromising any functional efficacy. Moreover, hMCPs did not show any GVHD when transplanted in an allogenic setting.

Mice exposed to whole-body irradiation with LD70/30 doses showed improvement in overall survival when treated with hMCPs at 24 h after irradiation. Histopathological and flowcytometric analysis clearly demonstrated the radiation-induced loss of bone marrow cells but recovery with the hMCP transplant. Significant recovery of recipient mice HSCs with hMCP transplant along with improvement in blood lymphocyte count suggested recovery of hematopoietic regeneration. Moreover, the presence of transplanted hMCPs was also noted in these mice even after 28 days of transfusion, suggesting the strong potential of hMCPs for cell-replacement therapy. 

One of the major concerns of cell transplant in the allogenic setting is immunologic graft rejection by the host’s immune system. Presence of allogenic cells can activate allo-reactive T cells through antigen-presenting cells (APCs). Expanded allo-reactive T cells can infiltrate and damage target tissues. hMCPs have shown systemic immuno-suppressive function when transplanted in allogenic mice and mitigate h-ARS without inducing any GVHD in allogenic mice even in later stages (>30 days) post-irradiation. Clinical GVHD has an acute form which may damage the skin, liver, and gastrointestinal tract. Our data clearly showed that hMCP transplant did not promote any acute toxicity in allogenic recipient mice, suggesting the inhibition of GVHD. However, further studies are required to examine the effect hMCP treatment against delayed effect of acute radiation syndrome along with any chronic GVHD symptom.

Recovery of host HSCs and rapid compensation of their functions are critical for bone marrow regeneration and mitigation of h-ARS. While donor progenitor cells can lodge, engraft, and proliferate to repopulate the bone marrow, restitution of host HSCs at early time points post-irradiation can only be achieved by donor cell-derived paracrine signals. Our flowcytometry data clearly showed that mHSCs are also rescued in transplanted mice, suggesting restitution of repair and regeneration of the host HSC population. However, this can only happen through donor cell-derived paracrine signals as transplanted hMCPs are initially lodged into lung. sEVs are the major carrier of cell-derived paracrine signals including regenerative signals, anti-inflammatory signals, anti-oxidative signals in the form of proteins, lipids, and nucleic acids. Our proteomics and sEVs miRNA analysis also demonstrated the presence of exosomal cargo related to the BM regenerative process. Mitigation of h-ARS with hMCP-derived sEVs treatment further confirmed the involvement of paracrine signals in inducing host BM regeneration. Finally, sEVs showing radio-mitigation efficacy were previously cryopreserved and therefore can be considered for a national stockpile. 

Acute radiation syndrome is a complex disease process that may lead to multi-organ syndrome. In recent years, polypharmacy approaches have been gaining more attention over single-agent treatment against such complex multi organ or systemic injury. While identification and optimization of polypharmacy is a challenge, cell, or sEVs-based therapy can perform as an efficient natural polypharmacy as it delivers multiple physiological factors through paracrine signals [48]. Therefore, cell or exosomal treatment enriched with multiple regenerative and immunomodulatory factors should be considered as a leading radio-mitigation approach.

## 4. Materials and Methods

### 4.1. Human Myeloid-Committed Progenitor Cell (hMCP) Culture

Human myeloid progenitor cells were cultured from human PBMCs purchased from StemCell Technologies (Cat no. 70025.3) and plated in T75 flasks. After 24 h of incubation, cells were transferred to a fresh flask and cultured for seven days with expansion media containing StemSpan™-XF with addition of StemSpan™ CD34+ Expansion Supplement (StemCell Technology, Vancouver, BC, Canada). Cells were collected after 14 days of expansion and characterized by flowcytometry analysis. 

### 4.2. Characterization of hMCP

Expanded cells were characterized for myeloid-committed progenitor cell expression markers. During cell expansion, cells were collected on day 7 and day 14 and analyzed for expression of myeloid-committed progenitor cell genes (*CD33, NRG-3, Tnfrs-11a*) [49,50,51] by qPCR. RNA was extracted from day 7 and 14 expanded cells using RNeasy Mini Kit (Qiagen, Germantown, MD, USA). The concentration and purity of the extracted RNA were checked using a NanoDrop spectrometer (Thermo Scientific, Waltham, MA, USA). 1 µg of total RNA was reverse-transcribed using RNA to cDNA EcoDry™ Premix (Double Primed) (Takara Bio USA Inc., San Jose, CA, USA). The reaction mixture was incubated for 1 h at 42 °C; incubation was stopped at 70 °C for 10 min. Quantitative real-time PCR (qPCR) was performed using the QuantStudio™ 7 Flex Real-Time PCR System (Applied Biosystems™, New York, NY, USA) and SYBR Green Supermix (Bio-Rad, Hercules, CA, USA) with specific primers to the target genes in a 20 µL final reaction volume. The primer sequences are listed in Supplementary Table S1. Beta-actin was used as a reference gene for sample normalization. The delta-delta threshold cycle (ΔΔCt) method was used to calculate the fold change expression in mRNA level in the samples.

Further, the phenotype of the expanded cells was characterized by flowcytometric analysis using the following antibodies: BV605 CD34 (Clone 581, BioLegend, San Diego, CA, USA), BV711 CD45RA (Clone HI100, BD Biosciences), BV510 Lineage (BioLegend, 348807), APC CD38 (Clone REA 572, MACS), PE CD123 (Clone AC145, MACS), PE/Cy7 CD135 (Clone BV10A4H2, BioLegend) [52]. According to previous reports, myeloid-committed progenitors are Lin-CD45RA-CD34+CD38+ cells with the highest myeloid colony forming efficiency compared with Lin-CD45RA-CD34+CD38− cells [53]. The percentage of Lin-CD45RA-CD34+CD38+ cells was measured in the total cell population. Further, the percentage of Lin-CD45RA-CD34+CD38+ cells expressing the myeloid-committed cell markers CD123, CD135/Flt3 was measured to determine hMCP cell population. We did not observe any significant difference in surface markers in hMCPs derived from different batches of PBMC. The flowcytometry data were analyzed by FlowJo software (BD Biosciences, Franklin Lakes, NJ, USA).

### 4.3. Collection of hMCP-Conditioned Media

For collection of hMCP-conditioned media, cells were cultured at a density of 16–18 × 10^3^ cells per cm^2^ in T175 cm^2^ flasks with 20 mL of cell expansion media. After 4 days of expansion, cells were washed with PBS two to three times followed by a final wash with supplement free Basal media for complete removal of cell expansion medium and antibiotics. Over the next 24 h these hMCPs (52 × 10^3^ cells per cm^2^) were incubated with supplement-free Basal media. After 24 h, conditioned media were collected for further in vivo and in vitro applications. Cells incubated over 24 h without growth supplement showed 82.4% viability with the concentration of 42.8 × 10^3^ cells per cm^2^. Conditioned media collected from different flasks from passage 4 and 5 pooled together and centrifuged at 500× *g* for 10 min at 4 °C to remove the cells. Cell-free conditioned media was frozen at −80 °C until further use.

### 4.4. Isolation of Small Extracellular Vesicles (sEVs) from hMCP-Conditioned Media

sEVs were isolated from hMCP-conditioned media using a differential centrifugation method as described previously [54]. A detailed schematic diagram for sEVs isolation from hMCP-conditioned media using the Ultracentrifugation method is described in Supplementary Figure S16. In brief, the conditioned media from hMCP cells was centrifuged at 500× *g* for 10 min at 4 °C to remove the cells. The supernatant was centrifuged at 2000× *g* for 10 min at 4 °C to remove any leftover dead cells. To remove the apoptotic bodies/debris, the supernatant was centrifuged at 10,000× *g* for 30 min at 4 °C. The supernatant was then subjected to ultracentrifugation at 100,000× *g* for 70 min at 4 °C. The pellet-containing sEVs were washed with cold PBS at 100,000× *g* for 70 min at 4 °C. The sEVs exosome pellet was resuspended in PBS and stored at −80 °C for further studies. 

### 4.5. Characterization of sEVs

sEVs were characterized by flowcytometry by using FITC CD9 (clone HI9a, BioLegend), Pe CD81 (clone 5A6, BioLegend), APC CD63 (clone H5C6, BioLegend) antibody cocktail in 30 µL of suspension and incubated 20 min at 4 °C in the dark. After incubation, the sEVs were washed twice with PBS and transferred to the flowcytometry tube for analysis. Flowcytometric data were analyzed by FlowJo software.

### 4.6. sEVs Protein Estimation and Particle Count

The total protein concentration of isolated sEVs was determined using the micro bicinchoninic acid (BCA) assay kit (Thermo Scientific, Waltham, MA, USA) according to the manufacturer’s instructions. Briefly, the isolated sEVs were diluted 1:80 using distilled water and 150 µL of a BCA mixture of reagent A, B, and C (A:B:C  =  25:24:1) was added and incubated for 2 h at 37 °C. The optical density (OD) of the sample was measured at 562 nm on the Infinite 200 PRO multimode plate reader (Tecan Group Ltd., Männedorf, Switzerland). The protein concentration was calculated from a standard BCA curve. All measurements were carried out under constant experimental conditions to obtain comparable results.

### 4.7. Nanoparticle Tracking Analysis (NTA) of sEVs

Size distribution and concentration of isolated sEVs were measured by Nanosight NTA (LM10, Malvern Inst. Ltd., Malvern, UK). The NTA analyzes the motion of particles illuminated by a laser, from which it deduces their size and concentration. sEVs samples were diluted (1:10) with PBS and readings were taken in triplicates.

### 4.8. Exosomal miRNA Analysis

Purified sEVs were subjected to RNA isolation using the exoRNeasy kit (QIAGEN, USA) according to the manufacturer’s protocol. Quality of the isolated EV-derived RNA was assessed using an Agilent 2100 Bioanalyzer (Agilent Technologies Inc., Santa Clara, CA, USA). The Nano String nCounter miRNA expression assay was then used to measure the levels of ~800 miRNAs following the manufacturer’s protocol. This assay provides a direct, digital count of each miRNA without the use of reverse transcription or PCR-based amplification. Briefly, 50 ng of the EV-derived RNA was used for the assay. Following overnight hybridization at 65 °C with the Reporter Code Set and Capture ProbeSet, the samples were further processed on the automatic Prep Station to remove excess reporter probes and non-target cellular transcripts followed by removal of excess capture probes. The purified target/probe complexes were then immobilized on the cartridge for data collection via digital imaging on the Digital Analyzer. Barcode counts were tabulated in a comma separated value (CSV) format and analyzed using the nSolver™ software (ver. 3.0) to assess data quality and perform data normalization using the Top 100 normalization method built into the software. Normalized miRNA data obtained from these assays were analyzed using miRNA database online software miRPathDB 2.0 (https://mpd.bioinf.uni-sb.de/overview.html) accessed on 12 August 2021 to predict their related function and target genes. From the total number of miRNAs identified, a cutoff was made based on count number from the Nano String assay. MicroRNAs with a count number >10 were further analyzed using Gene Ontology (GO) annotation and Kyoto Encyclopedia of Genes and Genomes (KEGG) pathway analysis.

### 4.9. T Cells Inhibition Assay

To confirm the immunosuppressive capacity of cultured hMCPs, we performed T cell inhibition assays as described previously using the suppressive role of mesenchymal stem cells [55]. The cultured hMCPs were plated at a density of 1 X 10^5^ cells per well of 24 well plates along with 1 × 10^6^ PBMCs with 25 μL of T Cell-activating beads (ImmunoCult™ Human CD3/CD28 T Cell Activator Cat no 10970, StemCell Technology) per mL of media. The culture was incubated at 37 °C with 5% CO_2_ for 72 h. After incubation, the secretion of proinflammatory cytokines (IFN-γ and TNF-α) and surface marker (CD25) of T cells were analyzed by flowcytometric analysis.

### 4.10. Mice

10–12 week old male *NOD.Cg-Rag1^tm1Mom^ Il2rg^tm1Wjl^/SzJ* (*NRG* mice) (Stock #7799, Jackson laboratories, Bar Harbor, ME, USA) and 10–12 week old male *C;129S4-Rag2^tm1.1Flv^ Il2rg^tm1.1Flv^/J* (*Rag2-γc*-mice) (Stock #014593, Jackson laboratories) immunocompromised mice were used in the present study. Both these immunocompromised mice do not have the Prkdc mutation, which makes them a less radiosensitive strain compared with other immunocompromised mice for the use of cells engraftment studies. However, there are moderate differences in radiosensitivity between these two strains. To examine the efficacy of hMCP transplantation in an allogenic setting, we used 8–10 week old non-immunocompromised male FVB mice (Stock#01800, Jackson laboratories). All of the experimental mice were maintained in a pathogen-free environment and housed in cages in groups of five animals per cage with constant temperature and humidity and a 12 h/12 h light/dark cycle. All animals always had access to food (8604; Teklad Rodent Diet) and water. All of these animal studies were performed under the guidelines and protocols of the Institutional Animal Care and Use Committee of the University of Kansas Medical Center (ACUP number 2019-2487).

### 4.11. Irradiation

An irradiation procedure was performed on mice anesthetized with 87.5 mg/kg of Ketamine and 12.5 mg/kg of Xylazine using a small animal radiation research platform (XENX; XStrahl, Suwanee, GA, USA) as previously described [56] at a dose rate of 2.26 Gy/min at 220 V and 13 mA. To ensure homogeneous dose delivery, half of the dose was delivered from the anterior–posterior direction and the other half from the posterior–anterior direction. The output of the X-ray irradiator was verified using an ion chamber measurement detector system to confirm the dose rate of the irradiator. Based on the calculated dose and the dose rate, the time of irradiation was calculated to meet the required radiation dose. Dosimetry was routinely performed by a physicist from the Department of Radiation Oncology, University of Kansas Medical Center, Kansas City, KS, 66160, USA. 

### 4.12. Assessment of Graft Vs Host Disease (GVHD)

*FVB* mice treated with/without hMCPs were monitored daily from day 1 until day 14 for the clinical manifestations of GVHD (for example, weight loss, hunched posture, poor activity, ruffled fur) [57]. After 14 days, animals were euthanized and blood and spleen were collected for immune cell analysis while skin, liver, and colon were collected for histological analysis of GVHD assessment.

### 4.13. Bone Marrow Cell Analysis

Cells were harvested from femurs and tibias. Red blood cells were lysed using 0.16 M ammonium chloride. Cells were stained using the following antibodies: FITC lineage, BioLegend (CD3e, clone 145-2C11, CD4, clone GK1.5, CD8a, clone 53-6.7, B220, IgM, clone RMM-1, Mac-1, clone M1/70, Gr1, clone RB6-8C5, Ter119 respectively); PE-Cy7 Sca-1 (clone D7, BioLegend); APC c-Kit (clone ACK2, BioLegend; BV711 CD150 (clone TC15-12F12.2, BioLegend); PerCP Cy5.5 CD48 (clone HM48-1, BioLegend); BV421 human CD45 (clone HI30, BioLegend) [58]. Flow cytometry analysis was performed using a custom LSR Fortessa X-20 analyzer (BD Biosciences). Data analysis was performed using FlowJo software (BD Biosciences). Mouse HSCs were defined as lineage negative, Sca-1+, c-Kit+, CD150+, CD48− cells. Cellularity was based on the total nucleated cell count from 1 femur + 1 tibia. 

### 4.14. Histology of Bone Marrow

Bones were fixed in 10% neutral-buffered formalin (G Biosciences) followed by decalcification. After decalcification, bones were processed for paraffin embedding. Then, 5 μm sections were cut and placed on the slides for hematoxylin and eosin (H&E) staining. The H&E slides from all the groups were examined by light microscopy to capture bright-field images using EVOS XL core microscope.

### 4.15. Ex Vivo Colony-Forming Unit (CFU) Assay

Human Bone-marrow derived CD34+ cells were kindly gifted by Ossium Health (Indianapolis, IN, USA) for performing CFU assay. Cells were plated in 96-well plate with StemMACS HSC-CFU assay media (Miltenyi Biotec Inc., Auburn, CA, USA) and cultured for 14 days according to the manufacturer’s protocol. All of the plates were incubated at 37 °C with 5% CO_2_ for 14 days. After incubation, colonies were stained with a staining cocktail of fluorochrome-conjugated antibodies: CD235a antibody, anti-human, PE, REAfinity™ (clone: REA175, isotype: recombinant human IgG1), CD15 antibody, anti-human, APC (clone: VIMC6, isotype: mouse IgM), CD14 antibody, anti-human, VioBlue^®^ (clone: TÜK4, isotype: mouse IgG2aκ) as per the manufacturer’s protocol and subjected to flowcytometric analysis.

### 4.16. Proteomics Analysis of hMCP-Conditioned Media

Conditioned media from expanded hMCPs were collected and centrifuged at 500× *g* for 10 min to remove any cell debris. Supernatants were stored at −80 °C until they were sent to BGI Global genomic services, San Jose, USA for mass spectrophotometry. Growth medium used for cell culture were considered as background control. All the MS data were further analyzed using Ingenuity Pathway Array assist software.

### 4.17. Statistical Analysis

A comparison between groups was carried out by nonparametric T-test (Mann–Whitney test) using GraphPad Prism 9 (GraphPad Software, San Diego, CA, USA). Kaplan–Meier Survival analysis of mice survival/mortality in different treatment groups was analyzed by Kaplan–Meier (Mantel–Cox) as a function of radiation dose using GraphPad Prism. All data are presented as mean ± standard deviation (SD). A difference between groups with * *p* < 0.05, ** *p* < 0.005, or *** *p* < 0.0005 was considered statistically significant.

## 5. Conclusions

This study established hMCPs as a major therapeutic candidate to mitigate h-ARS. These cells can be transplanted to allogenic recipients, suggesting their applicability in mass casualty settings. Moreover, our data clearly suggest that hMCPs mitigates h-ARS by inducing repair and regeneration of host HSCs as well as repopulating the bone marrow by self-renewal and proliferation. We were able to identify several proteins and miRNAs responsible for the activation of regenerative process. hMCP should be considered as a major candidate for polypharmacy against h-ARS. To advance our cell-based therapy for clinical applications, further studies are needed in large animal models where hMCPs will be tested in combination with existing standard-of-care treatments against h-ARS. 

## Figures and Tables

**Table 1:** Small extracellular vesicles (sEVs)-miRNA involved in various biological functions of Hematopoietic Stem Cells including proliferation, homeostasis, cell lineage, differentiation, and regulation of differentiation using their target genes.

Identified miRNA	*p*-Value	Counts	Target Genes
Hematopoietic Stem Cell Proliferation			
hsa-miR-22-3p	0.007	52	CTC1, MECOM, WNT1
hsa-miR-148a-3p	0.017	11	WNT1, WNT10B, WNT2B
hsa-miR-1246	0.029	2038	CTC1, PIM1
hsa-miR-19b-3p	0.012	69	ARIH2, ATXN1L, CD34, EIF2AK2, MECOM, N4BP2L2, SFRP2, THPO, WNT1, WNT2B
hsa-miR-495-3p	0.033	11	ACE, ARIH2, ATXN1L, CTC1, EIF2AK2, MECOM, N4BP2L2, NKAP, RUNX1, SFRP2, WNT2B
hsa-miR-765	0.040	83	ACE, ARIH2, ATXN1L, CD34, EIF2AK2, ETV6, NKAP, PIM1, RUNX1, WNT1, WNT10B, WNT2B
hsa-miR-582-5p	0.004	12	ACE, ARIH2, ATXN1L, CTC1, EIF2AK2, MECOM, N4BP2L2, NKAP, PDCD2, PIM1, RUNX1, SFRP2, THPO, WNT1, WNT10B, WNT2B
hsa-miR-1915-3p	0.018	613	ACE, ARIH2, ATXN1L, CD34, CTC1, EIF2AK2, ETV6, N4BP2L2, NKAP, PDCD2, PIM1, RUNX1, SFRP2, THPO, WNT1, WNT10B, WNT2B
hsa-miR-570-3p	0.031	11	ARIH2, ATXN1L, CD34, CTC1, EIF2AK2, ETV6, MECOM, N4BP2L2, NKAP, PDCD2, PIM1, RUNX1, SFRP2, WNT1, WNT10B, WNT2B
hsa-miR-148b-3p	0.034	18	ACE, ARIH2, ATXN1L, CTC1, EIF2AK2, ETV6, MECOM, N4BP2L2, PDCD2, RUNX1, THPO, WNT1, WNT10B, WNT2B
hsa-miR-4516	0.046	2637	ACE, ARIH2, ATXN1L, CD34, CTC1, EIF2AK2, ETV6, N4BP2L2, NKAP, PDCD2, PIM1, RUNX1, THPO, WNT1, WNT10B, WNT2B
Hematopoietic Stem Cell Homeostasis			
hsa-miR-130a-3p	0.044	13	CCN3, NLE1, TCIRG1, UBAP2L
Hematopoietic Cell Lineage			
hsa-miR-451a	0.010	10	IL6, IL6R
hsa-miR-34a-5p	0.013	16	CD24, CD44, CSF1R, IL6R, ITGA6, ITGB3, KIT, TFRC, TNF
hsa-miR-148b-3p	0.022	18	CSF1, ITGA5
hsa-miR-34a-5p	0.031	16	CD24, CD44, CSF1R, IL6R, KIT
hsa-miR-451a	0.035	10	IL6, IL6R
Hematopoietic Stem Cell Differentiation			
hsa-miR-223-3p	0.007	46	IL6, LMO2, STAT5A
hsa-miR-146a-5p	0.008	66	CXCR4, FOS, IL6, NOTCH1
hsa-miR-184	0.020	24	CSF1, NFATC2
hsa-miR-34a-5p	0.028	16	FOS, KCNH2, LEF1, MYB, NOTCH1
hsa-miR-223-3p	0.041	46	IL6, LMO2, STAT5A
hsa-miR-155-5p	0.044	268	FLI1, FOS, IL6, MXI1, MYB, SPI1, THRB
hsa-miR-34a-5p	0.047	16	FOS, ITGB3, KCNH2, LEF1, MYB, NOTCH1
Regulation of Hematopoietic Stem Cell Differentiation			
hsa-miR-615-3p	0.048	13	CBFB, CDK6, HSPA9, PSMB7, PSMD13, PSMD2, PSMD8, PSMF1, TCF3, YTHDF2
hsa-miR-105-5p	0.018	17	CDK6, MYB

## Data Availability

Data is contained within the article and Supplementary Material.

## Supplementary Materials

The following supporting information can be downloaded at: https://www.mdpi.com/article/10.3390/ijms23105498/s1.
