# Supplementary material for: Human Peripheral Blood Mononucleocyte Derived Myeloid Committed Progenitor Cells Mitigate H-ARS by Exosomal Paracrine Signal
Source: Int J Mol Sci. 2022 May 14;23(10):5498. doi: 10.3390/ijms23105498 (PMC9142131; doi:10.3390/ijms23105498)
Supplement: Supplementary file 1 [file ijms-23-05498-s001.zip › ijms-1687506-supplementary.pdf]

Supplementary Figure: 1

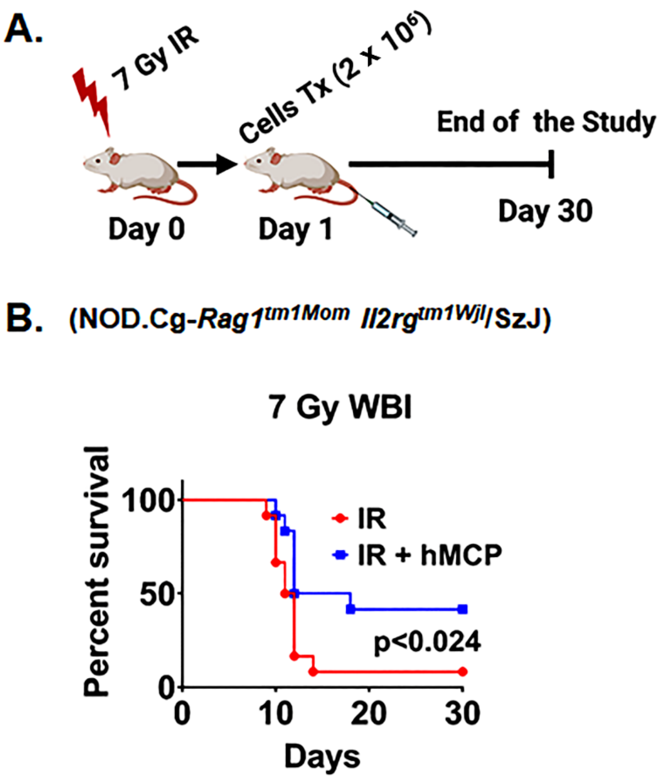

Supplementary Figure S1: Single dose of hMCP cell transplantation in mice following whole body irradiation. (A) Schematic representation of survival study experimental plan (radiation dose and timeline for cell transplantation). Mice receiving hMCP cells at 24 hours after irradiation (B) Kaplan-Meier survival (Mantel-Cox) analysis of *NOD.Cg-Rag1tm1Mom Il2rgtm1Wjl/SzJ* (NRG) mice showed 41.6% of survival in IR+hMCP single dose transplanted group compared to IR group. ($p<0.024$). (n = 12 mice per group).

Supplementary Figure: 2

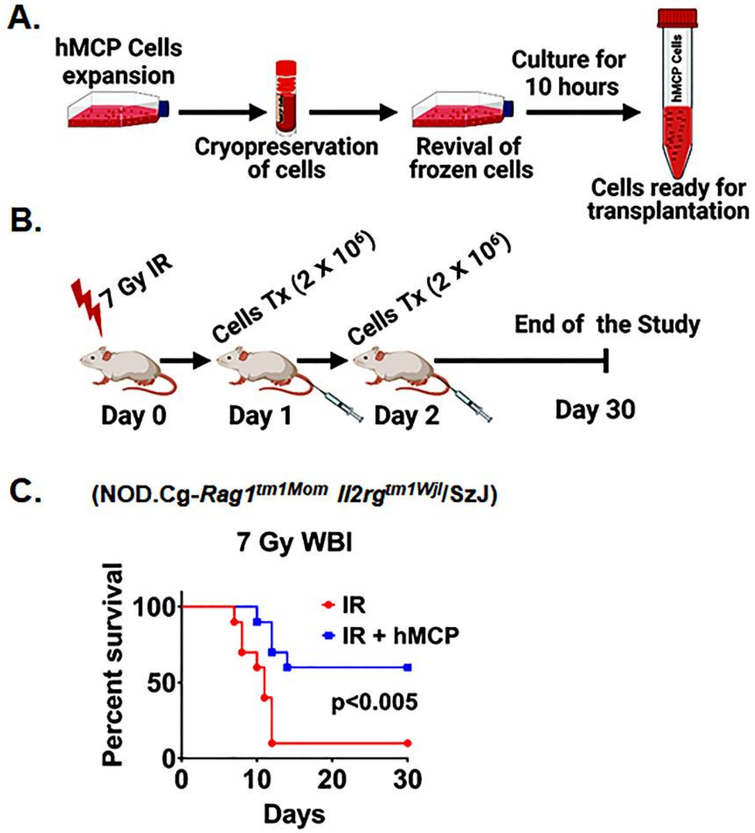

Supplementary Figure S2: Functional efficacy of frozen and thawed hMCP cell transplantation improves survival of mice following whole body irradiation. (A) Schematic representation of hMCP cells preparation for transplantation in mice after cryopreservation and revival of cells (B) Survival study experimental plan (radiation dose and timeline for cell transplantation). Mice receiving hMCP cells prepared from cryopreserved vials at 24 and 48 hours after irradiation (C) Kaplan-Meier survival analysis of *NOD.Cg-Rag1tm1Mom Il2rgtm1Wjl/SzJ* (NRG) mice showed 60% of survival ($p<0.005$) in IR+hMCP transplanted group compared to IR group (10% survival). (n = 10 mice) per group.

Supplementary Figure: 3

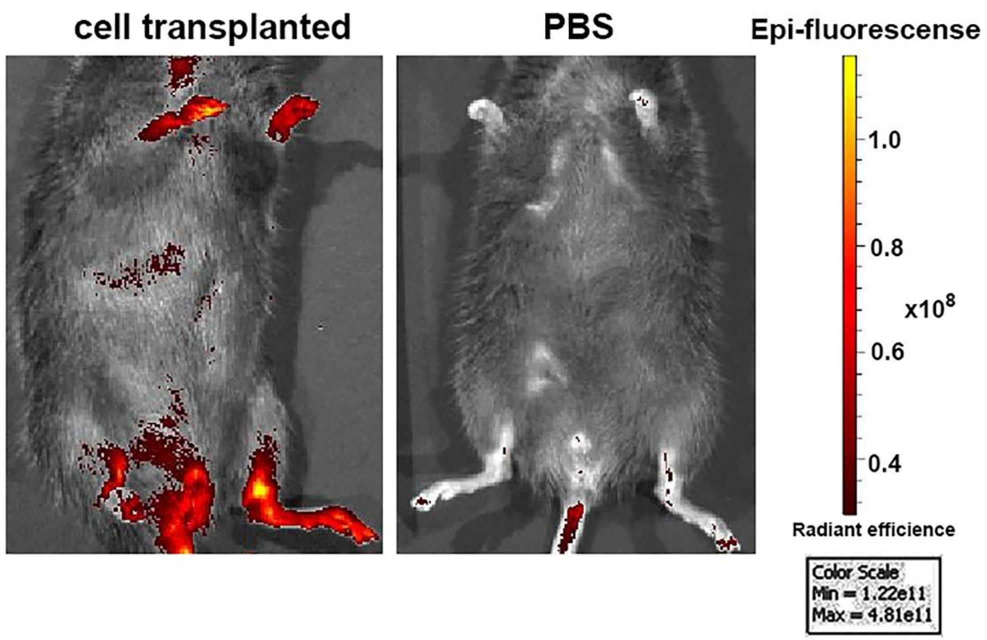

Supplementary Figure S3: In-vivo imaging system shows hMCP cells primarily located into lung and bone-marrow of mice.

Supplementary Figure: 4

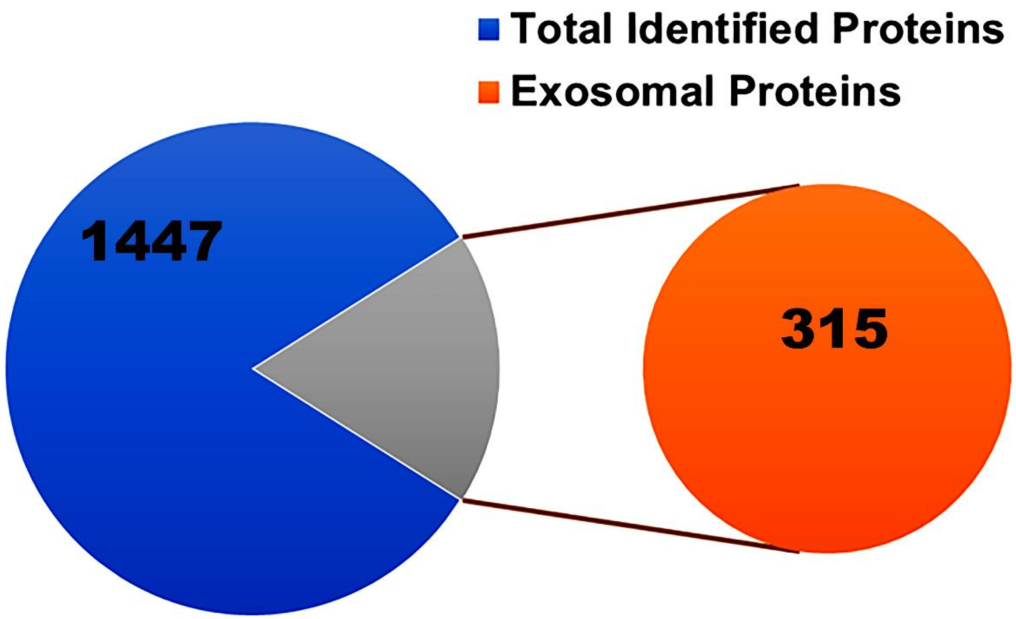

Supplementary Figure S4: Proteomics analysis of hMCP conditioned media shows that out of 1447 total identified proteins, 315 (21.8%) are exosomal proteins.

Supplementary Figure: 5

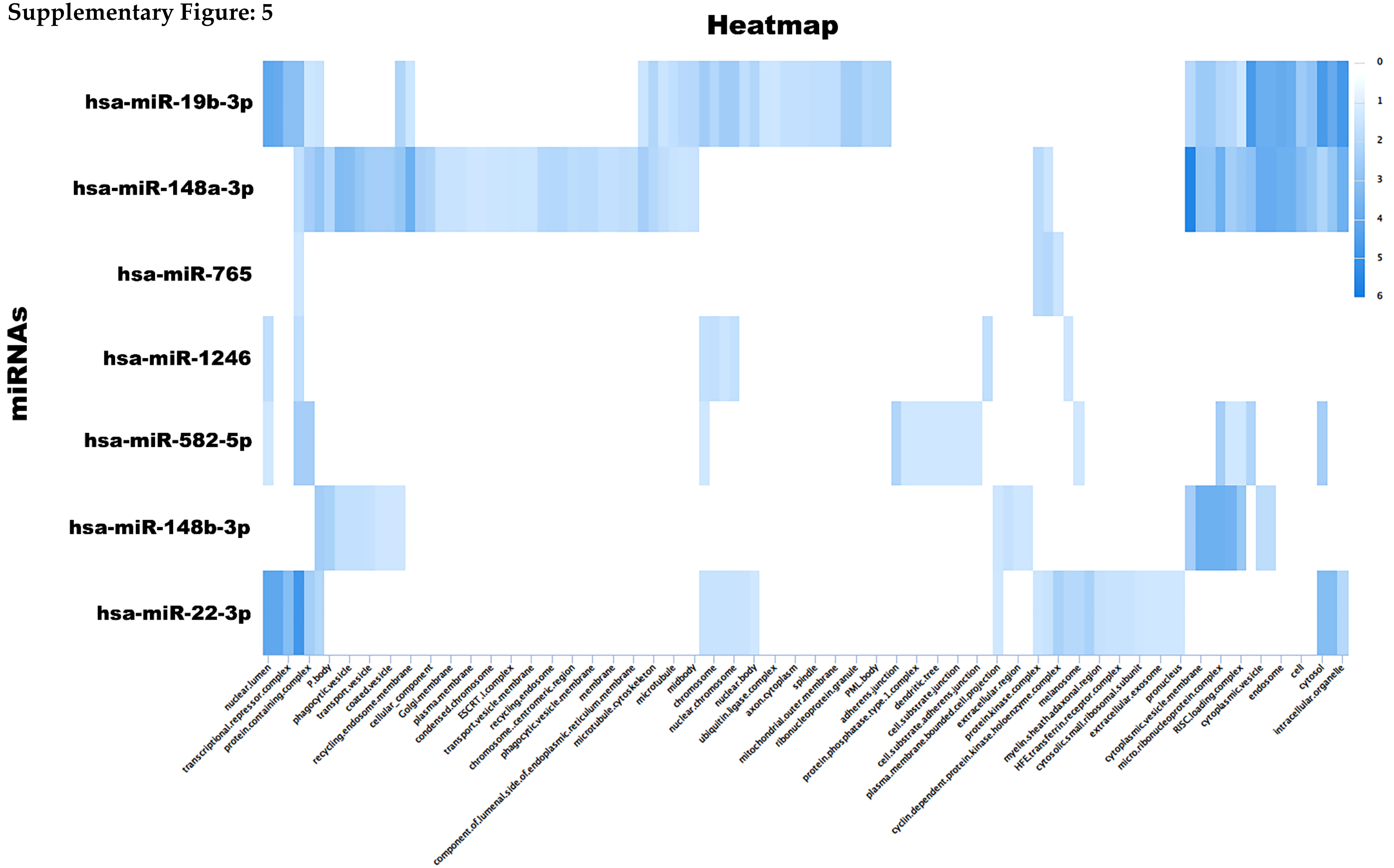

Supplementary Figure S5: Gene Ontology (GO) analysis of shortlisted exosomal-miRNA analyzed by miRPathDB v2.0. revealed various cellular components involved in HSCs Proliferation.

Supplementary Figure: 6

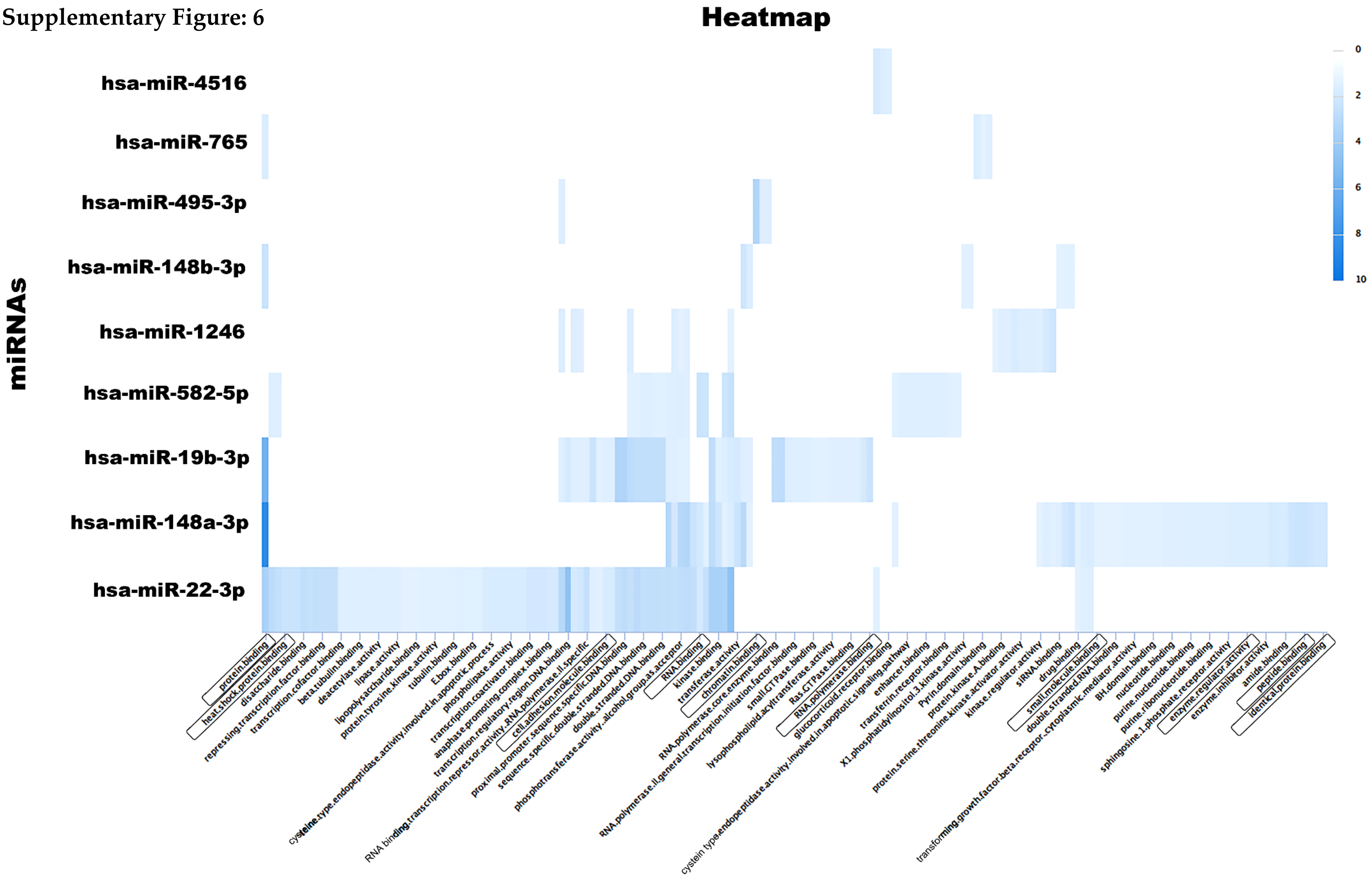

Supplementary Figure S6: Gene Ontology (GO) analysis of shortlisted exosomal-miRNA analyzed by miRPathDB v2.0. revealed various important molecular functions (highlighted by rectangular box) such as protein binding, heat shock protein binding, cell adhesion molecule binding, RNA binding, RNA polymerase binding, enzyme regulator activity etc. involved in HSCs Proliferation.

Supplementary Figure: 7

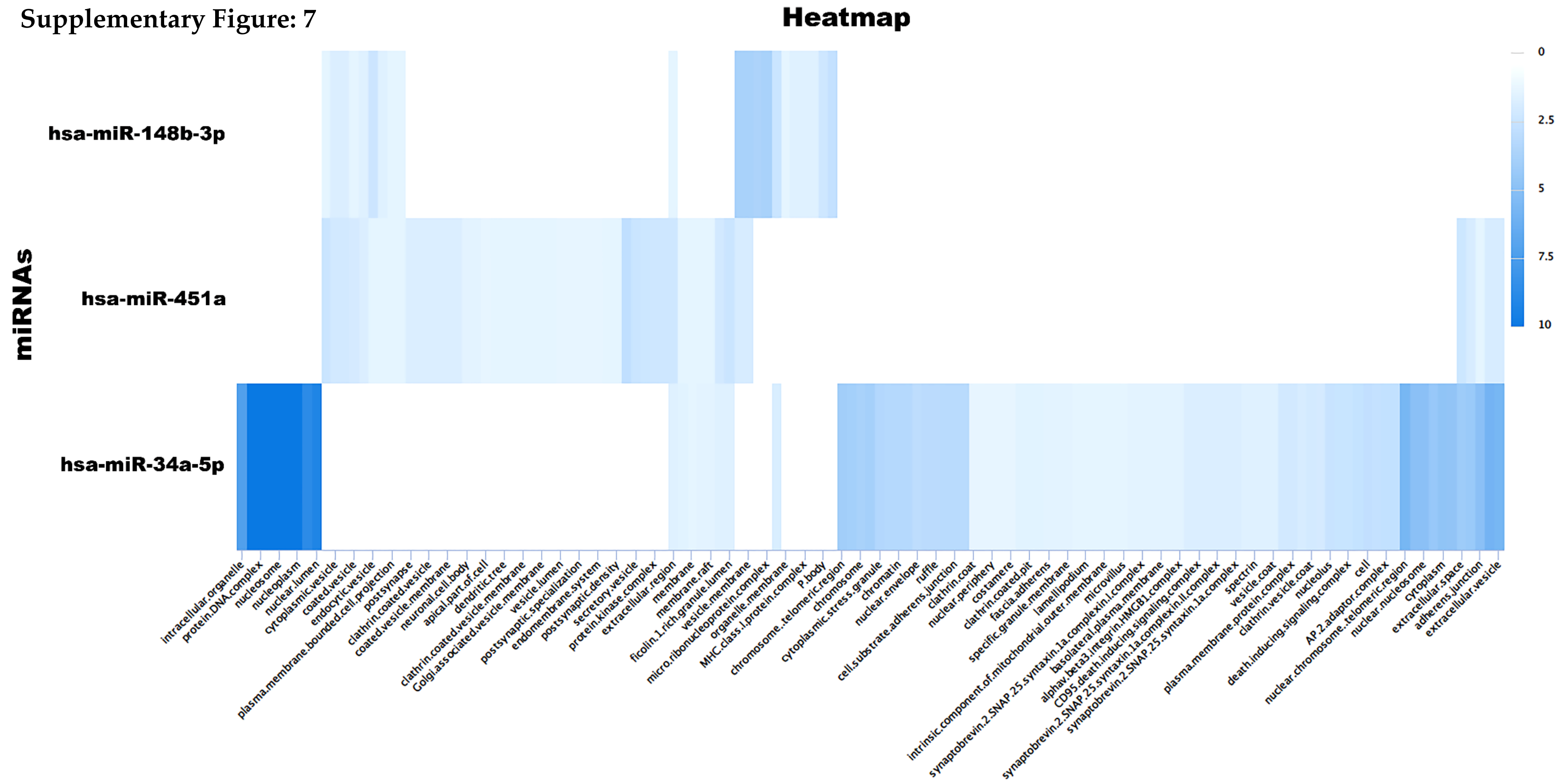

Supplementary Figure S7: Gene Ontology (GO) analysis of shortlisted exosomal-miRNA analyzed by miRPathDB v2.0. revealed various cellular components involved in HSCs lineages.

Supplementary Figure: 8

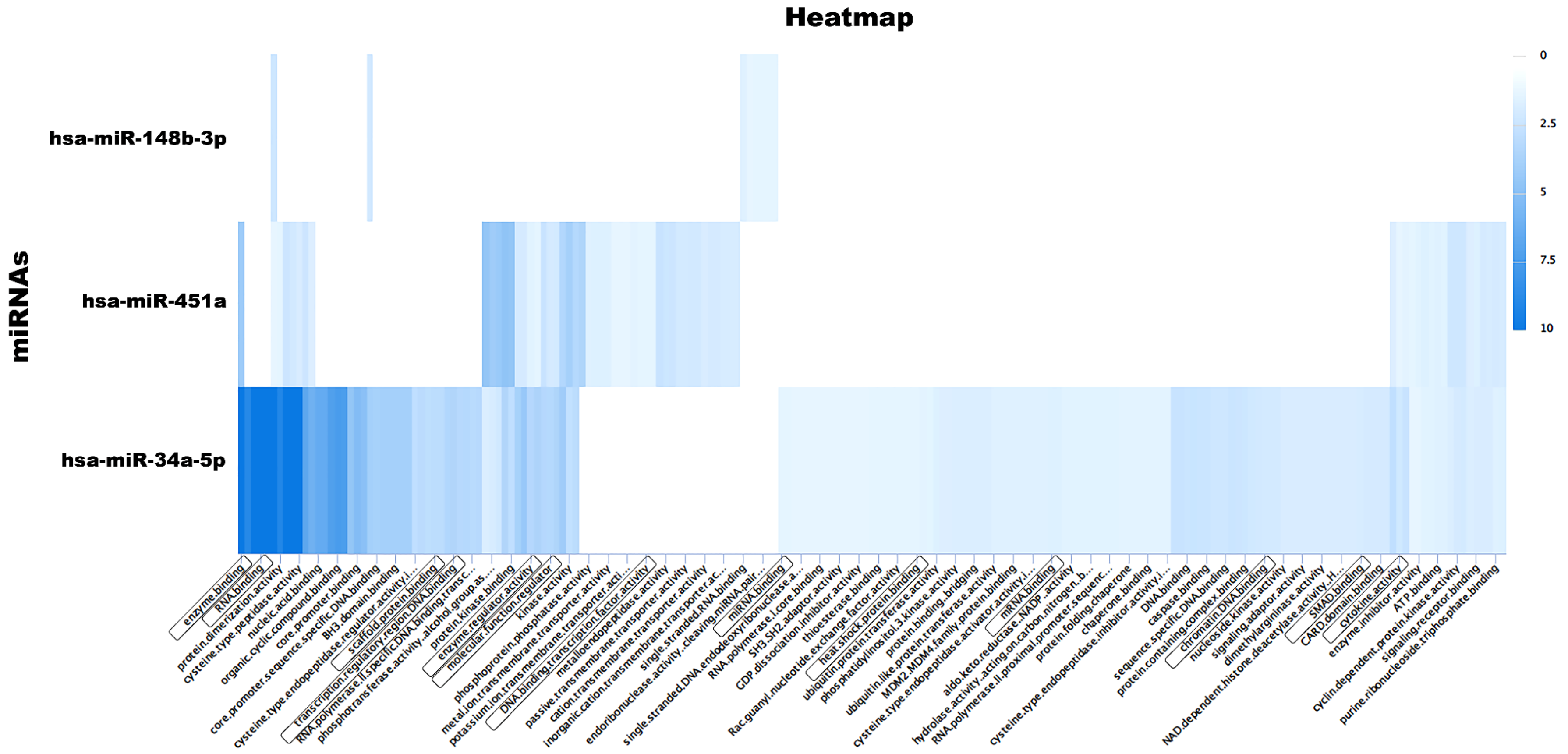

Supplementary Figure S8: Gene Ontology (GO) analysis of shortlisted exosomal-miRNA analyzed by miRPathDB v2.0. revealed various important molecular functions (highlighted by rectangular box) such as enzyme binding, RNA binding, scaffold protein binding, molecular function regulator, miRNA binding, heat shock protein binding, mRNA binding, chromatin DNA binding, cytokine activity etc. involved in HSCs lineages.

Supplementary Figure: 9

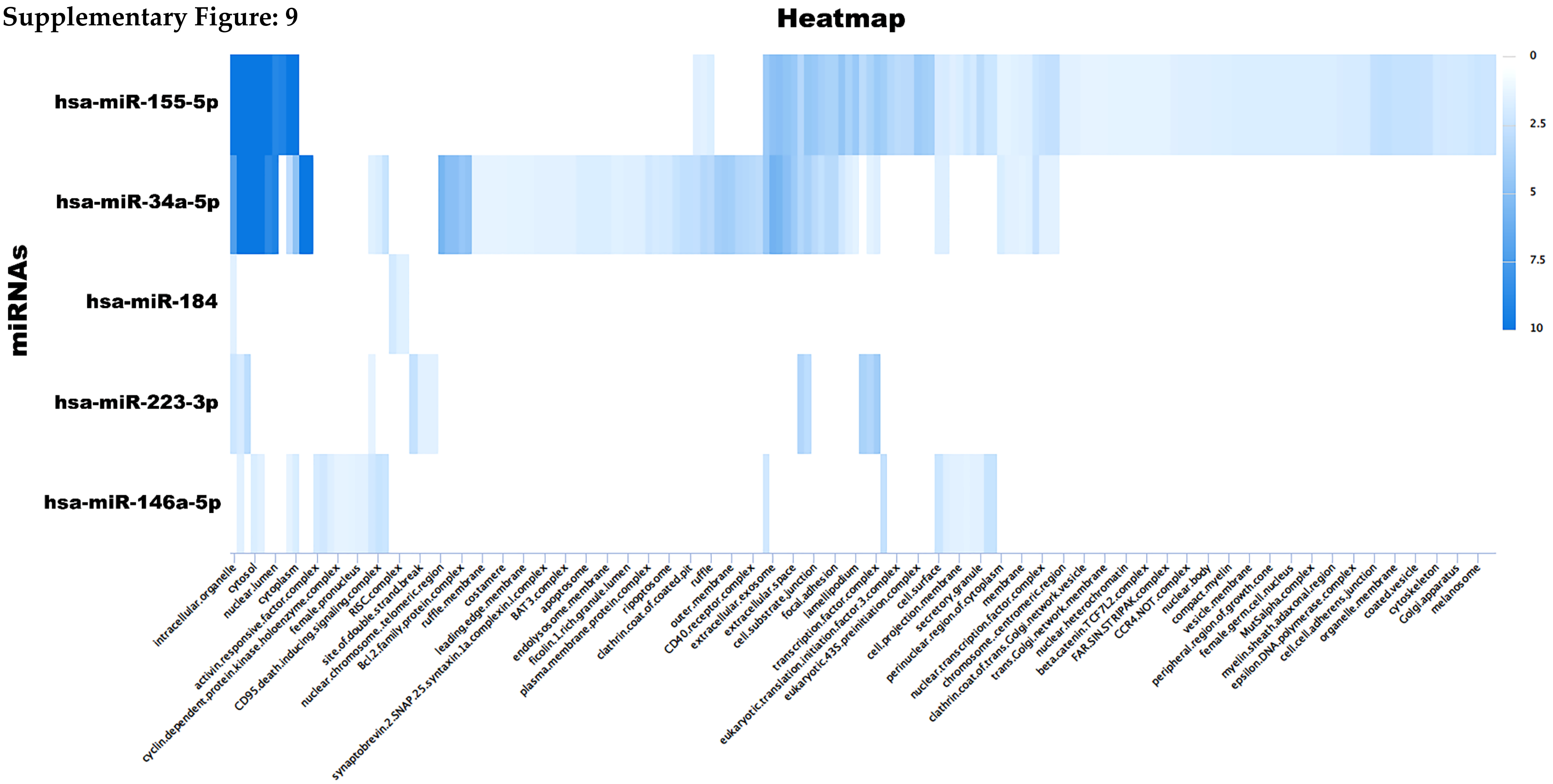

Supplementary Figure S9: Gene Ontology (GO) analysis of shortlisted exosomal-miRNA analyzed by miRPathDB v2.0. revealed various cellular components involved in HSCs differentiation.

Supplementary Figure: 10

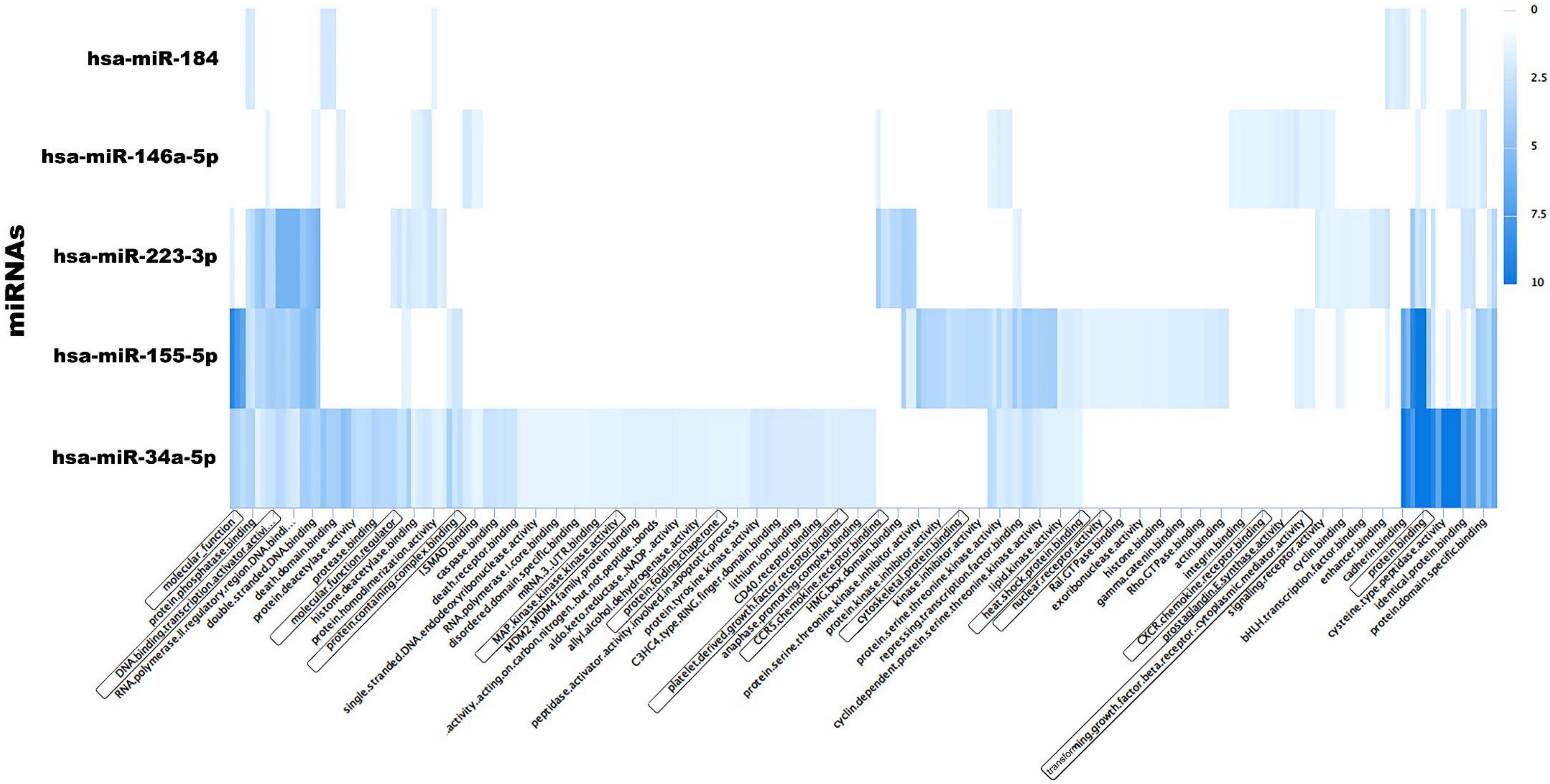

Supplementary Figure S10: Gene Ontology (GO) analysis of shortlisted exosomal-miRNA analyzed by miRPathDB v2.0. revealed various important molecular functions (highlighted by rectangular box) such as DNA binding transcription activator activity, molecular function regulator, protein containing complex binding, MAP kinase activity, platelet derived growth factor receptor binding, CCR5 chemokine receptor binding, cytoskeletal protein binding, heat shock protein binding, CXCR chemokine receptor binding, transforming growth factor beta receptor cytoplasmic mediator activity etc. involved in HSCs differentiation.

Supplementary Figure: 11

Heatmap

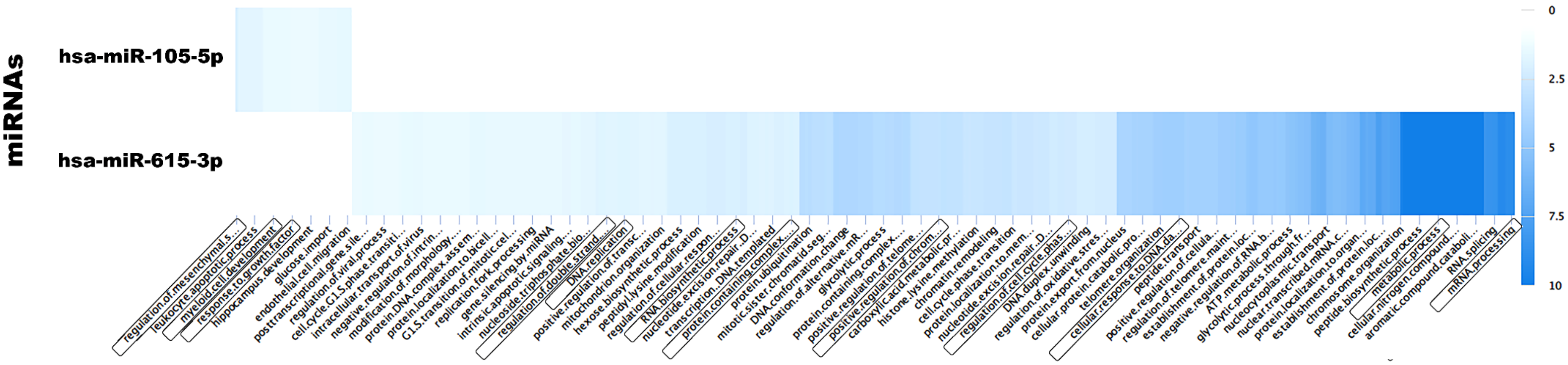

Supplementary Figure S11: Gene Ontology (GO) analysis of shortlisted exosomal-miRNA analyzed by miRPathDB v2.0. revealed various biological functions (highlighted by rectangular box) such as myeloid cells development, response to growth factor, DNA replication, RNA biosynthetic process, protein containing complex, positive regulation of chromatin, cellular response to DNA damage, metabolic process, mRNA processing etc. involved in regulation of HSCs differentiation.

Supplementary Figure: 12

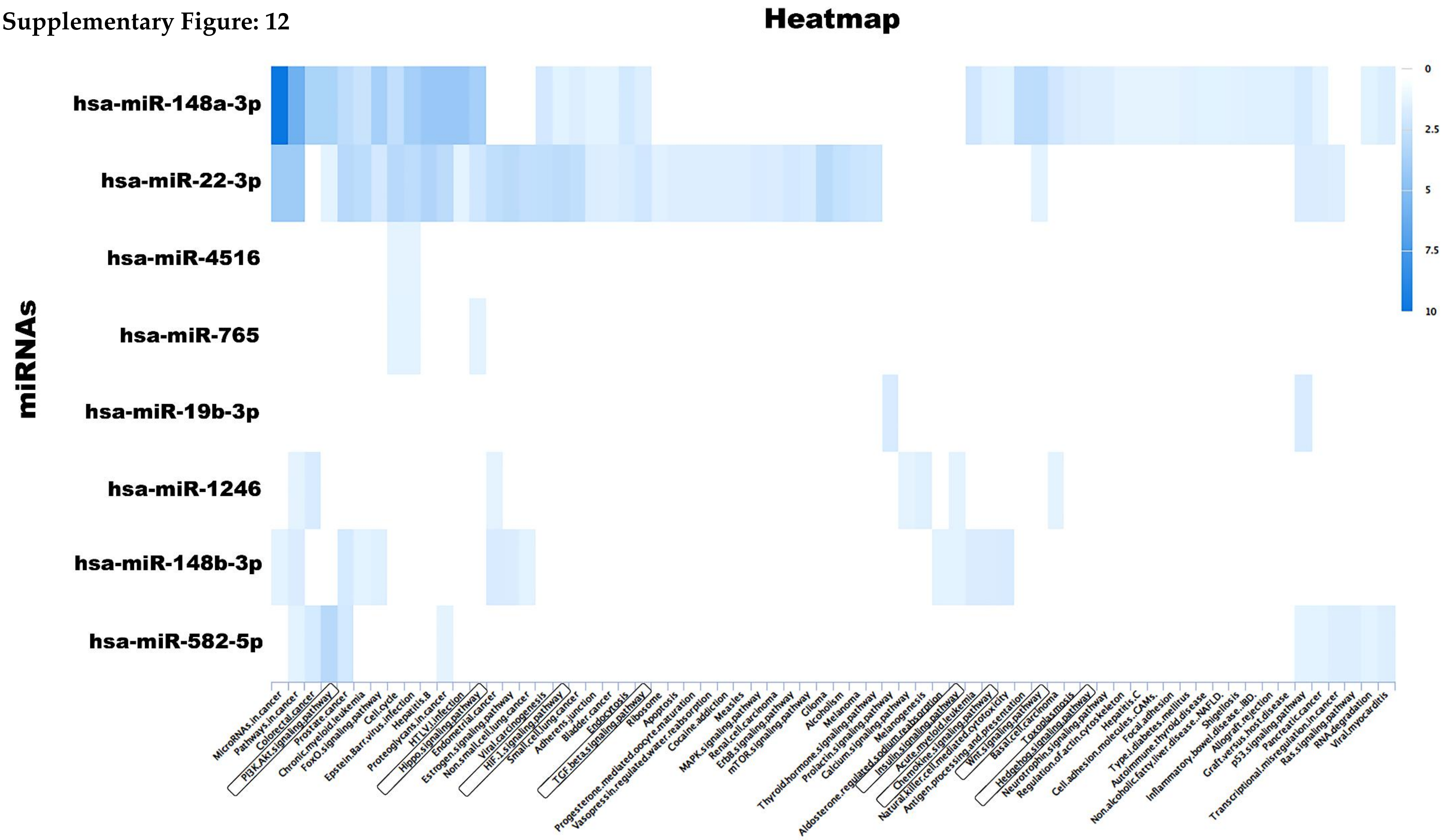

Supplementary Figure S12: KEGG analysis of shortlisted exosomal-miRNA analyzed by miRPathDB v2.0 revealed target signaling pathways (highlighted by rectangular box) such as PI3K-Akt signaling pathway, Hippo signaling pathway, HIF-1 signaling pathway, TGF- β signaling pathway, insulin signaling pathway, Wnt signaling pathway, Hedgehog signaling pathway etc. involved in HSCs proliferation.

Supplementary Figure: 13

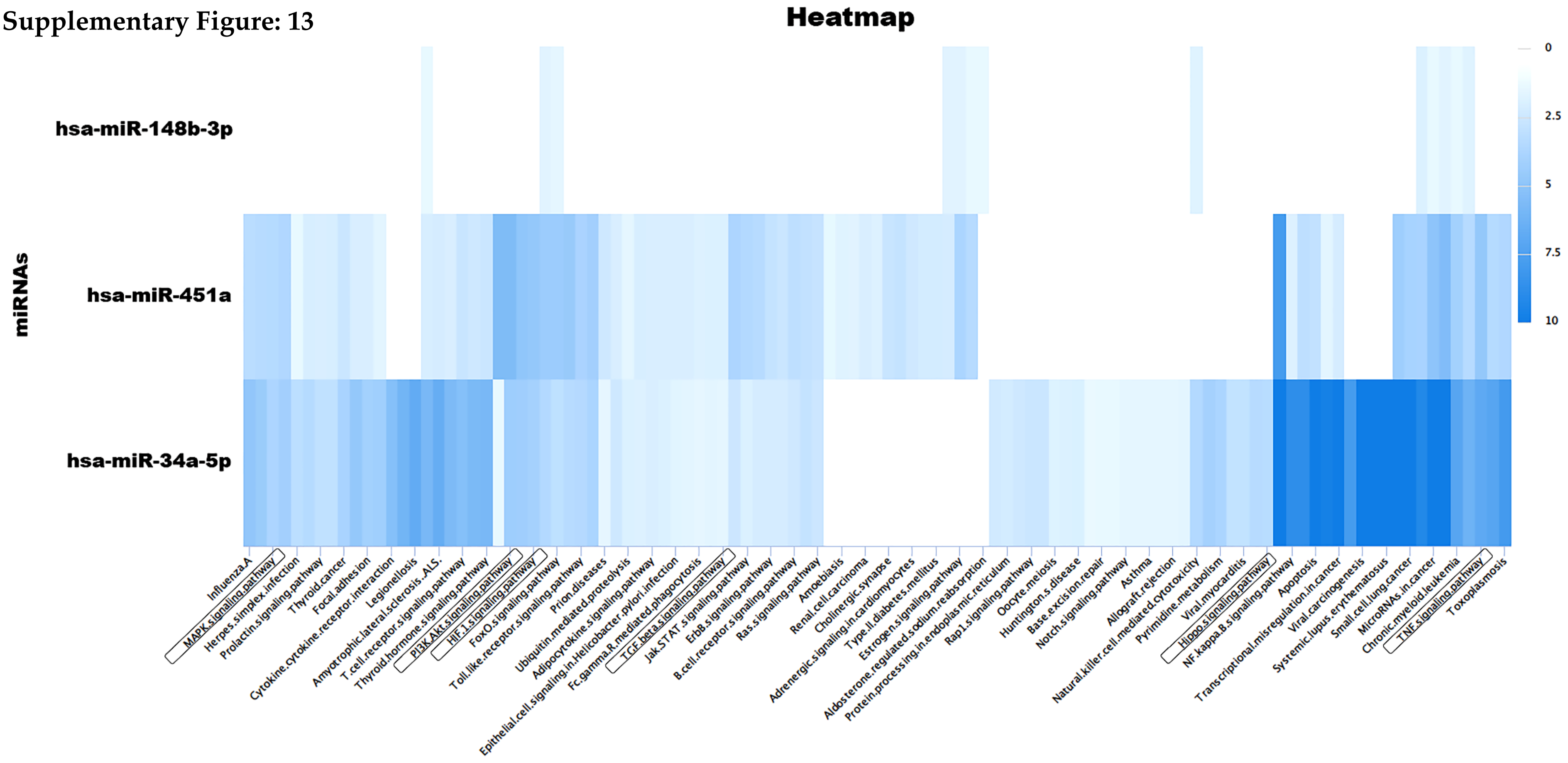

Supplementary Figure S13: KEGG analysis of shortlisted exosomal-miRNA analyzed by miRPathDB v2.0 revealed target signaling pathways (highlighted by rectangular box) such as MAPK signaling pathway, PI3K-Akt signaling pathway, HIF-1 signaling pathway, TGF- β signaling pathway, Hippo signaling pathway, TNF signaling pathway etc. involved in HSCs lineage.

Supplementary Figure: 14

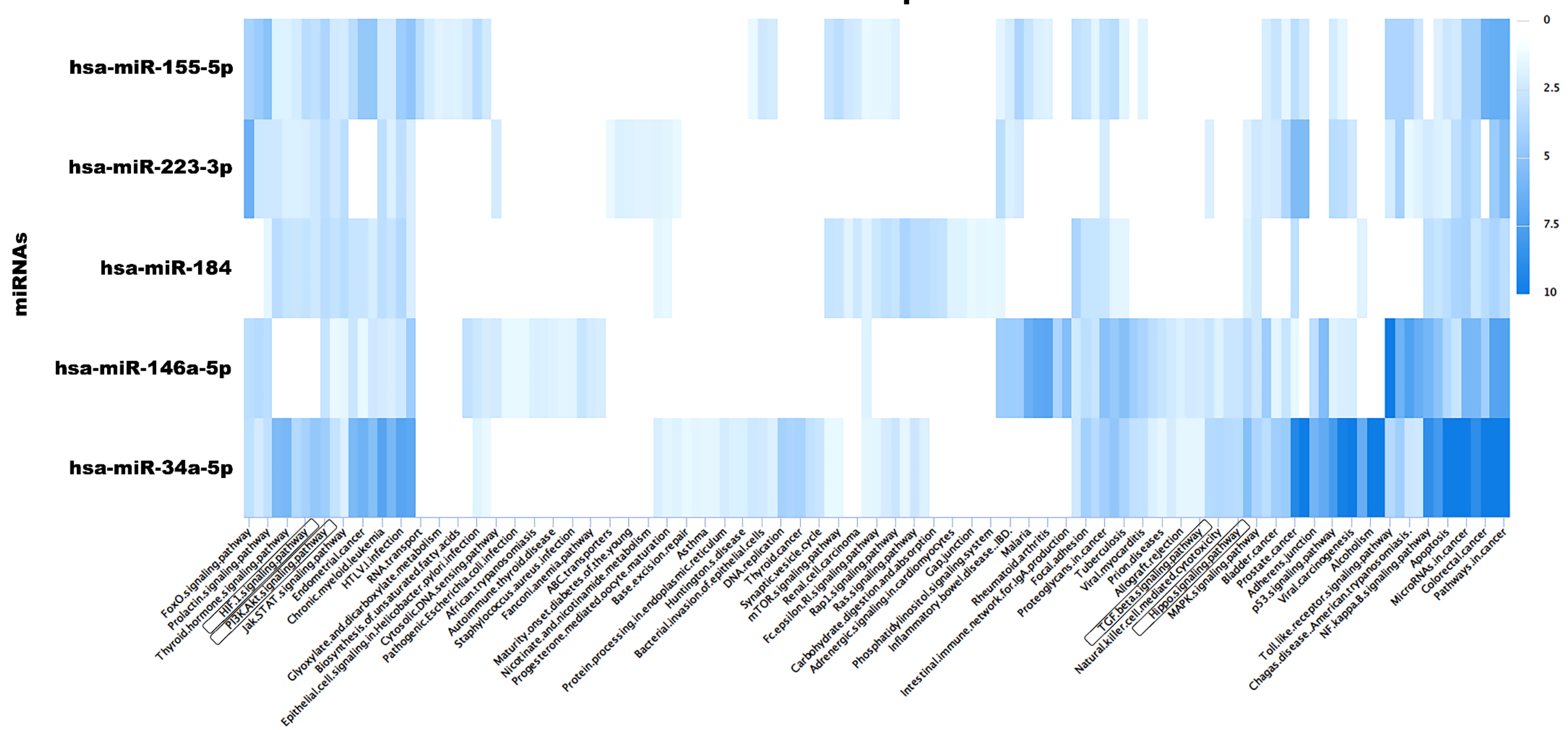

Supplementary Figure S14: KEGG analysis of shortlisted exosomal-miRNA analyzed by miRPathDB v2.0 revealed target signaling pathways (highlighted by rectangular box) such as HIF-1 signaling pathway, PI3K-Akt signaling pathway, TGF- β signaling pathway, Hippo signaling pathway etc. involved in HSCs differentiation.

Supplementary Figure: 15

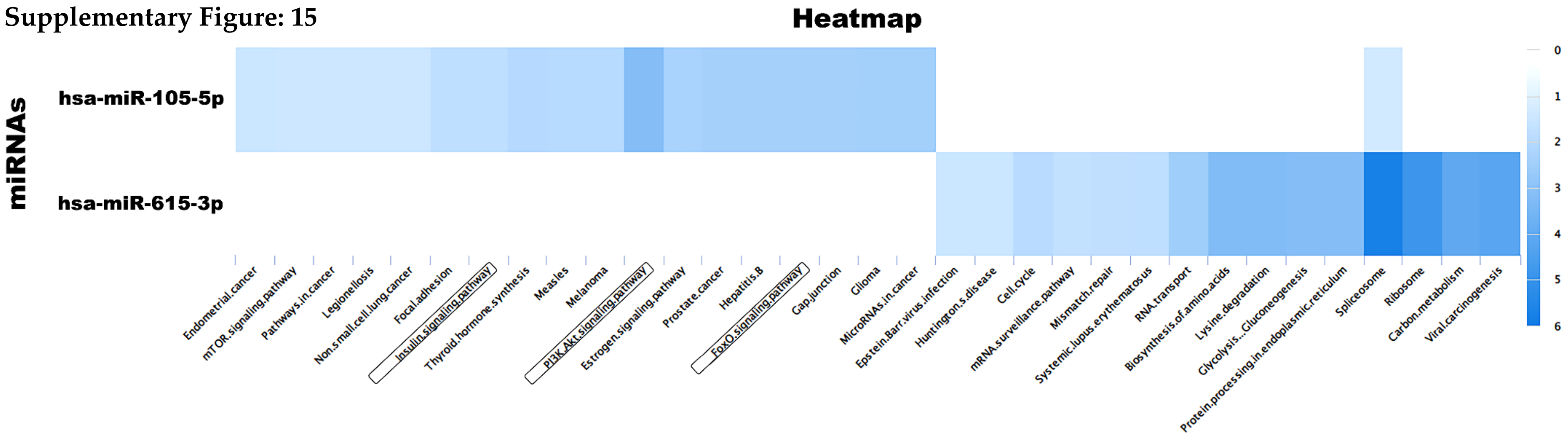

Supplementary Figure S15: KEGG analysis of shortlisted exosomal- miRNA analyzed by miRPathDB v2.0 revealed target signaling pathways (highlighted by rectangular box) such as Insulin signaling pathway, PI3K-Akt signaling pathway, FoxO signaling pathway etc. involved in regulation of HSCs differentiation.

Supplementary Figure: 16

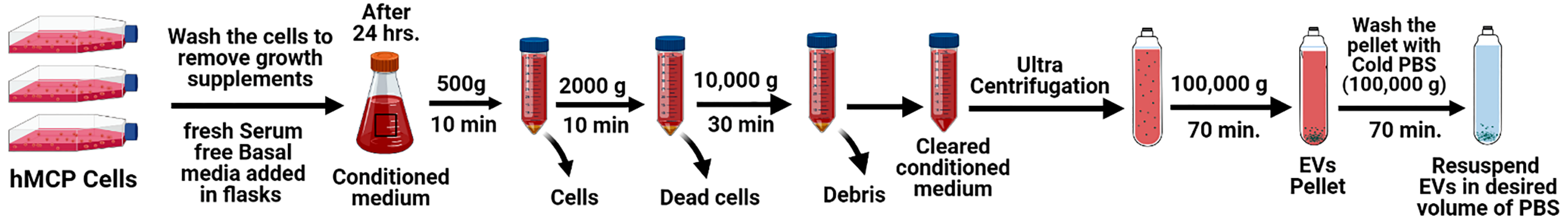

Supplementary Figure S16: A detailed schematic diagram for EVs isolation from hMCP conditioned media using Ultracentrifugation method. For expansion of hMCP cells were cultured at a density of 16-18 x 103 cells per cm^2 in T175 cm^2 flasks with 20ml of cell expansion media StemSpan™-XF with addition of StemSpan™ CD34+ Expansion Supplement and antibiotics. After 4 days of expansion the cells culture output was 52-55 x 103 cells per cm2. cells were washed with PBS two to three times followed by a final wash with supplement free Basal media for complete removal of cell expansion medium and antibiotics. Next 24 hours these hMCPs (52 x 10^3 cells per cm^2) were incubated with supplement free Basal media. After 24 hours conditioned media were collected for further in vivo and in vitro applications. Cells incubated over 24hours without growth supplement showed 82.4% viability with the concentration of 42.8 x 10^3 cells per cm^2. Conditioned media collected from different flasks and passage 4 and 5 pooled together and centrifuged at 500g for 10min at 4^0C to remove the cells. Cell free conditioned media was frozen at -80^0C until further use. hMCP cells were routinely performed for mycoplasma contamination testing. All the cells used in this study were negative for mycoplasma contamination.

Before EVs isolation pooled frozen conditioned media were thaw at 4^0C overnight and centrifuge two time at 2000g for 10 min and 10,000g for 30 min to remove dead cells and debris respectively before performing Ultracentrifugation steps. Cleared conditioned media were transferred to Open-Top Polyclear centrifuge tubes (Seton Scientific, USA, Part no. 7052) and fixed in Ultracentrifuged rotor (Superspin 630) bucket (Cat no. 79365) and Ultracentrifuge at 4^0C, 100,000g for 70minutes with rotor adjusted K factor 257. Supernatant was discarded and EVs pellet were washed with sterile cold PBS by centrifugation at 4^0C, 100,000g for 70minutes. Final EV pellets were resuspended in desired volume of sterile PBS for immediate use or stored at -80^0C until further use. EVs were further analyzed by using flowcytometry to determine EV associated proteins. EV Particle size and number was determined by Nanosight Tracking Assay (LM10, Malvern Inst. Ltd., UK). EV concentration was also determined by total protein estimation using BCA method.

Supplementary Table S1: List of Primers

Gene	Forward primer (5’-3’)	Reverse primer (5’-3’)
Human CD33	TTCCTCCTGTGGGTCTTCAC	CTTTCCAGGAGATGGCTCAG
Human NRG-3	TGCCGAGACAAGGACCTTGCAT	TCACAACGGACTCCTTGGTAGC
Human Tnfrs-11a	GAAGGTTGGCTGGACAGGAGTT	CGCATCGGATTTCTCTGTCCCA
Human Beta Actin	TGGATCAGCAAGCAGGAGTATG	GCATTTGCGGTGGACGAT

Supplementary Table S2a: Presence of Exosomal Proteins in Total Identified Proteins:

Total Identified Proteins from hMCP Conditioned Media	Presence of Exosomal Protein in total identified proteins (0= Non-exosomal proteins, 1= Exosomal Proteins)
**Entrez Gene ID**	
280717	1
213	1
4627	0
2316	1
7018	0
7094	0
3936	0
4353	0
7086	0
87	1
7431	0
81	1
8826	0
9961	0
60	1
3309	0
2821	1
4478	0
7057	0
1938	1
2934	1
5315	0
301	1
79026	0
2023	1
2665	1
302	1
3312	0
7534	0
10487	1
7414	0
4001	0
7167	0
3939	0
3320	0
2923	0
4000	0
309	1
8349	0
2597	1
440689	0
3326	0
5223; 643576	0
3329	0
847	1

Total Identified Proteins	Exosomal Protein
1447	315

5034	0
5052	0
1213	1
5660	0
7415	0
1992	0
7529	0
70	1
3945	0
1915	1
1072	0
70; 554313; 8365; 8364; 121504; 83	0
7171	0
5725	0
5272	0
4860	0
226	1
4048	0
6772	0
2194	0
8351; 8355; 8357; 8358; 8350; 8353	0
4869	0
526	0
506	0
11315	0
7531	0
5184	0
822	0
4125	0
6647	0
1890; 9997	0
4360	0
7430	1
10130	0
3009	0
5836	0
7360	0
7532	0
7453	0
5688	0
10072	0
5230	0
5682	0
5687	0
328	1
7296	0
11344	0
203068	0
498	0
83706	1
8407	0

333932; 126961; 653604	0
5685	0
2512	0
5686	0
3178	0
5689	0
3609	0
3858	0
204	1
3006	1
5777	0
7184	0
1937	1
290	1
4190	0
808; 805; 801	0
3007	1
29108	0
6624	1
10963	0
6279	0
5339	0
3008	1
397	1
10376	0
1808	0
3308	0
51056	0
5284	0
5721	0
5341	0
2936	1
6280	0
3122	0
1642	1
7846	0
9446	1
5696	0
832	0
5216	0
3428	0
7408	0
7170	0
4691	0
10971	0
3020; 440926; 3021	0
5720	0
3148	0
6282	0
3098	0
5684	0

6233	0
10097	1
7295	0
3689	0
5962	0
2058	1
4628	0
4069	0
3684	0
3184	0
10383	0
826	0
5698	0
10961	1
1622	1
3074	1
2547	0
929	0
4191	0
5226	0
3146	0
5479	0
230	1
2203	1
6888	0
1192	0
47	1
9520	0
4830	0
81567	0
10492	0
7175	0
23344	1
523	0
5683	0
7520	0
142	0
55748	0
2896	1
5908	0
56886	0
23603	1
7329	0
829	1
2746	0
3190	0
2664	1
5037	0
5269	0
10096	0
3126	0

88	1
3191	0
8288	1
3192	0
9555	1
140885	0
26986	0
5898	0
54504	1
6036	0
5553	0
6421	0
7533	0
7037	0
11198	0
708	0
396	1
1511	1
51031	0
22827	0
4926	0
10627	0
4831; 654364	0
103910; 10627	0
7203	1
7001	0
5887	0
11164	0
6181	0
2799	1
10493	0
79180	1
4082	0
8125	1
3035	1
4637	0
9255	1
25796	0
3183	0
23071	1
162	1
5093	0
51466	0
100	1
11316	1
1975	1
3181	0
10007	1
3674	0
2271	1

55920	0
4670	0
6709	0
6711	0
2745	0
10606	0
9131	0
7416	0
3185	0
5690	0
27044	0
308	1
5692	0
2171	1
84823	0
10236	0
2184	1
6950	0
5036	0
6175	0
10935	0
8880	1
1509	1
6741	0
3251	0
10577	0
7332	0
3304; 3303	0
55968	0
7919	1
9948	0
6050	0
3799	0
9588	0
3417	0
5473	0
79017	1
23193	1
2937	1
7555	0
387	0
3115	0
25939	0
3068	0
11151	1
55276	0
5691	0
4841	0
3959	0
2539	1

7112	0
6634	0
760	0
1981	0
10575	1
5271	0
5510	0
1075	1
3958	0
5878	0
2950	1
22919	0
9559	0
8775	0
51411	0
23020	0
5245	0
11335; 90416	0
3849	0
1973	1
56005	0
10525	0
6628	0
6189	0
3105	0
10327	1
7178	0
8662	1
3848	0
8338	0
4052	0
9126	0
7155	0
23406	1
3482	0
5931	0
2805	1
7873	0
9967	0
3690	0
6500	0
55700	0
4013	0
567	0
143244	1
5868	0
5478	0
3916	0
10923	0
1457	1
823	1

8243	0
2026	1
51571	1
3956	0
6601	0
27101	1
214	1
3507	0
83442	0
10539	1
10576	1
6285	0
51292	1
3149	0
976	1
51631	0
2027	1
2885	1
4666	0
5273	0
2288	1
1520	0
22820	1
5589	0
516	0
4172	0
1660	1
5781	0
389898	0
1738	1
2876	1
2517	1
831	0
8635	0
3608	0
728689	1
8661	1
10989	0
5524	0
327	1
2317	1
3151	0
9782	0
4043	0
1991	1
5516	0
4076	0
10574	1
64167	0
8411	1

9208	0
163	1
440686	0
4678	0
10521	1
6917	0
10437	0
2760	1
7536	0
9793	0
821	1
6125	0
1778	0
6451	0
10095	0
6275	0
427	0
126133	1
11021	0
4175	0
830	0
10549	0
6202	0
11345	1
7317	0
51816	0
8836	1
2040	0
6426	0
8761	0
5265	0
989	1
5204	0
92241	0
4738	0
5886	0
6139	0
6897	0
11054	0
51752	1
3118	0
2962	1
7257	0
8815	0
310	1
3295	0
4735	1
3150	0
6251	0
10189	1
8721	1

160	1
54039	0
25824	0
23157	1
84064	1
11337	0
8570	0
4904	0
2935	0
56729	0
5518	0
5499	0
10409	0
6185	0
6386	0
566	0
5094	0
11331	0
11338	0
6130	0
908	1
3187	0
9939	0
1178	0
4736	0
10262	0
1314	1
5928	0
10059	1
8971	1
1508	1
5110	0
22929	0
2806	1
813	1
8804	1
1431	1
3514	0
6224	0
5464	0
10808	0
200081	0
1729	1
11140	0
9352	0
4050	0
9218	0
3313	0
311	1
2739	0
11051	0

4668	0
6632	0
7266	1
5356	0
767558; 136319	0
8209; 102724023	0
7411	0
64710	0
5699	0
3920	0
57092	0
7247	0
6137	0
56954	0
9097	0
3073	1
6902	0
6294	0
23244	0
3500	0
2162	1
5757	0
2633	1
10399	1
10938	1
8260	0
22948	1
27131	0
9111	0
10695	0
6418	0
5530	0
27316	0
1108	0
9987	0
22826	1
6120	0
10457	1
5048	0
55379	0
392	1
10726	0
5704	0
6223	0
400668	0
5330	0
353	1
10109	0
25932	0
2289	1
51602	0

5861	0
4046	0
1786	1
128866	0
6749	0
4288	0
10483	0
811	1
5881	0
10055	0
26156	0
4332	0
3615	0
5885	0
16	0
11325	0
10598	0
3502	0
6188	0
8417	0
6636	0
2107	1
1854	0
10982	0
6161	0
29766	0
23385	0
84817	0
1965	1
59345	1
9550	0
8789	0
55611	0
23646	0
7073	0
10954	0
2224	0
1933	1
8668	1
8667	1
5049	0
6222	0
220988	0
5954	0
2130	0
22872	0
1653	1
6037	0
1639	1
3538	0

2804	1
8665	1
23593	1
231	1
10915	0
191	0
23549	1
3837	0
26528	0
5860	0
8565	0
6102	0
3840	0
8498	0
6156	0
6431	0
9958	0
115207	0
6146	0
3123	0
51726	1
654346	0
529	0
6138	0
2495	0
3301	1
5591	0
5547	0
3157	0
10155	0
10212	0
3093	0
4144	0
6432	0
10093	0
8226	0
2771	1
51074	0
6208	0
5813	0
4924	0
6633	0
26135	0
6152	0
9938	0
2314	1
3119	0
4522	0
84932	0
372	1

26578	0
8669	1
1371	1
7174	0
84844	0
3336	0
10728	0
1399	1
10226	0
5708	0
6128	0
6635	0
27243	0
54440	0
3925	0
7114	0
682	1
23499	0
1476	1
9276	1
25801	1
5196	0
1994	1
7323	0
1743	1
6449	0
1088	0
2029	0
7072	0
3383	0
216	1
5912	0
54205	0
388	0
2990	0
7336	0
3385	0
8607	0
199	0
6187	0
10394	0
11171	0
6427	0
51382	0
3895	0
5935	0
6118	0
1968	1
2287	0
10197	0

2079	0
8241	0
6117	0
57175	1
9527	1
1340	0
9459	0
10135	0
5111	0
92675	0
3692	1
960	0
51596	1
9092	0
4835	0
5576	0
30001	1
5723	0
6184	0
3109	0
23234	1
51690	0
131076	0
10015	0
2773	1
3028	0
90952	1
6206	0
670	0
689	0
1655	1
6342	0
84836	0
10294	1
10291	0
9774	0
6191	0
9050	0
4035	0
5295	0
84717	0
6230	0
6403	0
7265	0
6277	0
2969	1
5869	0
27258	0
5705	0
7419	0

116496	1
3978	0
51719	1
10204	0
5531	0
10657	0
6727	0
30011	0
51138	1
134510	0
306	1
545	0
1459	1
51691	0
3949	0
2280	0
8402	0
22913	0
8942	0
350	1
9554	0
873; 54093	0
54749	0
4074	0
3676	0
3263	0
8878	0
1615	1
9689	0
10987	0
1665	1
2802	1
5236	0
6627	0
51060	0
4676	0
9045	0
25996	0
3065	1
29979	0
11252	0
55646	0
5202	0
11168	0
23075	0
51493	0
3043	1
58513	1
10094	0
2618	1
10146	1

1066	0
27306	0
3107	0
80150	0
10541	1
51154	0
9138	1
3113	0
8520	1
9943	0
5859	0
10694	0
23524	0
5702	0
1654	0
3921	0
5770	0
8467	0
9601	0
3315	0
2782	1
4706	0
23450	0
50848	1
29941	0
2992	0
1478	0
5701	0
5007	0
6734	0
6742	0
4282	0
10856	0
644	0
58478	0
1845	0
7345	0
10250	0
4849	0
8904	1
10053	1
4673	0
57095	0
8574	1
1429	1
6599	0
8766	0
1611	0
3501	0
10450	0

10380	0
5694	0
10209	1
1460	1
7177; 64499	0
25804	0
55911	0
3208	0
10054	0
81027	0
2752	1
1650	1
9141	0
495	0
6194	0
6286	0
6726; 653226	0
55707	0
7064	0
7319	0
152007	1
10480	1
133619	0
7165	0
10051	0
972	0
57486	0
6229	0
7812	1
8943	1
307	1
399687	0
27339	0
1656	1
10946	0
2734	1
7153	0
475	0
528	0
39	0
55313	0
4728	0
6122	0
3117	0
998	0
127281	1
6448	0
10652	0
29952	0

57819	0
58527	0
9798	0
23760	0
22818	1
58517	0
8530	0
1936	1
22934	0
6136	0
6201	0
3248	0
10326	0
945	0
6642	0
26136	0
3127	0
29086	0
4597	0
11313	0
10589	0
57727	0
30968	0
683	1
10907	0
9815	0
51765	0
11332	1
5695	0
6434	0
9114	0
7343	0
51667	0
9980	0
10849	0
9879	1
23211	0
10949	0
51172	0
10105	0
7076	0
6817	0
51291	0
6168	0
7913	0
3040; 3039	0
5901	0
10981	0
1036	0

515	0
6301	0
3852	0
51762	0
9040	0
51608	1
9931	0
9246	0
645	0
3163	0
57674	0
9749	0
23186	0
4702	0
10788	0
10659	0
79869	1
5069	0
7150	0
1646	0
22931	0
3843	0
29937	0
23111	0
8514	0
4340	0
55335	0
283742	0
9984	0
1892	1
27095	0
8078	0
1495	1
5715	0
51024	0
23114	0
1432	0
391059	0
8773	0
6039	0
146722	0
8539	1
10152	0
81873	0
5714	0
58155	0
11333	0
9402	1
9589	0
57446	0
57470	0

23033	0
4671	0
84955	0
10890	0
3735	0
23085	0
5336	0
5573	0
4482	0
1777	0
2620	0
3182	0
5203	0
51155	0
522	0
10432; 100526737	0
345	1
145165	0
6234	0
9444	0
93100	0
2879	1
23078	0
6646	0
1069	0
1191	1
8544	0
63929	0
79366	0
5303	0
10043	0
3145	0
3845	0
1739	1
1522	1
10592	0
4267	0
6728	0
1810	0
5903	0
7706	0
54939	0
11314	0
55660	0
10980	1
10254	0
4301	0
2617	1
8829	0

414061	1
3798	0
3612	0
4501	0
5437	0
374454	0
7979	0
30844	1
58472	0
3857	0
83592	1
84991	0
2195	1
5700	0
118611	0
4507	0
51143	0
3029	0
23	0
87769	1
51206	1
9590	1
10075	0
4134	0
645683	0
125950	0
8895	1
7264	0
5965	0
1410	1
4925	0
10540	1
27161	0
8894	1
5306	0
11052	0
51593	0
9861	0
5250	0
79165	0
10801	0
669	0
9696	1
175	0
2339	1
3054	1
6829	0
58477	0
56301	0

654483; 552900; 107984053	0
3162	0
25777	0
3512	0
8832	0
4801	0
7454	0
145553	0
6217	0
116984	0
10992	0
9524	0
8455	0
2634	0
9343	1
57523	0
6166	0
5870	0
3430	0
408	0
64077	0
341676	0
51428	0
23557	0
27074	0
23558	0
5634	0
51506	0
8655	0
2091	1
91298	0
28988	1
100302736; 51014	0
10960	0
8436	0
6625	0
293	0
8106	0
2729	0
6207	0
26472	0
51110	0
126208	0
388946	0
10951	1
6160	0
1475	1
10910	0
3281	0
23526	0
91754	0

115361	0
51237	0
283417	0
8603	0
23042; 102724985	0
1486	0
6141	0
10666	0
29082	0
3159	0
3431	0
54832	0
54414	0
9482	0
8548	0
84687	0
6133	0
3796	0
79002	0
9320	0
1047	0
1445	1
25909	0
5201	0
23028	0
978	0
1153	0
10061	0
4905	0
9531	0
4033	0
51560	0
969	0
10092	0
3646	1
9818	0
132	1
10542	0
81609	0
5092	0
54453	0
60491	0
51386	1
10476	0
64802	0
10855	0
1952	0
10131	0
6249	0
2260	1

261726	0
4725	0
1803	0
9377	0
6205	0
112942	0
1359	0
7994	0
53938	0
83473	0
79709	1
3687	0
8905	0
8450	1
2521	0
6169	0
1891	1
115098	0
26092	0
6745	0
6155	0
3094	1
5657	0
387921	0
833	1
9360	0
55183	0
4124	0
9667	0
54732	0
7385	0
6810	0
56267	1
9669	1
3635	0
56893	0
26519	0
5874	0
10228	0
6135	0
6228	0
51115	0
7518	0
201294	0
1781	0
54472	0
128240	0
1788	1
58511	0
55004	0
9219	0

4809	0
159	0
10211	1
274	0
9221	0
23118	0
5917	0
5442	0
23307	1
2533	1
4034	0
84661	0
317749	0
58490	0
11235	0
6907	0
7879	0
210	1
134492	0
5621	0
83483	0
57187	0
8737	0
2286	0
50861	0
2823	1
131474	0
7965	1
1536	1
55623	0
79751	0
7450	0
2207	1
57476	0
54	0
8545	0
6051	0
7163	0
51389	0
958	0
54976	0
4218	0
2060	1
23052	1
11157	0
51015	0
65109	0
6923	0
5713	0
8664	1

3135	0
2098	0
5795	0
29085	0
24139	1
2037	1
10920	1
9130	0
4688	0
6176	0
23102	0
374659	0
9181	0
1471	1
92140	0
23475	0
4174	0
1635	0
1652	1
5707	0
54951	0
3059	0
4716	0
4067	0
29925	1
3927	0
441549	0
23673	0
4436	0
80273	0
64422	0
51347	0
9775	1
8615	0
8682	0
4599	0
128	1
10594	0
10528	0
6693	0
3161	0
10484	0
64328	0
23196	0
81631	0
642	0
83699	0
51260	0
4179	0
9733	0

152137	1
5706	0
103	1
23517	0
51747	0
55844	0
22978	0
79577	0
9584	0
962	0
3092	1
10434	0
4660	0
6748	0
51071	1
81611	1
26999	1
6708	0
5718	0
2219	1
2182	1
7791	0
6717	0
411	0
55727	0
55737	0
84154	0
9146	1
84898	0
55665	0
292	0
5130	0
5604	0
4065; 100526664; 9936	0
5198	0
55651	0
55577	0
10653	0
4542	0
8666	1
164	1
4116	0
2814	1
9368	0
57103	0
3416	0
23451	0
961	0
8301	0
9647	0
3032	1

6921	0
6193	0
573	0
11165	0
6637	0
83641	0
5552	0
125061	0
9525	0
5019	0
55573	0
5331	0
509	0
91526	1
79031	0
4204	0
23163	1
5434	0
9129	0
150684	0
1785	1
54927	0
2801	1
26058	0

Supplementary Table S2b: Functions of Total Identified Proteins from hMCP conditioned media:

Entrez Gene ID	Involved in Biological Process	Cellular Component	Molecular Function
280717	cell communication;regulation of biological process;response to stimulus;transport	extracellular	DNA binding;metal ion binding
213	cell communication;cell organization and biogenesis;metabolic process;regulation of biological process;response to stimulus;transpor	cytoplasm;endoplasmic reticulum;extracellular;Golgi;nucleus;organelle lumen	antioxidant activity;catalytic activity;DNA binding;metal ion binding;protein binding
4627	cell differentiation;cell organization and biogenesis;cellular component movement;metabolic process;regulation of biological process;	cytoplasm;cytoskeleton;cytosol;extracellular;membrane;nucleus	catalytic activity;motor activity;nucleotide binding;protein binding;RNA binding;transporter activity
2316	cell organization and biogenesis;cellular component movement;metabolic process;regulation of biological process;response to stimul	cytoplasm;cytoskeleton;cytosol;extracellular;membrane;nucleus	protein binding;RNA binding;signal transducer activity
7018	cell organization and biogenesis;cellular homeostasis;metabolic process;regulation of biological process;response to stimulus;transp	cell surface;endosome;extracellular;membrane;organelle lumen	enzyme regulator activity;metal ion binding;protein binding;transporter activity
7094	cell organization and biogenesis;cellular component movement;regulation of biological process;response to stimulus;transport	cell surface;cytoplasm;cytoskeleton;cytosol;extracellular;membrane	protein binding;structural molecule activity
3936	cell organization and biogenesis;cellular component movement;regulation of biological process;response to stimulus	cytoplasm;cytoskeleton;cytosol;membrane	metal ion binding;protein binding
4353	cell organization and biogenesis;defense response;metabolic process;regulation of biological process;response to stimulus;transport	extracellular;mitochondrion;nucleus;organelle lumen;vacuole	antioxidant activity;catalytic activity;metal ion binding
7086	metabolic process;regulation of biological process	cytosol	catalytic activity;metal ion binding;protein binding
87	cell differentiation;cell organization and biogenesis;regulation of biological process;transport	cytoplasm;cytoskeleton;cytosol;extracellular;membrane;organelle lumen	metal ion binding;protein binding;RNA binding
7431	cell differentiation;cell organization and biogenesis;cellular component movement;metabolic process;regulation of biological process;	cytoplasm;cytoskeleton;cytosol;membrane	protein binding;RNA binding;structural molecule activity
81	cell organization and biogenesis;cellular component movement;regulation of biological process;response to stimulus;transport	cytoplasm;cytoskeleton;cytosol;extracellular;nucleus;organelle lumen	DNA binding;metal ion binding;protein binding;RNA binding
8826	cell growth;regulation of biological process;response to stimulus;transport	cytoplasm;cytoskeleton;cytosol;membrane;nucleus	enzyme regulator activity;metal ion binding;protein binding
9961	regulation of biological process;response to stimulus;transport	cytoplasm;cytoskeleton;cytosol;extracellular;membrane;nucleus;organelle lumen	protein binding
60	cell organization and biogenesis;cellular component movement;metabolic process;regulation of biological process;response to stimul	cytoplasm;cytoskeleton;cytosol;membrane;nucleus	DNA binding;nucleotide binding;protein binding;structural molecule activity
3309	cell communication;cell death;cell differentiation;cell organization and biogenesis;metabolic process;regulation of biological process;r	cell surface;cytoplasm;endoplasmic reticulum;membrane;mitochondrion;nucleus;organelle lumen	catalytic activity;metal ion binding;nucleotide binding;protein binding
2821	metabolic process;regulation of biological process;response to stimulus;transport	cytoplasm;cytosol;extracellular;membrane;organelle lumen	catalytic activity;protein binding
4478	cell differentiation;cell organization and biogenesis;cell proliferation;cellular component movement;regulation of biological process;res	cell surface;cytoplasm;cytoskeleton;cytosol;membrane;nucleus	protein binding;RNA binding;structural molecule activity
7057	cell organization and biogenesis;cellular component movement;defense response;metabolic process;regulation of biological process;	cell surface;cytoplasm;endoplasmic reticulum;extracellular;organelle lumen	metal ion binding;protein binding
1938	cell differentiation;cell proliferation;metabolic process;regulation of biological process;response to stimulus;transport	cytoplasm;cytosol;extracellular;membrane;nucleus;organelle lumen;ribosome	catalytic activity;nucleotide binding;protein binding;RNA binding
2934	cell death;cell organization and biogenesis;metabolic process;regulation of biological process;response to stimulus;transport	cytoplasm;cytoskeleton;cytosol;extracellular;membrane;nucleus;organelle lumen	metal ion binding;protein binding
5315	cell death;cell organization and biogenesis;metabolic process;response to stimulus;transport	cytoplasm;cytosol;extracellular;mitochondrion;nucleus;organelle lumen	catalytic activity;metal ion binding;nucleotide binding;protein binding;RNA binding
301	cell differentiation;cell organization and biogenesis;cellular component movement;defense response;metabolic process;regulation of	cell surface;cytoplasm;cytoskeleton;cytosol;endosome;extracellular;membrane;nucleus;vacuole	catalytic activity;DNA binding;enzyme regulator activity;metal ion binding;protein binding;RNA bind
79026	cell organization and biogenesis;regulation of biological process	cytoplasm;cytoskeleton;cytosol;membrane;nucleus	protein binding;RNA binding;structural molecule activity
2023	metabolic process;regulation of biological process;response to stimulus	cell surface;cytoplasm;cytosol;membrane;nucleus	catalytic activity;DNA binding;metal ion binding;protein binding;RNA binding
2665	metabolic process;regulation of biological process;response to stimulus;transport	cytoplasm;cytosol;extracellular;membrane;organelle lumen	catalytic activity;enzyme regulator activity;protein binding;RNA binding
302	cell organization and biogenesis;metabolic process;regulation of biological process;response to stimulus;transport	cell surface;cytoplasm;cytosol;endoplasmic reticulum;endosome;extracellular;membrane;nucleus;organelle lum	enzyme regulator activity;metal ion binding;protein binding;RNA binding
3312	cell communication;cell organization and biogenesis;metabolic process;regulation of biological process;response to stimulus;transpor	cytoplasm;cytosol;endosome;extracellular;membrane;nucleus;organelle lumen;spliceosomal complex	catalytic activity;nucleotide binding;protein binding;RNA binding
7534	cell organization and biogenesis;metabolic process;regulation of biological process;response to stimulus;transport	cytoplasm;cytosol;membrane;mitochondrion;nucleus	catalytic activity;protein binding;RNA binding
10487	cell organization and biogenesis;cellular component movement;regulation of biological process;response to stimulus;transport	cytoplasm;cytoskeleton;extracellular;membrane;organelle lumen	protein binding
7414	cell growth;cell organization and biogenesis;cellular component movement;regulation of biological process;transport	cytoplasm;cytoskeleton;cytosol;extracellular;membrane;organelle lumen	protein binding;structural molecule activity
4001	regulation of biological process;response to stimulus	membrane;nucleus	catalytic activity;motor activity;protein binding;structural molecule activity
7167	development;metabolic process	cytosol;nucleus	catalytic activity;protein binding
3939	metabolic process;regulation of biological process;response to stimulus	cytoplasm;cytosol;membrane;nucleus	catalytic activity;nucleotide binding;protein binding
3320	cell organization and biogenesis;metabolic process;regulation of biological process;response to stimulus;transport	cytoplasm;cytosol;extracellular;membrane;nucleus;organelle lumen	catalytic activity;enzyme regulator activity;nucleotide binding;protein binding;RNA binding
2923	cellular homeostasis;metabolic process;regulation of biological process;response to stimulus;transport	cell surface;endoplasmic reticulum;membrane;nucleus;organelle lumen	catalytic activity;protein binding;RNA binding
4000	cell organization and biogenesis;regulation of biological process;response to stimulus;transport	cytosol;membrane;nucleus	protein binding;structural molecule activity
309	cell organization and biogenesis;cellular homeostasis;regulation of biological process;response to stimulus;transport	cytoplasm;membrane;mitochondrion	metal ion binding;nucleotide binding;protein binding;transporter activity
8349	cell organization and biogenesis;defense response;response to stimulus	chromosome;cytosol;nucleus	DNA binding;protein binding
2597	cell death;cell organization and biogenesis;defense response;metabolic process;regulation of biological process;response to stimulus	cytoplasm;cytoskeleton;cytosol;membrane;nucleus	catalytic activity;enzyme regulator activity;nucleotide binding;protein binding
440689	cell organization and biogenesis	chromosome;cytosol;nucleus	DNA binding;protein binding
3326	cell organization and biogenesis;metabolic process;regulation of biological process;response to stimulus;transport	cell surface;cytoplasm;cytosol;extracellular;membrane;mitochondrion;nucleus;organelle lumen	enzyme regulator activity;nucleotide binding;protein binding;RNA binding
5223; 643576	metabolic process;regulation of biological process;transport	cytoplasm;cytosol;extracellular;membrane;organelle lumen	catalytic activity;protein binding
3329	cell organization and biogenesis;cell proliferation;metabolic process;regulation of biological process;response to stimulus;transport	cell surface;cytoplasm;cytosol;endosome;membrane;mitochondrion;organelle lumen	catalytic activity;DNA binding;nucleotide binding;protein binding;RNA binding
847	cell differentiation;cell organization and biogenesis;metabolic process;regulation of biological process;response to stimulus;transport	cytosol;endoplasmic reticulum;extracellular;Golgi;membrane;mitochondrion;organelle lumen;vacuole	antioxidant activity;catalytic activity;metal ion binding;nucleotide binding;protein binding
5034	cell organization and biogenesis;cellular homeostasis;metabolic process;regulation of biological process;response to stimulus	endoplasmic reticulum;extracellular;membrane;organelle lumen	catalytic activity;metal ion binding;protein binding;RNA binding
5052	cell proliferation;cellular homeostasis;defense response;metabolic process;regulation of biological process;response to stimulus	cytoplasm;cytosol;mitochondrion;nucleus	antioxidant activity;catalytic activity;protein binding;RNA binding
1213	cell differentiation;cell division;cell organization and biogenesis;cellular component movement;metabolic process;regulation of biologi	cytoplasm;cytoskeleton;cytosol;endosome;membrane;vacuole	protein binding;RNA binding;structural molecule activity
5660	cell differentiation;metabolic process;regulation of biological process;response to stimulus;transport	extracellular;membrane;mitochondrion;organelle lumen;vacuole	catalytic activity;enzyme regulator activity;protein binding
7415	cell communication;cell organization and biogenesis;metabolic process;regulation of biological process;response to stimulus;transpor	cytoplasm;cytosol;endoplasmic reticulum;extracellular;membrane;nucleus;organelle lumen;proteasome	catalytic activity;enzyme regulator activity;nucleotide binding;protein binding;RNA binding
1992	regulation of biological process;transport	cytoplasm;extracellular;membrane;organelle lumen	enzyme regulator activity
7529	cell organization and biogenesis;metabolic process;regulation of biological process;response to stimulus;transport	cytoplasm;cytosol;membrane;mitochondrion;nucleus	protein binding
70	cell death;cell organization and biogenesis;cellular component movement;regulation of biological process;response to stimulus	cytoplasm;cytoskeleton;cytosol;membrane	catalytic activity;nucleotide binding;protein binding
3945	metabolic process	cytoplasm;cytosol;membrane;mitochondrion	catalytic activity;nucleotide binding;protein binding
1915	metabolic process;regulation of biological process;response to stimulus;transport	cytoplasm;cytoskeleton;cytosol;extracellular;membrane;nucleus;organelle lumen	catalytic activity;nucleotide binding;protein binding;RNA binding
1072	cell organization and biogenesis;cellular component movement;metabolic process;regulation of biological process;response to stimul	cytoplasm;cytoskeleton;cytosol;membrane;nucleus	protein binding
3; 8365; 8364; 12	cell organization and biogenesis;metabolic process;regulation of biological process;response to stimulus	chromosome;extracellular;membrane;nucleus	DNA binding;protein binding;RNA binding
7171	cell differentiation;cell organization and biogenesis;cellular component movement	cytoplasm;cytoskeleton;cytosol;membrane	metal ion binding;protein binding;structural molecule activity
5725	metabolic process;regulation of biological process;response to stimulus	membrane;nucleus	protein binding;RNA binding
5272	cell death;regulation of biological process;response to stimulus	cytoplasm;cytosol;membrane;nucleus	enzyme regulator activity;protein binding
4860	metabolic process;regulation of biological process;response to stimulus;transport	cytoplasm;cytoskeleton;cytosol;extracellular;nucleus;organelle lumen	catalytic activity
226	cell organization and biogenesis;cellular homeostasis;metabolic process;regulation of biological process;transport	cytoplasm;cytoskeleton;cytosol;extracellular;membrane;nucleus;organelle lumen	catalytic activity;protein binding;RNA binding
4048	metabolic process;transport	cytoplasm;cytosol;extracellular;nucleus	catalytic activity;metal ion binding;protein binding;RNA binding
6772	cell death;cell differentiation;cell proliferation;cellular component movement;defense response;metabolic process;regulation of biologi	cytoplasm;cytosol;nucleus	DNA binding;protein binding;signal transducer activity
2194	cell differentiation;metabolic process;regulation of biological process;response to stimulus	cytoplasm;cytosol;Golgi;membrane;mitochondrion	catalytic activity;nucleotide binding;protein binding;RNA binding
5; 8357; 8358; 83	cell organization and biogenesis;coagulation;metabolic process;regulation of biological process;response to stimulus	chromosome;extracellular;membrane;nucleus	catalytic activity;DNA binding;protein binding
4869	cell growth;cell organization and biogenesis;cellular homeostasis;metabolic process;regulation of biological process;response to stim	cytoplasm;cytoskeleton;cytosol;membrane;nucleus	DNA binding;enzyme regulator activity;protein binding;RNA binding
526	cellular homeostasis;metabolic process;regulation of biological process;response to stimulus;transport	cytoplasm;cytosol;membrane	catalytic activity;nucleotide binding;protein binding;transporter activity
506	cell differentiation;cell organization and biogenesis;cellular homeostasis;metabolic process;regulation of biological process;transport	cell surface;membrane;mitochondrion;nucleus;organelle lumen	catalytic activity;nucleotide binding;protein binding;transporter activity
11315	cell communication;cell organization and biogenesis;defense response;metabolic process;regulation of biological process;response t	chromosome;cytoplasm;cytosol;endoplasmic reticulum;membrane;mitochondrion;nucleus;organelle lumen	antioxidant activity;catalytic activity;DNA binding;enzyme regulator activity;metal ion binding;protei
7531	cell organization and biogenesis;cellular component movement;cellular homeostasis;metabolic process;regulation of biological proce	cytoplasm;cytosol;membrane;mitochondrion;nucleus	protein binding;RNA binding
5184	metabolic process		catalytic activity;metal ion binding;protein binding
822	cell organization and biogenesis;regulation of biological process	cytoplasm;extracellular;nucleus	protein binding
4125	metabolic process;transport	extracellular;organelle lumen;vacuole	catalytic activity;metal ion binding
6647	cell organization and biogenesis;cellular component movement;cellular homeostasis;metabolic process;regulation of biological proce	cytoplasm;cytosol;extracellular;membrane;mitochondrion;nucleus;organelle lumen;vacuole	antioxidant activity;catalytic activity;metal ion binding;protein binding
1890; 9997	cell differentiation;cell organization and biogenesis;cellular homeostasis;development;metabolic process;regulation of biological proc	cytosol;membrane;mitochondrion;organelle lumen	catalytic activity;metal ion binding;protein binding
4360	regulation of biological process;response to stimulus;transport	cell surface;endosome;membrane	protein binding;receptor activity;signal transducer activity
7430	cell differentiation;cell organization and biogenesis;cellular component movement;metabolic process;regulation of biological process;	cytoplasm;cytoskeleton;cytosol;endosome;membrane	protein binding;RNA binding
10130	cellular homeostasis;metabolic process;regulation of biological process;response to stimulus;transport	cytosol;endoplasmic reticulum;membrane;organelle lumen	catalytic activity;protein binding
3009	cell organization and biogenesis;regulation of biological process	chromosome;nucleus	DNA binding;protein binding;RNA binding
5836	cell death;metabolic process;transport	cytoplasm;cytosol;extracellular;organelle lumen	catalytic activity;nucleotide binding;protein binding
7360	metabolic process	cytoplasm;cytosol;nucleus	catalytic activity;metal ion binding;nucleotide binding;protein binding
7532	cell organization and biogenesis;regulation of biological process;response to stimulus;transport	cytoplasm;cytosol;membrane;mitochondrion	enzyme regulator activity;protein binding;RNA binding
7453	metabolic process;regulation of biological process	cytoplasm;cytosol;nucleus	catalytic activity;enzyme regulator activity;nucleotide binding;protein binding
5688	metabolic process;regulation of biological process;response to stimulus;transport	cytoplasm;cytosol;nucleus;proteasome	catalytic activity;nucleotide binding;protein binding
10072	metabolic process	cytoplasm;cytosol;membrane	catalytic activity;metal ion binding;protein binding
5230	cell differentiation;metabolic process;regulation of biological process;response to stimulus	cytoplasm;cytosol;membrane	catalytic activity;nucleotide binding;protein binding
5682	metabolic process;regulation of biological process;response to stimulus;transport	cytoplasm;cytosol;nucleus;proteasome	catalytic activity;protein binding;RNA binding
5687	metabolic process;regulation of biological process;response to stimulus;transport	cytoplasm;cytosol;nucleus;proteasome	catalytic activity;nucleotide binding;protein binding;RNA binding
328	cell organization and biogenesis;cellular homeostasis;metabolic process;regulation of biological process;response to stimulus	cytoplasm;endoplasmic reticulum;mitochondrion;nucleus;ribosome	catalytic activity;DNA binding;metal ion binding;protein binding;RNA binding
7296	cell proliferation;cellular homeostasis;metabolic process;regulation of biological process;response to stimulus;transport	cytoplasm;cytosol;mitochondrion;nucleus	antioxidant activity;catalytic activity;nucleotide binding;protein binding
11344	cell organization and biogenesis;regulation of biological process;response to stimulus	cytoplasm;cytoskeleton	nucleotide binding;protein binding;RNA binding
203068	cell division;cell organization and biogenesis;cellular component movement;defense response;regulation of biological process;respon	cytoplasm;cytoskeleton;extracellular;membrane;nucleus;organelle lumen	catalytic activity;nucleotide binding;protein binding;structural molecule activity
498	cell organization and biogenesis;metabolic process;regulation of biological process;transport	membrane;mitochondrion;organelle lumen	catalytic activity;nucleotide binding;protein binding;RNA binding;transporter activity
83706	cell organization and biogenesis;regulation of biological process;response to stimulus;transport	extracellular;membrane;organelle lumen	protein binding
8407	cell differentiation;transport	cytosol;extracellular	protein binding
932; 126961; 653	cell organization and biogenesis;coagulation;metabolic process;regulation of biological process;response to stimulus	chromosome;extracellular;nucleus	DNA binding;protein binding
5685	metabolic process;regulation of biological process;response to stimulus;transport	cytoplasm;cytosol;nucleus;proteasome	catalytic activity;protein binding
2512	cellular homeostasis;transport	cytosol;extracellular;membrane;organelle lumen;vacuole	metal ion binding;protein binding
5686	metabolic process;regulation of biological process;response to stimulus;transport	cytoplasm;cytosol;extracellular;nucleus;organelle lumen;proteasome	catalytic activity;nucleotide binding;protein binding

3178	cell communication;metabolic process;regulation of biological process;response to stimulus;transport	cytoplasm;membrane;nucleus;spliceosomal complex	DNA binding;protein binding;RNA binding
5689	cell organization and biogenesis;metabolic process;regulation of biological process;response to stimulus;transport	cytoplasm;cytosol;extracellular;nucleus;organelle lumen;proteasome	catalytic activity;protein binding
3609	defense response;metabolic process;regulation of biological process;response to stimulus	cytoplasm;extracellular;membrane;mitochondrion;nucleus	DNA binding;protein binding;RNA binding
3858	cell death;cell differentiation;metabolic process	cytoplasm;cytoskeleton;cytosol;membrane;nucleus	structural molecule activity
204	metabolic process	membrane;mitochondrion	catalytic activity;nucleotide binding
3006	cell organization and biogenesis;metabolic process;regulation of biological process	chromosome;nucleus	DNA binding;protein binding;RNA binding
5777	cell death;cell differentiation;cell proliferation;cellular component movement;defense response;metabolic process;regulation of biolog	cytoplasm;cytosol;extracellular;membrane;nucleus;organelle lumen	catalytic activity;protein binding;receptor activity;signal transducer activity
7184	cell organization and biogenesis;metabolic process;regulation of biological process;response to stimulus;transport	cytosol;endoplasmic reticulum;extracellular;membrane;nucleus;organelle lumen	metal ion binding;nucleotide binding;protein binding;RNA binding
1937	metabolic process;response to stimulus	cytoplasm;cytosol;endoplasmic reticulum;membrane;nucleus	catalytic activity;protein binding;RNA binding
290	cell communication;cell differentiation;development;metabolic process;regulation of biological process;response to stimulus;transport	membrane;nucleus	catalytic activity;metal ion binding;receptor activity
4190	metabolic process	cytoplasm;cytosol;mitochondrion	catalytic activity;nucleotide binding;protein binding
808; 805; 801	metabolic process;regulation of biological process;response to stimulus;transport	cytoplasm;cytoskeleton;cytosol;extracellular;membrane	catalytic activity;enzyme regulator activity;metal ion binding;protein binding;transporter activity
3007	cell organization and biogenesis;metabolic process;regulation of biological process	chromosome;nucleus	DNA binding;RNA binding
29108	cell death;cell organization and biogenesis;defense response;metabolic process;regulation of biological process;response to stimulus	cytoplasm;cytosol;endoplasmic reticulum;extracellular;mitochondrion;nucleus;organelle lumen	catalytic activity;enzyme regulator activity;protein binding
6624	cell organization and biogenesis;cell proliferation;cellular component movement;regulation of biological process	cytoplasm;cytoskeleton;cytosol	protein binding;RNA binding
10963	response to stimulus	cytoplasm;cytosol;Golgi;nucleus	protein binding;RNA binding
6279	cell death;cellular component movement;defense response;metabolic process;regulation of biological process;response to stimulus;tr	cytoplasm;cytoskeleton;cytosol;extracellular;membrane;nucleus;organelle lumen	metal ion binding;protein binding
5339	cell organization and biogenesis	cytoplasm;cytoskeleton;cytosol;membrane	protein binding;RNA binding;structural molecule activity
3008	cell organization and biogenesis;metabolic process;regulation of biological process	chromosome;nucleus	DNA binding;metal ion binding;nucleotide binding;protein binding;RNA binding
397	cellular component movement;development;regulation of biological process;response to stimulus	cytoplasm;cytoskeleton;cytosol;membrane	catalytic activity;enzyme regulator activity;protein binding
10376	cell division;cell organization and biogenesis;response to stimulus;transport	cytoplasm;cytoskeleton;membrane	catalytic activity;nucleotide binding;protein binding;RNA binding;structural molecule activity
1808	cell differentiation;cell organization and biogenesis;cellular component movement;development;metabolic process;regulation of biolog	cytoplasm;cytoskeleton;cytosol;membrane	catalytic activity;protein binding
3308	cell organization and biogenesis;response to stimulus;transport	cytoplasm;cytosol	nucleotide binding;protein binding
51056	metabolic process	cytoplasm;cytosol;mitochondrion;nucleus	catalytic activity;metal ion binding
5284	cell organization and biogenesis;regulation of biological process;response to stimulus;transport	extracellular;membrane	receptor activity;signal transducer activity
5721	metabolic process;regulation of biological process;response to stimulus;transport	cytoplasm;cytosol;membrane;proteasome	enzyme regulator activity;protein binding
5341	cell differentiation;cell organization and biogenesis;metabolic process;regulation of biological process;response to stimulus;transport	cytoplasm;cytosol;extracellular;membrane	protein binding
2936	cellular homeostasis;metabolic process;regulation of biological process;response to stimulus	cytoplasm;cytosol;mitochondrion;organelle lumen	antioxidant activity;catalytic activity;nucleotide binding
6280	cell communication;cell death;cellular component movement;defense response;metabolic process;regulation of biological process;re	cytoplasm;cytoskeleton;cytosol;extracellular;membrane;nucleus;organelle lumen	antioxidant activity;catalytic activity;metal ion binding;protein binding;signal transducer activity
3122	cell organization and biogenesis;regulation of biological process;response to stimulus	cell surface;endoplasmic reticulum;endosome;Golgi;membrane;vacuole	protein binding;receptor activity;signal transducer activity
1642	cell organization and biogenesis;metabolic process;regulation of biological process;response to stimulus	cytoplasm;nucleus	DNA binding;protein binding
7846	cell division;cell organization and biogenesis;regulation of biological process;transport	cytoplasm;cytoskeleton;cytosol;endosome;membrane;nucleus	catalytic activity;nucleotide binding;protein binding;structural molecule activity
9446	cellular homeostasis;metabolic process;regulation of biological process;response to stimulus	cytoplasm;cytosol	antioxidant activity;catalytic activity;protein binding
5696	cell differentiation;metabolic process;regulation of biological process;response to stimulus;transport	cytoplasm;cytosol;nucleus;proteasome	catalytic activity;protein binding
832	cell organization and biogenesis;cellular component movement;coagulation;regulation of biological process;transport	cytoplasm;cytoskeleton;cytosol	protein binding
5216	cell organization and biogenesis;regulation of biological process;response to stimulus	cytoplasm;cytoskeleton;cytosol;membrane;nucleus	enzyme regulator activity;nucleotide binding;protein binding;RNA binding
3428	cell communication;cell death;cell differentiation;cell proliferation;defense response;metabolic process;regulation of biological process	cytoplasm;cytosol;membrane;nucleus	DNA binding;protein binding;RNA binding
7408	cell organization and biogenesis;cellular component movement;regulation of biological process	cytoplasm;cytoskeleton;cytosol;membrane	protein binding
7170	cell organization and biogenesis;cellular component movement	cytoplasm;cytoskeleton;cytosol	protein binding;structural molecule activity
4691	regulation of biological process;response to stimulus	cytoplasm;membrane;nucleus	DNA binding;protein binding;RNA binding
10971	cell organization and biogenesis;regulation of biological process;response to stimulus;transport	cytoplasm;cytosol;membrane;mitochondrion	protein binding
020; 440926; 302	cell differentiation;cell organization and biogenesis;cell proliferation;coagulation;metabolic process;regulation of biological process;re	chromosome;extracellular;nucleus	DNA binding;protein binding
5720	metabolic process;regulation of biological process;response to stimulus;transport	cytoplasm;cytosol;proteasome	enzyme regulator activity;protein binding
3148	cell organization and biogenesis;cellular component movement;defense response;metabolic process;regulation of biological process;	chromosome;cytoplasm;extracellular;nucleus	DNA binding;protein binding;RNA binding
6282	regulation of biological process;response to stimulus;transport	cytoplasm;extracellular;nucleus;organelle lumen	metal ion binding;protein binding
3098	cellular homeostasis;metabolic process;regulation of biological process	cytosol;membrane;mitochondrion	catalytic activity;nucleotide binding;protein binding
5684	metabolic process;regulation of biological process;response to stimulus;transport	cytoplasm;cytosol;nucleus;proteasome	catalytic activity;nucleotide binding;protein binding
6233	cell communication;cell organization and biogenesis;cellular homeostasis;defense response;metabolic process;regulation of biologica	cytoplasm;cytosol;membrane;nucleus;ribosome	metal ion binding;protein binding;RNA binding;structural molecule activity
10097	cell division;cell organization and biogenesis;cellular component movement;regulation of biological process;response to stimulus;tran	cytoplasm;cytoskeleton;cytosol;extracellular;membrane;organelle lumen	nucleotide binding;protein binding;structural molecule activity
7295	cell communication;cell proliferation;cellular component movement;cellular homeostasis;metabolic process;regulation of biological pr	cytoplasm;cytosol;extracellular;mitochondrion;nucleus	antioxidant activity;catalytic activity;protein binding;RNA binding
3689	cell communication;cell death;cell differentiation;cell organization and biogenesis;cellular component movement;defense response;m	cell surface;membrane	metal ion binding;protein binding;receptor activity;signal transducer activity
5962	cell organization and biogenesis;regulation of biological process;response to stimulus	cytoplasm;cytoskeleton;membrane	protein binding;RNA binding
2058	cell organization and biogenesis;metabolic process;regulation of biological process;response to stimulus	cytoplasm;cytosol;membrane	catalytic activity;metal ion binding;nucleotide binding;protein binding;RNA binding
4628	cell organization and biogenesis;cellular component movement;metabolic process;regulation of biological process;transport	cytoplasm;cytosol;nucleus	catalytic activity;motor activity;nucleotide binding;protein binding;transporter activity
4069	defense response;metabolic process;response to stimulus;transport	extracellular;organelle lumen	catalytic activity;protein binding
3684	cell differentiation;cell organization and biogenesis;cellular component movement;regulation of biological process;response to stimulu	cell surface;membrane	metal ion binding;protein binding
3184	metabolic process;regulation of biological process;response to stimulus	cytoplasm;cytosol;nucleus	DNA binding;protein binding;RNA binding
10383	cell organization and biogenesis;cellular component movement;defense response;regulation of biological process;response to stimulu	cytoplasm;cytoskeleton;cytosol;extracellular;nucleus;organelle lumen	catalytic activity;nucleotide binding;protein binding;RNA binding;structural molecule activity
826	cell death;cell organization and biogenesis;metabolic process;regulation of biological process	cytoplasm;cytosol;membrane	catalytic activity;metal ion binding;protein binding
5698	metabolic process;regulation of biological process;response to stimulus;transport	cytoplasm;cytosol;nucleus;proteasome	catalytic activity;protein binding
10961	metabolic process;regulation of biological process;transport	cell surface;endoplasmic reticulum;membrane;organelle lumen	catalytic activity;protein binding
1622	metabolic process;transport	endoplasmic reticulum;Golgi;organelle lumen	protein binding
3074	cell organization and biogenesis;cellular component movement;cellular homeostasis;metabolic process;regulation of biological proce	extracellular;membrane;organelle lumen;vacuole	catalytic activity;protein binding
2547	cell organization and biogenesis;defense response;metabolic process;regulation of biological process;response to stimulus;transport	chromosome;cytoplasm;cytosol;extracellular;membrane;nucleus;organelle lumen	catalytic activity;DNA binding;nucleotide binding;protein binding;RNA binding
929	cell death;defense response;regulation of biological process;response to stimulus;transport	cell surface;extracellular;Golgi;membrane	protein binding;receptor activity;signal transducer activity
4191	metabolic process	membrane;mitochondrion;nucleus;organelle lumen	catalytic activity;nucleotide binding;protein binding;RNA binding
5226	metabolic process	cytoplasm;cytosol;nucleus	catalytic activity
3146	cell differentiation;cell organization and biogenesis;cell proliferation;cellular component movement;cellular homeostasis;defense resp	cell surface;chromosome;cytoplasm;endosome;extracellular;membrane;nucleus;organelle lumen	catalytic activity;DNA binding;enzyme regulator activity;protein binding;RNA binding
5479	metabolic process;regulation of biological process	endoplasmic reticulum;membrane;nucleus;organelle lumen	catalytic activity;protein binding;RNA binding
230	cell differentiation;metabolic process;transport	cytoskeleton;cytosol;extracellular;mitochondrion;organelle lumen	catalytic activity;protein binding
2203	cell organization and biogenesis;metabolic process;regulation of biological process;response to stimulus	cytoplasm;cytosol;nucleus	catalytic activity;metal ion binding;nucleotide binding;protein binding
6888	metabolic process;regulation of biological process;response to stimulus	cytoplasm;cytosol;nucleus	catalytic activity;protein binding
1192	metabolic process;regulation of biological process;response to stimulus;transport	cytoplasm;membrane;mitochondrion;nucleus	catalytic activity;protein binding;transporter activity
47	metabolic process;regulation of biological process;transport	cytoplasm;cytosol;extracellular;membrane;organelle lumen	catalytic activity;metal ion binding;nucleotide binding;protein binding
9520	metabolic process;response to stimulus	cytoplasm;cytosol;nucleus	catalytic activity;metal ion binding
4830	cell differentiation;cellular homeostasis;metabolic process;regulation of biological process;response to stimulus;transport	cytoplasm;cytosol;membrane;mitochondrion;nucleus	catalytic activity;DNA binding;metal ion binding;nucleotide binding;protein binding;RNA binding
81567	cellular homeostasis;metabolic process;regulation of biological process;response to stimulus;transport	endoplasmic reticulum;extracellular;organelle lumen	catalytic activity;protein binding
10492	cell differentiation;metabolic process;regulation of biological process;response to stimulus	cytoplasm;endoplasmic reticulum;membrane;nucleus;spliceosomal complex	protein binding;RNA binding
7175	cell division;cell organization and biogenesis;metabolic process;regulation of biological process;response to stimulus;transport	chromosome;cytoplasm;cytoskeleton;membrane;nucleus	catalytic activity;motor activity;protein binding;RNA binding;structural molecule activity;transporter
23344	metabolic process;transport	endoplasmic reticulum;membrane	metal ion binding;protein binding
523	cellular homeostasis;metabolic process;regulation of biological process;response to stimulus;transport	cytosol;membrane;mitochondrion	catalytic activity;nucleotide binding;transporter activity
5683	metabolic process;regulation of biological process;response to stimulus;transport	cytoplasm;cytosol;extracellular;nucleus;organelle lumen;proteasome	catalytic activity;protein binding
7520	cell differentiation;cell organization and biogenesis;cell proliferation;defense response;metabolic process;regulation of biological proc	chromosome;cytoplasm;cytosol;extracellular;membrane;nucleus;organelle lumen	catalytic activity;DNA binding;enzyme regulator activity;nucleotide binding;protein binding;RNA bin
142	cell death;cell differentiation;cell organization and biogenesis;metabolic process;regulation of biological process;response to stimulus	membrane;mitochondrion;nucleus	catalytic activity;DNA binding;metal ion binding;nucleotide binding;protein binding;RNA binding
55748	metabolic process	cytoplasm;cytosol	catalytic activity;metal ion binding
2896	regulation of biological process;response to stimulus;transport	endoplasmic reticulum;endosome;extracellular;organelle lumen;vacuole	protein binding;RNA binding
5908	cell organization and biogenesis;cell proliferation;regulation of biological process;response to stimulus;transport	cytoplasm;cytosol;membrane	catalytic activity;nucleotide binding;protein binding
56886	metabolic process;regulation of biological process;response to stimulus	endoplasmic reticulum;organelle lumen	catalytic activity;protein binding
23603	cell organization and biogenesis;cellular component movement;regulation of biological process;response to stimulus;transport	cytoplasm;cytoskeleton;membrane	protein binding
7329	cell division;metabolic process;regulation of biological process;response to stimulus	cytoplasm;cytosol;nucleus	catalytic activity;nucleotide binding;protein binding;RNA binding
829	cell organization and biogenesis;cellular component movement;coagulation;defense response;regulation of biological process;respor	cytoplasm;cytoskeleton;cytosol;extracellular	protein binding
2746	cell organization and biogenesis;metabolic process;regulation of biological process	cytoplasm;mitochondrion;organelle lumen	catalytic activity;nucleotide binding;protein binding
3190	metabolic process;regulation of biological process;response to stimulus	cytoplasm;membrane;nucleus;spliceosomal complex	DNA binding;protein binding;RNA binding
2664	metabolic process;regulation of biological process;response to stimulus;transport	cytoplasm;cytosol;Golgi	catalytic activity;enzyme regulator activity;protein binding
5037	metabolic process;regulation of biological process;response to stimulus	cytoplasm;cytosol;nucleus	enzyme regulator activity;nucleotide binding;protein binding;RNA binding
5269	regulation of biological process;response to stimulus;transport	cytoplasm;cytosol;extracellular;membrane;nucleus	enzyme regulator activity;protein binding
10096	cell division;cell organization and biogenesis;cellular component movement;regulation of biological process;response to stimulus	cytoplasm;cytoskeleton;cytosol;membrane	nucleotide binding;protein binding;structural molecule activity
3126	regulation of biological process;response to stimulus	endoplasmic reticulum;endosome;Golgi;membrane;vacuole	
88	cell organization and biogenesis;cellular component movement;metabolic process;regulation of biological process;response to stimul	cytoplasm;cytoskeleton;cytosol;extracellular;membrane;organelle lumen	metal ion binding;protein binding;structural molecule activity
3191	metabolic process;regulation of biological process	cytoplasm;membrane;nucleus	DNA binding;protein binding;RNA binding
8288	cellular component movement;defense response;metabolic process;regulation of biological process;response to stimulus;transport	extracellular;organelle lumen	antioxidant activity;catalytic activity;metal ion binding
3192	cell differentiation;metabolic process;regulation of biological process;response to stimulus	cell surface;chromosome;cytoplasm;membrane;nucleus;spliceosomal complex	DNA binding;nucleotide binding;protein binding;RNA binding

9555	cell organization and biogenesis;regulation of biological process	chromosome;nucleus	DNA binding;enzyme regulator activity;protein binding
140885	cellular component movement;transport	membrane	protein binding
26986	metabolic process;regulation of biological process	cytoplasm;cytosol;membrane;nucleus;spliceosomal complex	protein binding;RNA binding;translation regulator activity
5898	cell division;cell organization and biogenesis;regulation of biological process;response to stimulus;transport	cell surface;membrane	catalytic activity;nucleotide binding;protein binding
54504	metabolic process		catalytic activity
6036	defense response;metabolic process;response to stimulus;transport	extracellular;organelle lumen;vacuole	catalytic activity
5553	defense response;regulation of biological process;response to stimulus;transport	extracellular	
6421	cell organization and biogenesis;defense response;metabolic process;regulation of biological process;response to stimulus	chromosome;cytoplasm;nucleus	DNA binding;protein binding;RNA binding
7533	cell organization and biogenesis;metabolic process;regulation of biological process;response to stimulus;transport	cytoplasm;cytosol;membrane;mitochondrion	catalytic activity;protein binding
7037	cell differentiation;cell organization and biogenesis;cellular homeostasis;metabolic process;regulation of biological process;response	cell surface;endosome;extracellular;membrane	protein binding;receptor activity;RNA binding;transporter activity
11198	cell organization and biogenesis;metabolic process;regulation of biological process;response to stimulus	chromosome;nucleus	protein binding;RNA binding
708	cell death;cell organization and biogenesis;defense response;metabolic process;regulation of biological process;response to stimulus	cell surface;cytoplasm;cytosol;extracellular;membrane;mitochondrion;nucleus;organelle lumen	protein binding;RNA binding
396	cellular component movement;regulation of biological process;response to stimulus	cytoplasm;cytoskeleton;cytosol	enzyme regulator activity;protein binding
1511	cell organization and biogenesis;defense response;metabolic process;regulation of biological process;response to stimulus;transport	cell surface;extracellular;membrane;nucleus;organelle lumen	catalytic activity;protein binding
51031		mitochondrion	protein binding
22827	cell death;cellular homeostasis;metabolic process;regulation of biological process	nucleus	catalytic activity;DNA binding;protein binding;RNA binding
4926	cell differentiation;cell division;cell organization and biogenesis;metabolic process;regulation of biological process	chromosome;cytoplasm;cytoskeleton;cytosol;membrane;nucleus	protein binding;structural molecule activity
10627	regulation of biological process;response to stimulus	cytosol	metal ion binding;protein binding
4831; 654364	cell organization and biogenesis;cellular homeostasis;metabolic process;regulation of biological process;response to stimulus;transpo	cytoplasm;cytosol;extracellular;membrane;nucleus;organelle lumen	catalytic activity;DNA binding;metal ion binding;nucleotide binding;protein binding
103910; 10627	regulation of biological process;response to stimulus	cytosol	metal ion binding;protein binding
7203	cell organization and biogenesis;metabolic process;regulation of biological process;transport	cytoplasm;cytoskeleton;cytosol;membrane	nucleotide binding;protein binding;RNA binding
7001	cell communication;cellular homeostasis;metabolic process;regulation of biological process;response to stimulus	cytoplasm;cytosol;membrane	antioxidant activity;catalytic activity;protein binding
5887	cell organization and biogenesis;metabolic process;regulation of biological process;response to stimulus	cytoplasm;cytosol;nucleus;proteasome	DNA binding;protein binding
11164	cell organization and biogenesis;metabolic process	cytosol;nucleus	catalytic activity;metal ion binding;protein binding;RNA binding
6181	cell organization and biogenesis;metabolic process;transport	cytosol;membrane;ribosome	protein binding;structural molecule activity
2799	metabolic process;transport	extracellular;organelle lumen;vacuole	catalytic activity;metal ion binding;protein binding
10493	metabolic process;regulation of biological process;transport	cytoplasm;extracellular;membrane;mitochondrion;organelle lumen	catalytic activity;metal ion binding
79180		membrane	metal ion binding;protein binding
4082	cell organization and biogenesis;regulation of biological process	cytoplasm;cytoskeleton;membrane;nucleus	protein binding
8125	cell organization and biogenesis;metabolic process;regulation of biological process;response to stimulus;transport	cytoplasm;endoplasmic reticulum;nucleus	protein binding;RNA binding
3035	cell organization and biogenesis;metabolic process	cytoplasm;cytosol;mitochondrion	catalytic activity;nucleotide binding;protein binding
4637	cellular component movement	cytosol;membrane	catalytic activity;metal ion binding;motor activity;protein binding;structural molecule activity
9255	cell communication;cell death;cellular component movement;defense response;metabolic process;regulation of biological process;res	cell surface;cytoplasm;cytosol;endoplasmic reticulum;extracellular;Golgi;membrane;nucleus	protein binding;RNA binding
25796	metabolic process	cytoplasm;cytosol	catalytic activity;protein binding
3183	cell differentiation;cell organization and biogenesis;metabolic process;regulation of biological process	cytoskeleton;cytosol;extracellular;membrane;nucleus;spliceosomal complex	DNA binding;protein binding;RNA binding
23071	cellular homeostasis;metabolic process;regulation of biological process;response to stimulus;transport	cell surface;endoplasmic reticulum;extracellular;membrane;organelle lumen	catalytic activity;metal ion binding;protein binding
162	regulation of biological process;transport	cytosol;Golgi;membrane	protein binding;transporter activity
5093	metabolic process;regulation of biological process	cytoplasm;cytosol;membrane;nucleus	DNA binding;protein binding;RNA binding
51466	cell organization and biogenesis;cellular component movement;regulation of biological process;response to stimulus	cytoplasm;cytoskeleton;cytosol;membrane	protein binding
100	cell differentiation;metabolic process;regulation of biological process;response to stimulus;transport	cell surface;cytoplasm;cytosol;membrane;organelle lumen;vacuole	catalytic activity;metal ion binding;protein binding
11316	transport	cytoplasm;cytosol;Golgi;membrane	protein binding;structural molecule activity
	regulation of biological process;response to stimulus	endoplasmic reticulum	
1975	cell organization and biogenesis;metabolic process;regulation of biological process	cytosol	catalytic activity;protein binding;RNA binding
3181	metabolic process;regulation of biological process;response to stimulus;transport	cytoplasm;extracellular;membrane;nucleus;spliceosomal complex	DNA binding;protein binding;RNA binding
10007	metabolic process	cytoplasm;cytosol	catalytic activity;protein binding
3674	cell organization and biogenesis;regulation of biological process;response to stimulus;transport	cell surface;membrane	metal ion binding;protein binding
2271	metabolic process	cytoplasm;mitochondrion;organelle lumen	catalytic activity;protein binding
55920	cell division;cell organization and biogenesis;regulation of biological process;response to stimulus	chromosome;cytoplasm;cytoskeleton;cytosol;membrane;nucleus	protein binding;RNA binding
4670	metabolic process;regulation of biological process;response to stimulus	membrane;nucleus;spliceosomal complex	protein binding;RNA binding
6709	cell organization and biogenesis;cellular component movement;metabolic process;regulation of biological process;response to stimul	cytoplasm;cytoskeleton;cytosol;extracellular;membrane;organelle lumen	metal ion binding;protein binding;structural molecule activity
6711	cell organization and biogenesis;cellular component movement;metabolic process;regulation of biological process;response to stimul	cytoplasm;cytoskeleton;cytosol;membrane;nucleus	protein binding;RNA binding;structural molecule activity
2745	cellular homeostasis;metabolic process;regulation of biological process;transport	cytoplasm;cytosol;mitochondrion;nucleus	catalytic activity;protein binding
10606	metabolic process	cytoplasm;cytosol;membrane	catalytic activity;nucleotide binding;protein binding
9131	cell death;cell differentiation;cell organization and biogenesis;cellular homeostasis;metabolic process;regulation of biological process	cytoplasm;cytosol;membrane;mitochondrion;nucleus	catalytic activity;DNA binding;nucleotide binding;protein binding
7416	cell communication;cell death;cell differentiation;defense response;metabolic process;regulation of biological process;response to sti	membrane;mitochondrion;nucleus	protein binding;transporter activity
3185	metabolic process;regulation of biological process;response to stimulus	cytosol;membrane;nucleus;spliceosomal complex	protein binding;RNA binding
5690	metabolic process;regulation of biological process;response to stimulus;transport	cytoplasm;cytosol;membrane;nucleus;proteasome	catalytic activity;enzyme regulator activity;protein binding
27044	cell differentiation;metabolic process;regulation of biological process	cytoplasm;cytosol;membrane;mitochondrion;nucleus	catalytic activity;protein binding;RNA binding
308	coagulation;regulation of biological process;response to stimulus;transport	cytoplasm;cytosol;extracellular;membrane	enzyme regulator activity;metal ion binding;protein binding
5692	metabolic process;regulation of biological process;response to stimulus;transport	cytoplasm;cytosol;nucleus;proteasome	catalytic activity;enzyme regulator activity;protein binding
2171	metabolic process;transport	cytoplasm;cytosol;extracellular;membrane;organelle lumen	protein binding;transporter activity
84823		membrane;nucleus	catalytic activity;motor activity;protein binding;structural molecule activity
10236	metabolic process;regulation of biological process	cytoplasm;endoplasmic reticulum;nucleus;spliceosomal complex	protein binding;RNA binding
2184	metabolic process	cytosol	catalytic activity;metal ion binding;protein binding
6950	cell organization and biogenesis;metabolic process;regulation of biological process;response to stimulus;transport	cytoplasm;cytoskeleton;cytosol;Golgi	nucleotide binding;protein binding;RNA binding
5036	cell proliferation;metabolic process;regulation of biological process;transport	cytoplasm;extracellular;membrane;nucleus;organelle lumen	DNA binding;protein binding;RNA binding
6175	cell organization and biogenesis;metabolic process;regulation of biological process;response to stimulus;transport	cytoplasm;cytosol;endoplasmic reticulum;membrane;nucleus;ribosome	protein binding;RNA binding;structural molecule activity
10935	cell death;cell differentiation;cell organization and biogenesis;cellular homeostasis;metabolic process;regulation of biological process	cytoplasm;cytosol;endosome;mitochondrion;organelle lumen	antioxidant activity;catalytic activity;enzyme regulator activity;protein binding
8880	metabolic process;regulation of biological process	nucleus	DNA binding;protein binding;RNA binding
1509	metabolic process;transport	extracellular;membrane;organelle lumen;vacuole	catalytic activity;protein binding
6741	metabolic process;transport	cytoplasm;nucleus	protein binding;RNA binding
3251	cell differentiation;cell organization and biogenesis;cell proliferation;metabolic process;regulation of biological process;response to sti	cytoplasm;cytosol	catalytic activity;metal ion binding;nucleotide binding;protein binding
10577	metabolic process;regulation of biological process;response to stimulus;transport	endoplasmic reticulum;extracellular;organelle lumen;vacuole	protein binding;transporter activity
7332	cell proliferation;metabolic process;regulation of biological process;response to stimulus	cytoplasm;cytosol;nucleus	catalytic activity;enzyme regulator activity;nucleotide binding;protein binding;RNA binding
3304; 3303	cell organization and biogenesis;metabolic process;regulation of biological process;response to stimulus;transport	cytoplasm;cytoskeleton;cytosol;endoplasmic reticulum;extracellular;mitochondrion;nucleus	catalytic activity;nucleotide binding;protein binding;receptor activity;RNA binding
55968	cell organization and biogenesis;metabolic process;regulation of biological process	chromosome;cytoskeleton;cytosol;Golgi;membrane;nucleus	enzyme regulator activity;protein binding
7919	cell organization and biogenesis;metabolic process;regulation of biological process;transport	cytoplasm;nucleus;spliceosomal complex	catalytic activity;nucleotide binding;protein binding;RNA binding
9948	cell differentiation;cell organization and biogenesis;cellular component movement;metabolic process;regulation of biological process;	cytoplasm;cytoskeleton;cytosol;extracellular	protein binding
6050	metabolic process;regulation of biological process	cytoplasm;cytosol	enzyme regulator activity;protein binding
3799	cell differentiation;cell organization and biogenesis;cellular component movement;metabolic process;regulation of biological process;	cytoplasm;cytoskeleton;cytosol;membrane	catalytic activity;motor activity;nucleotide binding;protein binding
9588	cellular homeostasis;metabolic process;regulation of biological process;response to stimulus;transport	cytoplasm;cytosol;extracellular;membrane;nucleus;organelle lumen;vacuole	antioxidant activity;catalytic activity;protein binding
3417	metabolic process;regulation of biological process;response to stimulus;transport	cytoplasm;cytosol;extracellular;mitochondrion;organelle lumen	catalytic activity;metal ion binding;nucleotide binding;protein binding
5473	cellular component movement;defense response;regulation of biological process;response to stimulus;transport	extracellular;organelle lumen	protein binding;transporter activity
79017	cell organization and biogenesis;metabolic process	cytosol	catalytic activity;protein binding
23193	metabolic process	endoplasmic reticulum;Golgi;membrane;organelle lumen	catalytic activity;RNA binding
2937	metabolic process;response to stimulus	cytosol	catalytic activity;metal ion binding;nucleotide binding;protein binding
7555	metabolic process;regulation of biological process	cytoplasm;cytosol;endoplasmic reticulum;nucleus	DNA binding;metal ion binding;protein binding;RNA binding
387	cell differentiation;cell division;cell organization and biogenesis;cellular component movement;metabolic process;regulation of biologi	cytoplasm;cytoskeleton;cytosol;endosome;membrane	catalytic activity;nucleotide binding;protein binding
3115	regulation of biological process;response to stimulus	cell surface;endoplasmic reticulum;endosome;Golgi;membrane;vacuole	
25939	cell organization and biogenesis;defense response;metabolic process;regulation of biological process;response to stimulus	membrane;nucleus	catalytic activity;metal ion binding;nucleotide binding;protein binding;RNA binding
3068	cell proliferation;metabolic process;regulation of biological process;response to stimulus	cytoplasm;extracellular;nucleus	DNA binding;nucleotide binding;protein binding;RNA binding
11151	cell organization and biogenesis;cellular component movement;defense response;regulation of biological process;response to stimulu	cytoplasm;cytoskeleton;cytosol;endosome;membrane;nucleus	protein binding;RNA binding
55276	metabolic process;transport	cytoplasm;cytosol;extracellular;organelle lumen	catalytic activity;metal ion binding;protein binding
5691	metabolic process;regulation of biological process;response to stimulus;transport	cytoplasm;cytosol;nucleus;proteasome	catalytic activity;enzyme regulator activity;protein binding
4841	defense response;metabolic process;regulation of biological process;response to stimulus	membrane;nucleus	DNA binding;protein binding;RNA binding
3959	defense response;regulation of biological process;response to stimulus;transport	extracellular;membrane;organelle lumen	protein binding;receptor activity
2539	metabolic process;regulation of biological process;response to stimulus	cytoplasm;cytosol;membrane;nucleus	catalytic activity;nucleotide binding;protein binding
7112	regulation of biological process	chromosome;nucleus	DNA binding;protein binding
6634	cell organization and biogenesis;metabolic process;transport	cytoplasm;cytosol;nucleus;spliceosomal complex	protein binding;RNA binding
760	cellular homeostasis;metabolic process;regulation of biological process;response to stimulus;transport	cytoplasm;cytosol;membrane	catalytic activity;metal ion binding;protein binding
1981	cell organization and biogenesis;defense response;metabolic process;regulation of biological process;response to stimulus	cytoplasm;cytosol;membrane;nucleus	nucleotide binding;protein binding;RNA binding;structural molecule activity

10575	metabolic process;regulation of biological process;transport	cytoplasm;cytoskeleton;cytosol	nucleotide binding;protein binding;RNA binding
5271	regulation of biological process	cytoplasm;cytosol	enzyme regulator activity;protein binding
5510	regulation of biological process	chromosome;cytoplasm;nucleus	enzyme regulator activity;protein binding
1075	cell death;cell organization and biogenesis;metabolic process;regulation of biological process;response to stimulus;transport	endoplasmic reticulum;extracellular;Golgi;membrane;organelle lumen;vacuole	catalytic activity;enzyme regulator activity;protein binding
3958	cell differentiation;cellular component movement;defense response;metabolic process;regulation of biological process;response to sti	cytoplasm;extracellular;membrane;nucleus;spliceosomal complex	protein binding;RNA binding
5878	regulation of biological process;response to stimulus;transport	endosome;membrane	catalytic activity;nucleotide binding;protein binding
2950	cell communication;cell proliferation;metabolic process;regulation of biological process;response to stimulus;transport	cytoplasm;cytosol;extracellular;membrane;mitochondrion;nucleus;organelle lumen	antioxidant activity;catalytic activity;enzyme regulator activity;protein binding
22919	cell division;cell organization and biogenesis;cell proliferation;regulation of biological process	cytoplasm;cytoskeleton;cytosol;Golgi	protein binding;RNA binding
9559	regulation of biological process;response to stimulus;transport	cytoplasm;cytosol;endosome;membrane;vacuole	protein binding;transporter activity
8775	cell communication;cell differentiation;cell organization and biogenesis;regulation of biological process;transport	cytosol;membrane	protein binding
51411	cell organization and biogenesis;cellular component movement;response to stimulus;transport	cytoplasm;extracellular;membrane;organelle lumen	protein binding
23020	cell differentiation;cell organization and biogenesis;metabolic process	membrane;nucleus;spliceosomal complex	catalytic activity;DNA binding;nucleotide binding;protein binding;RNA binding
5245	cell differentiation;cell organization and biogenesis;metabolic process;regulation of biological process;response to stimulus;transport	cell surface;cytoplasm;endosome;membrane;mitochondrion;nucleus	DNA binding;protein binding
11335; 90416	cell organization and biogenesis;metabolic process;regulation of biological process	chromosome;membrane;nucleus	protein binding
3849	cell death;cell organization and biogenesis;cell proliferation;cellular component movement;metabolic process	cytoskeleton;cytosol;membrane;nucleus	protein binding;structural molecule activity
1973	metabolic process;regulation of biological process	cytoplasm;cytosol;membrane;nucleus	catalytic activity;DNA binding;nucleotide binding;protein binding;RNA binding
56005	cell death;regulation of biological process	extracellular;organelle lumen	protein binding
10525	regulation of biological process;response to stimulus;transport	endoplasmic reticulum;extracellular;membrane;organelle lumen	nucleotide binding;protein binding
6628	cell organization and biogenesis;metabolic process;transport	cytoplasm;cytosol;nucleus;spliceosomal complex	protein binding;RNA binding
6189	cell differentiation;cell organization and biogenesis;metabolic process;regulation of biological process;transport	cytoplasm;cytosol;endoplasmic reticulum;nucleus;ribosome	protein binding;RNA binding;structural molecule activity
3105	regulation of biological process;response to stimulus	cell surface;endoplasmic reticulum;Golgi;membrane	protein binding;RNA binding
10327	metabolic process	cytosol;membrane	catalytic activity;protein binding
7178	cell differentiation;cellular homeostasis;regulation of biological process;response to stimulus;transport	cytoplasm;cytosol;endosome;nucleus	metal ion binding;protein binding;RNA binding
8662	cell organization and biogenesis;metabolic process;regulation of biological process	cytoplasm;cytosol	protein binding;RNA binding;structural molecule activity
3848	cell death;defense response;metabolic process;regulation of biological process;response to stimulus;transport	cytoskeleton;cytosol;extracellular;membrane;nucleus	catalytic activity;motor activity;protein binding;receptor activity;structural molecule activity
8338	regulation of biological process	chromosome;nucleus	DNA binding;protein binding
4052	metabolic process;regulation of biological process;response to stimulus	cell surface;extracellular;membrane;organelle lumen	catalytic activity;metal ion binding;protein binding;receptor activity;signal transducer activity
9126	cell division;cell organization and biogenesis;metabolic process;regulation of biological process;response to stimulus;transport	chromosome;cytosol;nucleus	catalytic activity;motor activity;nucleotide binding;protein binding;transporter activity
7155	cell organization and biogenesis;cellular component movement;metabolic process;regulation of biological process	cytoplasm;cytosol;nucleus	catalytic activity;DNA binding;metal ion binding;nucleotide binding;protein binding
23406	defense response;response to stimulus;transport	cytoplasm;cytoskeleton;extracellular;nucleus;organelle lumen	protein binding
3482	cell organization and biogenesis;regulation of biological process;response to stimulus;transport	cell surface;cytoplasm;endosome;Golgi;membrane;nucleus;vacuole	catalytic activity;protein binding;receptor activity;signal transducer activity;transporter activity
5931	cell organization and biogenesis;cell proliferation;development;metabolic process;regulation of biological process;response to stimul	cytosol;nucleus	catalytic activity;protein binding;RNA binding
2805	metabolic process;regulation of biological process;response to stimulus	cytoplasm;cytosol;mitochondrion;nucleus;vacuole	catalytic activity
7873	cell differentiation;cell organization and biogenesis;regulation of biological process;response to stimulus;transport	cytosol;endoplasmic reticulum;extracellular;nucleus	protein binding;RNA binding
9967	metabolic process;regulation of biological process;response to stimulus	nucleus	DNA binding;nucleotide binding;protein binding;receptor activity;RNA binding
3690	cell differentiation;cell growth;cell organization and biogenesis;cellular component movement;coagulation;regulation of biological proc	cell surface;membrane;nucleus	catalytic activity;protein binding;receptor activity;signal transducer activity
6500	cell organization and biogenesis;cellular homeostasis;metabolic process;regulation of biological process;response to stimulus	cytoplasm;cytosol;nucleus	catalytic activity;protein binding
55700	cell organization and biogenesis	cytoplasm;cytoskeleton;cytosol	structural molecule activity
4013			
567	cell differentiation;defense response;metabolic process;regulation of biological process;response to stimulus;transport	cell surface;cytosol;extracellular;Golgi;membrane;organelle lumen	protein binding
143244	metabolic process;regulation of biological process;transport	cytoplasm;endoplasmic reticulum;membrane;nucleus	RNA binding
5868	cell organization and biogenesis;coagulation;metabolic process;regulation of biological process;response to stimulus;transport	cytoplasm;cytoskeleton;cytosol;endosome;membrane	catalytic activity;nucleotide binding;protein binding
5478	cell organization and biogenesis;cellular component movement;metabolic process;regulation of biological process;response to stimul	cytoplasm;cytosol;extracellular;membrane;nucleus;organelle lumen	catalytic activity;protein binding;RNA binding
3916	cell death;regulation of biological process;response to stimulus;transport	cell surface;cytoplasm;cytosol;endosome;membrane;vacuole	protein binding;receptor activity
10923	cell organization and biogenesis;metabolic process;regulation of biological process;response to stimulus	nucleus	catalytic activity;DNA binding;protein binding;RNA binding
1457	cell communication;cell death;metabolic process;regulation of biological process;response to stimulus	chromosome;cytoplasm;cytosol;membrane;nucleus	catalytic activity;enzyme regulator activity;nucleotide binding;protein binding
823	cell death;cell organization and biogenesis;metabolic process;regulation of biological process;transport	cytoplasm;cytosol;extracellular;membrane;mitochondrion;vacuole	catalytic activity;metal ion binding;protein binding
8243	cell division;cell organization and biogenesis;metabolic process;regulation of biological process;response to stimulus	chromosome;cytosol;nucleus	nucleotide binding;protein binding;RNA binding
2026	metabolic process	cytoplasm;cytosol;membrane	catalytic activity;metal ion binding;protein binding
51571	regulation of biological process;transport	extracellular;membrane;organelle lumen	protein binding
3956	cell death;cell differentiation;cellular homeostasis;metabolic process;regulation of biological process;response to stimulus	cell surface;cytoplasm;cytosol;extracellular;nucleus;organelle lumen	protein binding;RNA binding;signal transducer activity
6601	cell organization and biogenesis;metabolic process;regulation of biological process	nucleus	DNA binding;protein binding
27101	cell differentiation;regulation of biological process;response to stimulus	cytoplasm;cytosol;nucleus	protein binding
214	cell growth;cellular component movement;regulation of biological process;response to stimulus	extracellular;membrane	protein binding
3507	cell organization and biogenesis;cellular component movement;defense response;metabolic process;regulation of biological process;	cell surface;extracellular;membrane	DNA binding;protein binding
83442	cellular homeostasis;metabolic process;regulation of biological process	cytoplasm;nucleus	catalytic activity
10539	cell organization and biogenesis;cellular homeostasis;metabolic process;regulation of biological process	cytoplasm;cytosol;nucleus;spliceosomal complex	catalytic activity;metal ion binding;protein binding;RNA binding
10576	cell organization and biogenesis;metabolic process;regulation of biological process;transport	cytoplasm;cytosol;extracellular;organelle lumen	nucleotide binding;protein binding
6285	cell differentiation;cell organization and biogenesis;cell proliferation;defense response;regulation of biological process;response to sti	cytoplasm;extracellular;nucleus	metal ion binding;protein binding
51292	metabolic process	cytosol	catalytic activity;metal ion binding
3149	defense response;development;metabolic process;regulation of biological process;response to stimulus	chromosome;cytoplasm;nucleus	DNA binding;protein binding;RNA binding
976	cell communication;cellular component movement;defense response;regulation of biological process;response to stimulus;transport	cell surface;extracellular;membrane	metal ion binding;protein binding;receptor activity;signal transducer activity
51631	cell organization and biogenesis	nucleus;spliceosomal complex	protein binding;RNA binding
2027	metabolic process	cytoplasm;cytosol;membrane	catalytic activity;metal ion binding
2885	cell communication;cell differentiation;cell organization and biogenesis;cellular component movement;defense response;metabolic pr	cytoplasm;cytosol;endosome;Golgi;membrane;nucleus	catalytic activity;protein binding;RNA binding
4666	metabolic process;regulation of biological process	cytoplasm;nucleus	DNA binding
5273	regulation of biological process;transport	cytoplasm;cytosol;membrane;nucleus	enzyme regulator activity
2288	cell organization and biogenesis;metabolic process;regulation of biological process;response to stimulus;transport	cytoplasm;cytoskeleton;cytosol;mitochondrion;nucleus	catalytic activity;nucleotide binding;protein binding;RNA binding
1520	cell organization and biogenesis;metabolic process;regulation of biological process;response to stimulus;transport	extracellular;organelle lumen;vacuole	catalytic activity;protein binding
22820	cellular component movement;transport	cytoplasm;cytosol;Golgi;membrane	protein binding;structural molecule activity
5589	metabolic process;regulation of biological process;response to stimulus	endoplasmic reticulum;organelle lumen	metal ion binding;protein binding
516	cell organization and biogenesis;metabolic process;transport	membrane;mitochondrion	catalytic activity;protein binding;transporter activity
4172	metabolic process	cytoplasm;membrane;nucleus	catalytic activity;DNA binding;nucleotide binding;protein binding
1660	cell death;cell differentiation;cell organization and biogenesis;defense response;metabolic process;regulation of biological process;re	cytoplasm;cytoskeleton;cytosol;membrane;nucleus;ribosome	catalytic activity;DNA binding;metal ion binding;nucleotide binding;protein binding;RNA binding
5781	cell differentiation;cell organization and biogenesis;cellular component movement;metabolic process;regulation of biological process;	cytoplasm;cytosol;mitochondrion;nucleus	catalytic activity;protein binding
389898	metabolic process;response to stimulus	cytoplasm;nucleus	catalytic activity;protein binding
1738	cellular homeostasis;metabolic process;regulation of biological process	mitochondrion;organelle lumen	catalytic activity;nucleotide binding;protein binding
2876	cell death;cell differentiation;cell proliferation;cellular component movement;cellular homeostasis;metabolic process;regulation of biol	cytoplasm;cytosol;mitochondrion;organelle lumen	antioxidant activity;catalytic activity;protein binding
2517	metabolic process;transport	cytoplasm;extracellular;organelle lumen;vacuole	catalytic activity
831	regulation of biological process	cytosol;endoplasmic reticulum;membrane	enzyme regulator activity;protein binding;RNA binding
8635	metabolic process;transport	endoplasmic reticulum;extracellular;organelle lumen;vacuole	catalytic activity;RNA binding
3608	metabolic process;regulation of biological process;response to stimulus;transport	cytoplasm;extracellular;membrane;nucleus;organelle lumen	catalytic activity;DNA binding;nucleotide binding;protein binding;RNA binding
728689	metabolic process	cytoplasm	protein binding;RNA binding
8661	cell organization and biogenesis;metabolic process;regulation of biological process	cytoplasm;cytosol;membrane;nucleus	protein binding;RNA binding;structural molecule activity
10989	cell organization and biogenesis;cellular homeostasis	membrane;mitochondrion	protein binding;RNA binding
5524	cell organization and biogenesis;metabolic process;regulation of biological process	cytoplasm;nucleus	catalytic activity;enzyme regulator activity;nucleotide binding;protein binding
327	cell organization and biogenesis;metabolic process;transport	cytoplasm;cytosol;extracellular;membrane	catalytic activity;protein binding;RNA binding
2317	cell differentiation;cell organization and biogenesis;development;regulation of biological process;response to stimulus	cytoplasm;cytoskeleton;cytosol;membrane;nucleus	protein binding;RNA binding
3151		chromosome;cytoplasm;nucleus	DNA binding;protein binding;RNA binding
9782	defense response;regulation of biological process;response to stimulus	membrane;nucleus	metal ion binding;protein binding;RNA binding;structural molecule activity
4043	regulation of biological process;transport	cell surface;cytoplasm;endoplasmic reticulum;endosome;extracellular;Golgi;membrane;organelle lumen	protein binding
1991	cell organization and biogenesis;cellular component movement;cellular homeostasis;defense response;metabolic process;regulation	cell surface;cytoplasm;extracellular;organelle lumen	catalytic activity;protein binding
5516	cell organization and biogenesis;metabolic process;regulation of biological process;response to stimulus	chromosome;cytoplasm;cytoskeleton;cytosol;nucleus	catalytic activity;metal ion binding;protein binding
4076	cell differentiation;regulation of biological process	cytoplasm;cytosol;membrane	protein binding;RNA binding
10574	metabolic process;regulation of biological process;transport	cytoplasm;cytosol;mitochondrion	nucleotide binding;protein binding
64167	cell communication;metabolic process;regulation of biological process;response to stimulus	endoplasmic reticulum;membrane;organelle lumen	catalytic activity;metal ion binding
8411	cell organization and biogenesis;metabolic process;transport	cytoplasm;cytosol;endosome;membrane	catalytic activity;metal ion binding;motor activity;protein binding;transporter activity
9208	metabolic process;regulation of biological process	cytoplasm;cytoskeleton;cytosol;membrane;nucleus	DNA binding;protein binding;RNA binding
163	cell organization and biogenesis;cellular component movement;metabolic process;regulation of biological process;response to stimul	cytosol;membrane	protein binding;transporter activity
440686	cell organization and biogenesis;regulation of biological process	chromosome;nucleus	DNA binding;protein binding
4678	cell organization and biogenesis;cell proliferation;metabolic process;response to stimulus;transport	cytoplasm;nucleus	protein binding

10521	cell differentiation;cell organization and biogenesis;defense response;metabolic process;regulation of biological process;response to	cytoplasm;cytosol;membrane;nucleus	catalytic activity;nucleotide binding;protein binding;RNA binding
6917	metabolic process;regulation of biological process;response to stimulus	nucleus	DNA binding;metal ion binding;protein binding
10437	metabolic process;regulation of biological process;response to stimulus	cytosol;extracellular;organelle lumen;vacuole	catalytic activity;protein binding
2760	metabolic process;regulation of biological process;response to stimulus;transport	cytoplasm;extracellular;mitochondrion;organelle lumen;vacuole	catalytic activity;enzyme regulator activity;transporter activity
7536	cell differentiation;cell organization and biogenesis;metabolic process;regulation of biological process	nucleus;ribosome;spliceosomal complex	metal ion binding;protein binding;RNA binding
9793	cell division;cell organization and biogenesis;regulation of biological process;transport	chromosome;cytoplasm;cytoskeleton;cytosol;membrane	protein binding
821	metabolic process;regulation of biological process;response to stimulus;transport	cytoplasm;endoplasmic reticulum;membrane;organelle lumen;ribosome	metal ion binding;protein binding;RNA binding
6125	cell organization and biogenesis;metabolic process;regulation of biological process;transport	cytoplasm;cytosol;membrane;nucleus;ribosome	protein binding;RNA binding;structural molecule activity
1778	cell division;cell organization and biogenesis;cellular component movement;regulation of biological process;transport	cytoplasm;cytoskeleton;cytosol;extracellular;membrane;organelle lumen	catalytic activity;motor activity;nucleotide binding;protein binding;RNA binding
6451	cellular homeostasis;regulation of biological process	cytoplasm;nucleus	catalytic activity;protein binding
10095	cell organization and biogenesis;cellular component movement;regulation of biological process;response to stimulus	cytoplasm;cytoskeleton;cytosol	protein binding;structural molecule activity
6275	cell organization and biogenesis;regulation of biological process	extracellular;nucleus	metal ion binding;protein binding;RNA binding
427	metabolic process;transport	extracellular;organelle lumen;vacuole	catalytic activity
126133	metabolic process	membrane	catalytic activity
11021	cell organization and biogenesis;regulation of biological process;response to stimulus;transport	cytosol;endosome;membrane;mitochondrion	catalytic activity;nucleotide binding;protein binding
4175	metabolic process	nucleus	catalytic activity;DNA binding;nucleotide binding;protein binding
830	cell organization and biogenesis;cellular component movement;coagulation;defense response;regulation of biological process;respon	cytoskeleton;cytosol;extracellular;membrane	protein binding
10549	cell organization and biogenesis;cellular homeostasis;metabolic process;regulation of biological process;transport	cytoplasm;cytosol;endoplasmic reticulum;extracellular;mitochondrion;nucleus;organelle lumen	antioxidant activity;catalytic activity;protein binding
6202	cell organization and biogenesis;metabolic process;transport	cytoplasm;cytosol;endoplasmic reticulum;membrane;nucleus;ribosome	protein binding;RNA binding;structural molecule activity
11345	cell communication;cell organization and biogenesis;metabolic process;regulation of biological process;response to stimulus;transpor	cytoplasm;cytosol;Golgi;membrane;vacuole	protein binding
7317	metabolic process;response to stimulus	cytoplasm;cytosol;membrane;mitochondrion;nucleus	catalytic activity;nucleotide binding;protein binding;RNA binding
51816	development;metabolic process;regulation of biological process;transport	cytosol;extracellular;organelle lumen	catalytic activity;metal ion binding;protein binding
8836	metabolic process;response to stimulus;transport	cytosol;extracellular;nucleus;organelle lumen;vacuole	catalytic activity
2040	cell organization and biogenesis;regulation of biological process;transport	cytoplasm;cytoskeleton;cytosol;endoplasmic reticulum;membrane;mitochondrion	protein binding
6426	cell differentiation;cell organization and biogenesis;metabolic process;regulation of biological process;transport	cytoplasm;nucleus;spliceosomal complex	protein binding;RNA binding
8761	coagulation;metabolic process	cytoplasm;cytosol;nucleus	protein binding;RNA binding
5265	cell organization and biogenesis;coagulation;defense response;metabolic process;regulation of biological process;response to stimul	endoplasmic reticulum;extracellular;Golgi;membrane;organelle lumen	enzyme regulator activity;protein binding
989	cell differentiation;cell division;cell organization and biogenesis;regulation of biological process	chromosome;cytoplasm;cytoskeleton;cytosol;membrane;nucleus	nucleotide binding;protein binding;structural molecule activity
5204	metabolic process;regulation of biological process	cytoplasm;nucleus	protein binding
92241	response to stimulus		protein binding
4738	cellular homeostasis;metabolic process;regulation of biological process;response to stimulus	cytosol;nucleus	protein binding
5886	metabolic process;regulation of biological process;response to stimulus	cytoplasm;cytosol;nucleus;proteasome	DNA binding;protein binding
6139	cell organization and biogenesis;metabolic process;transport	cytosol;nucleus;ribosome	protein binding;RNA binding;structural molecule activity
6897	metabolic process	cytoplasm;cytoskeleton;cytosol	catalytic activity;nucleotide binding;protein binding;RNA binding
11054	regulation of biological process;response to stimulus	cytoplasm;extracellular;membrane;nucleus	protein binding;receptor activity;signal transducer activity
51752	cell communication;cell differentiation;metabolic process;regulation of biological process;response to stimulus	cytoplasm;cytosol;endoplasmic reticulum;extracellular;membrane;organelle lumen	catalytic activity;metal ion binding;protein binding
3118	regulation of biological process;response to stimulus	endoplasmic reticulum;endosome;Golgi;membrane;vacuole	receptor activity;signal transducer activity
2962	metabolic process;regulation of biological process;response to stimulus	nucleus	catalytic activity;DNA binding;enzyme regulator activity;protein binding;RNA binding
7257	cell differentiation;development;metabolic process;response to stimulus;transport	cytoplasm;cytosol;Golgi;nucleus	DNA binding;metal ion binding;protein binding;RNA binding;transporter activity
8815	cell organization and biogenesis;metabolic process;regulation of biological process;response to stimulus;transport	chromosome;cytoplasm;cytosol;nucleus	DNA binding;protein binding
310	cell differentiation;cell organization and biogenesis;cell proliferation;cellular homeostasis;metabolic process;regulation of biological pr	cytosol;membrane;nucleus	metal ion binding;protein binding;RNA binding
3295	cell differentiation;metabolic process	membrane;mitochondrion;organelle lumen	catalytic activity;protein binding
4735	cell differentiation;cell division;cell organization and biogenesis;regulation of biological process;response to stimulus	chromosome;cytoplasm;cytoskeleton;cytosol;membrane;nucleus	nucleotide binding;protein binding
3150	cell organization and biogenesis;metabolic process;regulation of biological process;response to stimulus	chromosome;cytoplasm;nucleus	DNA binding;protein binding
6251	regulation of biological process;response to stimulus	cytosol	protein binding
10189	cell differentiation;metabolic process;regulation of biological process;response to stimulus;transport	cytoplasm;cytosol;membrane;nucleus;spliceosomal complex	protein binding;RNA binding
8721	cell differentiation;development;metabolic process;regulation of biological process	cytoplasm;cytosol;nucleus	catalytic activity;DNA binding;protein binding;RNA binding
160	cell organization and biogenesis;cellular component movement;metabolic process;regulation of biological process;response to stimul	cytosol;membrane	protein binding;transporter activity
54039	metabolic process;regulation of biological process	cytoplasm;cytosol;nucleus	DNA binding;RNA binding
25824	cell death;cellular homeostasis;defense response;metabolic process;regulation of biological process;response to stimulus	cytoplasm;cytosol;mitochondrion;nucleus;organelle lumen	antioxidant activity;catalytic activity;DNA binding;enzyme regulator activity;protein binding
23157	cell differentiation;cell division	chromosome;cytoplasm;cytoskeleton	nucleotide binding;protein binding
84064	metabolic process		catalytic activity;metal ion binding;protein binding
11337	cell communication;cell death;cell organization and biogenesis;metabolic process;regulation of biological process;response to stimul	cytoplasm;cytoskeleton;cytosol;endoplasmic reticulum;Golgi;membrane;vacuole	protein binding
8570	metabolic process;regulation of biological process;response to stimulus;transport	cytoplasm;cytosol;membrane;nucleus	DNA binding;protein binding;RNA binding
4904	metabolic process;regulation of biological process	cytoplasm;cytosol;extracellular;membrane;nucleus;spliceosomal complex	DNA binding;protein binding;RNA binding
2935	cell organization and biogenesis;metabolic process	cytosol	catalytic activity;nucleotide binding;protein binding;RNA binding
56729	cell differentiation;regulation of biological process;response to stimulus;transport	extracellular;nucleus;organelle lumen	protein binding
5518	cell death;cell organization and biogenesis;metabolic process;regulation of biological process;response to stimulus	chromosome;cytoplasm;cytoskeleton;cytosol;membrane;mitochondrion;nucleus	catalytic activity;enzyme regulator activity;protein binding
5499	cell division;metabolic process;regulation of biological process;response to stimulus	cytoplasm;cytosol;membrane;nucleus	catalytic activity;metal ion binding;protein binding
10409	cell differentiation;cell organization and biogenesis;regulation of biological process	cytoplasm;cytoskeleton;membrane;nucleus	DNA binding;protein binding
6185	metabolic process;response to stimulus	endoplasmic reticulum;membrane	catalytic activity;protein binding
6386	cell communication;cell organization and biogenesis;regulation of biological process;response to stimulus;transport	cytoplasm;cytoskeleton;cytosol;endoplasmic reticulum;extracellular;membrane;nucleus;organelle lumen	protein binding
566	cellular component movement;defense response;metabolic process;regulation of biological process;response to stimulus;transport	extracellular;membrane;organelle lumen;vacuole	catalytic activity;protein binding
5094	defense response;metabolic process;regulation of biological process;response to stimulus	cytoplasm;cytosol;membrane;nucleus	DNA binding;protein binding;RNA binding
11331	cell organization and biogenesis;metabolic process;regulation of biological process;response to stimulus;transport	cell surface;cytoplasm;membrane;mitochondrion;nucleus	protein binding
11338	metabolic process;regulation of biological process;transport	nucleus;spliceosomal complex	protein binding;RNA binding
6130	cell organization and biogenesis;metabolic process;transport	cytoplasm;cytosol;membrane;nucleus;ribosome	protein binding;RNA binding;structural molecule activity
908	metabolic process;regulation of biological process	cytoplasm;cytosol;membrane	nucleotide binding;protein binding;RNA binding
3187	metabolic process;regulation of biological process;response to stimulus	cytosol;membrane;nucleus;spliceosomal complex	protein binding;RNA binding
9939	metabolic process;regulation of biological process;transport	cytoplasm;cytosol;nucleus;spliceosomal complex	protein binding;RNA binding
1178	cell death;development;regulation of biological process	cytoplasm;cytosol	catalytic activity;protein binding
4736	cell organization and biogenesis;metabolic process;response to stimulus;transport	cytosol;membrane;nucleus;ribosome	protein binding;RNA binding;structural molecule activity
10262	metabolic process;regulation of biological process	nucleus;spliceosomal complex	protein binding;RNA binding
1314	regulation of biological process;transport	cytoplasm;cytosol;extracellular;Golgi;membrane	protein binding;structural molecule activity
5928	cell organization and biogenesis;metabolic process;regulation of biological process;response to stimulus	cytosol;nucleus	catalytic activity;DNA binding;protein binding
10059	cell death;cell organization and biogenesis;regulation of biological process;response to stimulus;transport	cytoplasm;cytoskeleton;cytosol;endoplasmic reticulum;Golgi;membrane;mitochondrion	catalytic activity;enzyme regulator activity;nucleotide binding;protein binding
8971	cell organization and biogenesis	chromosome;nucleus	DNA binding;protein binding;RNA binding
1508	cell differentiation;metabolic process;regulation of biological process;response to stimulus;transport	extracellular;mitochondrion;organelle lumen;vacuole	catalytic activity;protein binding
5110	metabolic process	cytoplasm;cytosol	catalytic activity;protein binding
22929	metabolic process	cytoplasm;membrane;nucleus	catalytic activity;nucleotide binding;protein binding
2806	metabolic process;response to stimulus;transport	cell surface;membrane;mitochondrion;organelle lumen	catalytic activity;protein binding;RNA binding
813	metabolic process	endoplasmic reticulum;extracellular;Golgi;membrane;organelle lumen	metal ion binding;protein binding
8804	cell proliferation;development;regulation of biological process;transport	extracellular;organelle lumen	protein binding
1431	metabolic process	mitochondrion;nucleus;organelle lumen	catalytic activity;RNA binding
3514	cell organization and biogenesis;cellular component movement;defense response;metabolic process;regulation of biological process;	extracellular;membrane	catalytic activity;protein binding
6224	cell organization and biogenesis;metabolic process;transport	cytoplasm;cytosol;membrane;ribosome	protein binding;RNA binding;structural molecule activity
5464	metabolic process	cytoplasm;cytosol	catalytic activity;metal ion binding
10808	metabolic process;regulation of biological process;response to stimulus;transport	cytoplasm;cytosol;extracellular;nucleus;organelle lumen	enzyme regulator activity;nucleotide binding;protein binding
200081	cell proliferation;regulation of biological process;transport	cytoplasm;cytosol;extracellular;membrane	protein binding
1729	cell organization and biogenesis;regulation of biological process;response to stimulus;transport	cytoplasm;cytoskeleton;cytosol;membrane	catalytic activity;motor activity;protein binding;RNA binding
11140	cell division;metabolic process;regulation of biological process;response to stimulus;transport	cytoplasm;cytosol	catalytic activity;enzyme regulator activity;protein binding
9352	cellular homeostasis;metabolic process;regulation of biological process;response to stimulus;transport	cytoplasm;cytosol;nucleus;proteasome	antioxidant activity;catalytic activity
4050	cell communication;metabolic process;regulation of biological process;response to stimulus	membrane	protein binding
9218	cell death;cell organization and biogenesis;metabolic process;regulation of biological process;response to stimulus;transport	cytoskeleton;endoplasmic reticulum;membrane;nucleus	protein binding;signal transducer activity
3313	cell differentiation;cell organization and biogenesis;metabolic process;regulation of biological process;response to stimulus	cytoplasm;mitochondrion;nucleus;organelle lumen	nucleotide binding;protein binding;RNA binding
311	cell division;response to stimulus;transport	cytoplasm;cytoskeleton;cytosol;membrane;nucleus;vacuole	metal ion binding;protein binding;RNA binding
2739	cell differentiation;metabolic process;regulation of biological process	cytoplasm;cytosol;membrane;nucleus	catalytic activity;metal ion binding
11051	cell organization and biogenesis;metabolic process	nucleus	catalytic activity;protein binding;RNA binding
4668	metabolic process	cytoplasm;vacuole	catalytic activity;protein binding
6632	cell organization and biogenesis;metabolic process;transport	cytoplasm;cytosol;nucleus;spliceosomal complex	protein binding;RNA binding
7266	metabolic process;regulation of biological process	cytoplasm;cytoskeleton;cytosol;membrane;nucleus	enzyme regulator activity;protein binding
5356	cell organization and biogenesis;metabolic process;regulation of biological process	membrane;nucleus;spliceosomal complex	catalytic activity;protein binding

767558; 136319	cell differentiation;cell organization and biogenesis;metabolic process;regulation of biological process;response to stimulus	cytoplasm;cytosol;nucleus	DNA binding;protein binding
8209; 102724023		mitochondrion	
7411	cell organization and biogenesis;metabolic process	cytoplasm;cytosol;nucleus	protein binding
64710	cell organization and biogenesis;cellular homeostasis;metabolic process;regulation of biological process;response to stimulus	chromosome;cytoplasm;nucleus	DNA binding;protein binding;RNA binding
5699	metabolic process;regulation of biological process;response to stimulus;transport	cytoplasm;cytosol;nucleus;proteasome	catalytic activity;protein binding
3920	cell communication;cell organization and biogenesis;cellular homeostasis;metabolic process;regulation of biological process;response	endosome;membrane;organelle lumen;vacuole	protein binding
57092	metabolic process	nucleus	protein binding
7247	metabolic process;response to stimulus	cytoplasm;cytosol;nucleus	catalytic activity;DNA binding;protein binding;RNA binding
6137	cell organization and biogenesis;metabolic process;transport	cytosol;membrane;nucleus;ribosome	protein binding;RNA binding;structural molecule activity
56954	metabolic process;transport	cytoplasm;cytosol;extracellular;mitochondrion;organelle lumen	catalytic activity
9097	cell communication;metabolic process;regulation of biological process	cell surface;cytoplasm;cytosol;membrane;nucleus;proteasome	catalytic activity;enzyme regulator activity;protein binding
3073	metabolic process	membrane;organelle lumen;vacuole	catalytic activity;protein binding
6902	cell organization and biogenesis;metabolic process	cytoplasm;cytoskeleton	protein binding;RNA binding
6294	cell organization and biogenesis;metabolic process;regulation of biological process;response to stimulus	nucleus	DNA binding;protein binding;RNA binding
23244	cell division;cell organization and biogenesis;metabolic process;regulation of biological process;response to stimulus	chromosome;cytosol;membrane;nucleus	protein binding
3500	cell organization and biogenesis;cellular component movement;defense response;metabolic process;regulation of biological process;	extracellular;membrane	catalytic activity;metal ion binding;protein binding
2162	coagulation;metabolic process;response to stimulus;transport	cytoplasm;extracellular;organelle lumen	catalytic activity;metal ion binding
5757	cell organization and biogenesis;metabolic process	cytosol;nucleus	protein binding
2633	cell organization and biogenesis;defense response;regulation of biological process;response to stimulus	cytoplasm;cytosol;extracellular;Golgi;membrane	catalytic activity;nucleotide binding;protein binding
10399	cell death;cellular homeostasis;development;metabolic process;regulation of biological process;response to stimulus	cytoplasm;cytosol;membrane;mitochondrion;nucleus;ribosome	enzyme regulator activity;protein binding;RNA binding;structural molecule activity
10938	cell organization and biogenesis;coagulation;regulation of biological process;response to stimulus;transport	endosome;membrane	metal ion binding;nucleotide binding;protein binding
8260	cell organization and biogenesis;metabolic process;regulation of biological process	cytoplasm;cytosol;membrane;nucleus;ribosome	catalytic activity;protein binding
22948	metabolic process;regulation of biological process;response to stimulus;transport	cytoplasm;cytoskeleton;cytosol	nucleotide binding;protein binding;RNA binding
27131	cell organization and biogenesis;regulation of biological process;transport	cytoplasm;cytosol;endosome;membrane	protein binding
9111	defense response;metabolic process;regulation of biological process;response to stimulus	cytoplasm;cytosol	protein binding
10695	defense response;regulation of biological process;response to stimulus	endoplasmic reticulum;organelle lumen	protein binding
6418	cell organization and biogenesis;metabolic process;regulation of biological process;transport	cytoplasm;cytosol;endoplasmic reticulum;nucleus	DNA binding;enzyme regulator activity;protein binding
5530	cellular homeostasis;metabolic process;regulation of biological process;response to stimulus;transport	cytosol;membrane;mitochondrion;nucleus	catalytic activity;metal ion binding;protein binding
27316	cell differentiation;cell organization and biogenesis;metabolic process;regulation of biological process;response to stimulus	membrane;nucleus;spliceosomal complex	DNA binding;protein binding;RNA binding
1108	cell organization and biogenesis;metabolic process;regulation of biological process	cytoplasm;cytoskeleton;membrane;nucleus	catalytic activity;DNA binding;metal ion binding;nucleotide binding;protein binding
9987	metabolic process;regulation of biological process;response to stimulus	cytoplasm;cytosol;nucleus;spliceosomal complex	DNA binding;protein binding;RNA binding
22826	metabolic process	cytosol;nucleus	protein binding
6120	metabolic process	cytosol	catalytic activity;metal ion binding;protein binding
10457	cell communication;cell differentiation;regulation of biological process;response to stimulus	membrane	protein binding
5048	cell communication;cell differentiation;cell division;cell organization and biogenesis;cell proliferation;cellular component movement;de	chromosome;cytoplasm;cytoskeleton;cytosol;membrane;nucleus	catalytic activity;protein binding
55379		endoplasmic reticulum;membrane;nucleus	protein binding;RNA binding
392	regulation of biological process;response to stimulus;transport	cytoplasm;cytosol;endosome;membrane	enzyme regulator activity;protein binding
10726	cell division;cell organization and biogenesis;cell proliferation;development;metabolic process	cytoplasm;cytoskeleton;cytosol;nucleus	protein binding
5704	metabolic process;regulation of biological process;response to stimulus;transport	cytoplasm;cytosol;membrane;nucleus;proteasome	catalytic activity;nucleotide binding;protein binding;RNA binding
6223	cell differentiation;cell organization and biogenesis;cellular component movement;defense response;metabolic process;regulation of	cytoplasm;cytosol;membrane;nucleus;ribosome	protein binding;RNA binding;structural molecule activity
400668	metabolic process	extracellular;organelle lumen	catalytic activity
5330	cellular component movement;metabolic process;regulation of biological process;response to stimulus;transport	cytoplasm;cytosol;extracellular	catalytic activity;metal ion binding;protein binding;signal transducer activity
353	metabolic process;response to stimulus;transport	cytoplasm;cytosol;extracellular;organelle lumen	catalytic activity;nucleotide binding
10109	cell organization and biogenesis;cellular component movement;regulation of biological process;response to stimulus	cytoplasm;cytoskeleton;cytosol;endosome;membrane	protein binding;structural molecule activity
25932	cell differentiation;cell organization and biogenesis;cellular homeostasis;metabolic process;regulation of biological process;response	cell surface;cytoplasm;cytoskeleton;cytosol;membrane;mitochondrion;nucleus	catalytic activity;protein binding;transporter activity
2289	metabolic process	cytoplasm;cytosol;membrane;nucleus	catalytic activity;protein binding
51602	cell growth;metabolic process	cytosol;membrane;nucleus	protein binding;RNA binding
5861	cell organization and biogenesis;cellular component movement;defense response;metabolic process;regulation of biological process;	cytoplasm;cytosol;endoplasmic reticulum;endosome;Golgi;membrane	catalytic activity;nucleotide binding;protein binding
4046	cellular component movement;defense response;regulation of biological process;response to stimulus	cytoskeleton;membrane	protein binding;signal transducer activity
1786	cell organization and biogenesis;metabolic process;regulation of biological process;response to stimulus	nucleus	catalytic activity;DNA binding;metal ion binding;nucleotide binding;protein binding;RNA binding
128866	cell communication;cell organization and biogenesis;metabolic process;regulation of biological process;response to stimulus;transpor	cytoplasm;cytosol;endosome;membrane;nucleus	protein binding
6749	metabolic process;regulation of biological process;response to stimulus	chromosome;nucleus	DNA binding;protein binding;RNA binding
4288	cell proliferation;metabolic process;regulation of biological process;response to stimulus	chromosome;cytoplasm;membrane;nucleus	DNA binding;nucleotide binding;protein binding;RNA binding
10483	transport	cytoplasm;cytosol;endoplasmic reticulum;membrane	metal ion binding;protein binding
811	cell differentiation;cell organization and biogenesis;cellular homeostasis;metabolic process;regulation of biological process;response	cell surface;cytoplasm;cytosol;endoplasmic reticulum;extracellular;Golgi;membrane;nucleus;organelle lumen	DNA binding;metal ion binding;protein binding;RNA binding
5881	cell communication;cell organization and biogenesis;regulation of biological process;response to stimulus	cytoplasm;cytoskeleton;cytosol;membrane	catalytic activity;nucleotide binding;protein binding
10055	metabolic process;regulation of biological process	cytoplasm;nucleus	catalytic activity;enzyme regulator activity;protein binding
26156	cell differentiation;metabolic process;regulation of biological process	membrane;nucleus	protein binding;RNA binding
4332	cellular component movement;defense response;metabolic process;regulation of biological process;response to stimulus;transport	cytoplasm;extracellular;nucleus;organelle lumen	DNA binding;protein binding;transporter activity
3615	cell organization and biogenesis;cell proliferation;metabolic process;response to stimulus;transport	cytoplasm;cytosol;extracellular;membrane;nucleus;organelle lumen	catalytic activity;DNA binding;metal ion binding;nucleotide binding;protein binding;RNA binding
5885	cell death;cell division;cell organization and biogenesis;metabolic process;regulation of biological process;response to stimulus	chromosome;cytosol;membrane;nucleus	protein binding
16	metabolic process;regulation of biological process	cytoplasm;cytosol;membrane;mitochondrion	catalytic activity;metal ion binding;nucleotide binding;RNA binding
11325	metabolic process;regulation of biological process	cytoplasm;cytosol;membrane;nucleus	catalytic activity;nucleotide binding;protein binding;RNA binding
10598	regulation of biological process;response to stimulus	cytoplasm;cytosol;endoplasmic reticulum	enzyme regulator activity;protein binding
3502	cell organization and biogenesis;defense response;metabolic process;regulation of biological process;response to stimulus	extracellular;membrane	catalytic activity;protein binding
6188	cell death;cell division;cell organization and biogenesis;metabolic process;regulation of biological process;response to stimulus;transp	cytoplasm;cytoskeleton;cytosol;endoplasmic reticulum;membrane;mitochondrion;nucleus;organelle lumen;ribos	catalytic activity;DNA binding;protein binding;RNA binding;structural molecule activity
8417	cell organization and biogenesis;regulation of biological process;transport	endosome;membrane;vacuole	protein binding
6636	cell organization and biogenesis;metabolic process;transport	cytoplasm;cytosol;nucleus;spliceosomal complex	protein binding;RNA binding
2107	cell organization and biogenesis;metabolic process;regulation of biological process	cytoplasm;cytosol;nucleus	protein binding;RNA binding
1854	metabolic process	mitochondrion;nucleus	catalytic activity;metal ion binding;protein binding;RNA binding
10982	cell division;cell proliferation;regulation of biological process;response to stimulus	cytoplasm;cytoskeleton	protein binding
6161	cell organization and biogenesis;metabolic process;response to stimulus;transport	cytosol;membrane;ribosome	RNA binding;structural molecule activity
29766	cell organization and biogenesis;regulation of biological process	cytoplasm;cytoskeleton	protein binding
23385	cell proliferation;metabolic process;regulation of biological process;response to stimulus;transport	endoplasmic reticulum;Golgi;membrane	catalytic activity;protein binding
84817	metabolic process;regulation of biological process;response to stimulus	cytoplasm;cytosol	antioxidant activity;catalytic activity;protein binding
1965	cell communication;metabolic process;regulation of biological process;response to stimulus;transport	cytoplasm;cytosol;membrane;nucleus;ribosome	protein binding;RNA binding
59345	metabolic process;regulation of biological process;response to stimulus	cytosol;membrane	protein binding;signal transducer activity
9550	cellular homeostasis;regulation of biological process;response to stimulus;transport	cytosol;membrane	catalytic activity;protein binding;transporter activity
8789	metabolic process	cytoplasm;cytosol;membrane;nucleus	catalytic activity;metal ion binding;protein binding
55611	metabolic process;regulation of biological process;response to stimulus	cytoplasm;cytosol;nucleus	catalytic activity;nucleotide binding;protein binding
23646	metabolic process	endoplasmic reticulum;membrane	catalytic activity;protein binding
7073	cell death;cell division;defense response;regulation of biological process;response to stimulus	cytoplasm;nucleus;vacuole	DNA binding;RNA binding
10954	cellular homeostasis;metabolic process;regulation of biological process;response to stimulus	endoplasmic reticulum;membrane;organelle lumen	catalytic activity;protein binding
2224	metabolic process;regulation of biological process	cytoplasm;cytosol;mitochondrion	catalytic activity;metal ion binding;RNA binding
1933	metabolic process;response to stimulus	cytoplasm;cytosol;endoplasmic reticulum;nucleus	protein binding;RNA binding
8668	cell organization and biogenesis;metabolic process;regulation of biological process	cytoplasm;cytosol	protein binding;RNA binding
8667	cell organization and biogenesis;metabolic process;regulation of biological process	cytoplasm;cytosol;membrane	protein binding;RNA binding
5049	metabolic process;regulation of biological process;transport	cytoplasm;cytosol;extracellular;membrane;organelle lumen	catalytic activity;protein binding
6222	cell organization and biogenesis;metabolic process;transport	cytoplasm;cytosol;membrane;nucleus;ribosome	protein binding;RNA binding;structural molecule activity
220988	metabolic process;transport	cytoplasm;nucleus;spliceosomal complex	protein binding;RNA binding;transporter activity
5954	metabolic process	endoplasmic reticulum;organelle lumen	metal ion binding;protein binding
2130	metabolic process;regulation of biological process	cytoplasm;membrane;nucleus	metal ion binding;protein binding;RNA binding
22872	cell organization and biogenesis;response to stimulus;transport	cytoplasm;cytosol;endoplasmic reticulum;membrane	protein binding
1653	cell organization and biogenesis;development;metabolic process;regulation of biological process;response to stimulus	cytoplasm;cytosol;membrane;nucleus	catalytic activity;DNA binding;nucleotide binding;protein binding;RNA binding
6037	defense response;metabolic process;response to stimulus;transport	extracellular;organelle lumen	catalytic activity
1639	cell division;cell organization and biogenesis;cellular component movement;metabolic process;regulation of biological process;transp	chromosome;cytoplasm;cytoskeleton;cytosol;membrane;nucleus	catalytic activity;motor activity;protein binding
3538	cell organization and biogenesis;cellular component movement;defense response;metabolic process;regulation of biological process;	extracellular;membrane	catalytic activity;protein binding
			protein binding
2804	cell organization and biogenesis;metabolic process;regulation of biological process;transport	Golgi;membrane	catalytic activity;DNA binding;motor activity;protein binding;RNA binding;transporter activity
8665	metabolic process	cytoplasm;cytosol;membrane	catalytic activity;protein binding;RNA binding
23593	cell organization and biogenesis;regulation of biological process;transport	cytoplasm;extracellular;mitochondrion;organelle lumen	protein binding

231	metabolic process;regulation of biological process;response to stimulus	cytoplasm;cytosol;extracellular;vacuole	catalytic activity;protein binding
10915	metabolic process;regulation of biological process	nucleus	protein binding;RNA binding
191	defense response;metabolic process;response to stimulus;transport	cytoplasm;cytosol;nucleus	catalytic activity;nucleotide binding;protein binding
23549	metabolic process	cytoplasm;cytosol;nucleus	catalytic activity;metal ion binding;protein binding
3837	cell organization and biogenesis;cellular component movement;metabolic process;regulation of biological process;response to stimul	cytoplasm;cytosol;extracellular;membrane;nucleus;organelle lumen	metal ion binding;protein binding;RNA binding;transporter activity
26528	cell differentiation;cell proliferation;development;regulation of biological process	cytoplasm;cytosol;nucleus	protein binding;RNA binding
5860	metabolic process;response to stimulus	cytoplasm;cytosol;mitochondrion	catalytic activity;nucleotide binding;protein binding
8565	cell death;metabolic process;regulation of biological process;response to stimulus	cytoplasm;cytosol	catalytic activity;nucleotide binding;protein binding;RNA binding;signal transducer activity
6102	cell organization and biogenesis;metabolic process;regulation of biological process;transport	cytoplasm;Golgi;membrane	catalytic activity;enzyme regulator activity;nucleotide binding;protein binding
3840	regulation of biological process;response to stimulus;transport	cytoplasm;cytosol;nucleus	protein binding;transporter activity
8498	cell organization and biogenesis;metabolic process;regulation of biological process;transport	cytoplasm;nucleus	enzyme regulator activity;protein binding
6156	cell organization and biogenesis;defense response;metabolic process;response to stimulus;transport	cytosol;membrane;nucleus;ribosome	protein binding;RNA binding;structural molecule activity
6431	cell organization and biogenesis;metabolic process;regulation of biological process;response to stimulus;transport	nucleus	protein binding;RNA binding
9958	cell organization and biogenesis;metabolic process;regulation of biological process;response to stimulus	cytoplasm;cytosol;mitochondrion;nucleus	catalytic activity;protein binding
115207	cell organization and biogenesis;transport	membrane	protein binding;RNA binding;transporter activity
6146	cell organization and biogenesis;metabolic process;transport	cytoplasm;cytosol;nucleus;ribosome	protein binding;RNA binding;structural molecule activity
3123	cell organization and biogenesis;regulation of biological process;response to stimulus	cell surface;endoplasmic reticulum;endosome;Golgi;membrane;vacuole	protein binding
51726	metabolic process;regulation of biological process	cytoplasm;endoplasmic reticulum;membrane;nucleus;organelle lumen	protein binding
654346		cytosol	protein binding
529	cellular homeostasis;regulation of biological process;response to stimulus;transport	cytoplasm;cytosol;endosome;membrane;mitochondrion	catalytic activity;protein binding;transporter activity
6138	cell organization and biogenesis;metabolic process;response to stimulus;transport	cytosol;membrane;nucleus;ribosome	protein binding;RNA binding;structural molecule activity
2495	cellular homeostasis;metabolic process;regulation of biological process;response to stimulus;transport	cytosol;extracellular;nucleus;vacuole	catalytic activity;metal ion binding;protein binding
3301	cellular component movement;metabolic process;regulation of biological process;response to stimulus;transport	cytoplasm;cytoskeleton;cytosol;endoplasmic reticulum;membrane;mitochondrion;nucleus	enzyme regulator activity;metal ion binding;nucleotide binding;protein binding
5591	cell death;cell differentiation;cell organization and biogenesis;cell proliferation;defense response;metabolic process;regulation of biolo	cytosol;membrane;nucleus	catalytic activity;DNA binding;nucleotide binding;protein binding;RNA binding
5547	defense response;metabolic process;regulation of biological process;response to stimulus;transport	membrane;vacuole	catalytic activity;protein binding
3157	metabolic process;regulation of biological process;response to stimulus	cytoplasm;cytosol	catalytic activity;protein binding
10155	cell organization and biogenesis;defense response;metabolic process;regulation of biological process;response to stimulus	chromosome;nucleus	catalytic activity;DNA binding;metal ion binding;protein binding;RNA binding
10212	metabolic process;regulation of biological process;response to stimulus;transport	cytoplasm;membrane;nucleus;spliceosomal complex	catalytic activity;nucleotide binding;protein binding;RNA binding
3093	cell organization and biogenesis;metabolic process;regulation of biological process;response to stimulus	cytoplasm;cytosol;nucleus	catalytic activity;nucleotide binding;protein binding
4144	cell organization and biogenesis;metabolic process	cytosol	catalytic activity;metal ion binding;nucleotide binding;protein binding
6432	metabolic process;regulation of biological process;transport	cytoplasm;nucleus	metal ion binding;protein binding;RNA binding
10093	cell organization and biogenesis;regulation of biological process;response to stimulus	cytoplasm;cytoskeleton;cytosol	protein binding;structural molecule activity
8226	metabolic process	cytoplasm;cytosol	catalytic activity;metal ion binding
2771	cell division;cell proliferation;metabolic process;regulation of biological process;response to stimulus	cytoplasm;cytoskeleton;cytosol;membrane;nucleus	catalytic activity;metal ion binding;nucleotide binding;protein binding;signal transducer activity
51074	cell death;cell organization and biogenesis;metabolic process;regulation of biological process	cytoplasm	catalytic activity;metal ion binding;protein binding
6208	cell differentiation;cell organization and biogenesis;metabolic process;regulation of biological process;transport	cytosol;membrane;mitochondrion;ribosome	protein binding;RNA binding;structural molecule activity;translation regulator activity
5813	cell differentiation;metabolic process;regulation of biological process	cytoplasm;nucleus	DNA binding;protein binding;RNA binding;translation regulator activity
4924	metabolic process;regulation of biological process;response to stimulus	cytoplasm;endoplasmic reticulum;endosome;extracellular;Golgi;membrane;nucleus;organelle lumen	DNA binding;metal ion binding;protein binding
6633	cell organization and biogenesis;metabolic process;transport	cytoplasm;cytosol;nucleus;spliceosomal complex	protein binding;RNA binding
26135	regulation of biological process	cytoplasm;cytosol;membrane;nucleus	protein binding;RNA binding
6152	cell organization and biogenesis;cellular component movement;metabolic process;regulation of biological process;transport	cytoplasm;cytosol;membrane;ribosome	protein binding;RNA binding;structural molecule activity
9938	cell organization and biogenesis;metabolic process;regulation of biological process;response to stimulus	cytosol	enzyme regulator activity
2314	cell organization and biogenesis;development;metabolic process;regulation of biological process	cytoplasm;cytoskeleton;cytosol;nucleus	protein binding
3119	regulation of biological process;response to stimulus	endoplasmic reticulum;endosome;Golgi;membrane;vacuole	receptor activity;signal transducer activity
4522	metabolic process	cytoplasm;cytosol;membrane;mitochondrion	catalytic activity;nucleotide binding;protein binding
84932	regulation of biological process;response to stimulus;transport	endoplasmic reticulum;Golgi;membrane	catalytic activity;nucleotide binding;protein binding
372	response to stimulus;transport	cytoplasm;cytosol;endoplasmic reticulum;Golgi;membrane	RNA binding
26578	regulation of biological process;response to stimulus;transport	cytoplasm;extracellular;organelle lumen	protein binding
8669	cell organization and biogenesis;metabolic process;regulation of biological process	cytoplasm;cytosol	protein binding;RNA binding
1371	metabolic process;response to stimulus	cytosol;membrane;mitochondrion	catalytic activity;protein binding;structural molecule activity
7174	metabolic process	cytoplasm;cytosol;nucleus	catalytic activity;protein binding
84844	metabolic process;regulation of biological process	nucleus;spliceosomal complex	DNA binding;metal ion binding;RNA binding
3336	cell differentiation;metabolic process;regulation of biological process;response to stimulus	cytoplasm;membrane;mitochondrion;organelle lumen	metal ion binding;nucleotide binding;protein binding;RNA binding
10728	cell organization and biogenesis;metabolic process;regulation of biological process;response to stimulus	cytoplasm;cytosol;nucleus	catalytic activity;protein binding
1399	metabolic process;regulation of biological process;response to stimulus	cytoplasm;cytosol;endosome	protein binding;RNA binding;signal transducer activity
10226	transport	cytoplasm;cytosol;endosome;Golgi;membrane	protein binding
5708	metabolic process;regulation of biological process;response to stimulus;transport	cytoplasm;cytosol;extracellular;membrane;nucleus;organelle lumen;proteasome	catalytic activity;enzyme regulator activity;nucleotide binding;protein binding
6128	cell organization and biogenesis;metabolic process;regulation of biological process;transport	cytosol;membrane;nucleus;ribosome	DNA binding;protein binding;RNA binding;structural molecule activity
6635	cell organization and biogenesis;metabolic process;transport	cytoplasm;cytosol;nucleus;spliceosomal complex	protein binding;RNA binding
27243	cell communication;cell organization and biogenesis;metabolic process;regulation of biological process;response to stimulus;transpor	chromosome;cytosol;endosome;membrane	protein binding
54440	regulation of biological process	cytoplasm;nucleus	protein binding
3925	cell differentiation;cell organization and biogenesis;development;regulation of biological process;response to stimulus	cytoplasm;cytoskeleton;cytosol;membrane	protein binding;signal transducer activity
7114	cell differentiation;cell organization and biogenesis;metabolic process;regulation of biological process;response to stimulus;transport	cytoplasm;cytoskeleton;cytosol;extracellular;membrane;nucleus;organelle lumen	catalytic activity;metal ion binding;nucleotide binding;protein binding;RNA binding;structural molec
682	cell organization and biogenesis;cellular component movement;metabolic process;regulation of biological process;response to stimul	membrane;mitochondrion	protein binding;transporter activity
23499	cell organization and biogenesis;regulation of biological process;response to stimulus;transport	cytoplasm;cytoskeleton;Golgi;membrane	catalytic activity;metal ion binding;motor activity;protein binding;RNA binding
1476	regulation of biological process;response to stimulus;transport	cytoplasm;cytosol;extracellular;nucleus;organelle lumen	enzyme regulator activity;protein binding;RNA binding
9276	transport	cytoplasm;cytosol;Golgi;membrane	protein binding;structural molecule activity
25801	cell organization and biogenesis;metabolic process;transport	cytoplasm;cytosol;extracellular;membrane;organelle lumen	catalytic activity;metal ion binding;protein binding
5196	cellular component movement;defense response;regulation of biological process;response to stimulus;transport	cytoplasm;extracellular;organelle lumen	protein binding
1994	development;metabolic process;regulation of biological process	cytoplasm;cytosol;membrane;nucleus	protein binding;RNA binding
7323	cell death;metabolic process;regulation of biological process;response to stimulus	cytosol;endosome;membrane	catalytic activity;nucleotide binding;protein binding
1743	metabolic process	membrane;mitochondrion;nucleus;organelle lumen	catalytic activity
6449	metabolic process;regulation of biological process	cytoplasm;cytosol;membrane;nucleus	protein binding
1088	cellular component movement;response to stimulus;transport	membrane	protein binding
2029	cell division;regulation of biological process;response to stimulus;transport	cytoplasm	enzyme regulator activity;protein binding
7072	cell death;regulation of biological process;response to stimulus	cytoplasm;cytosol;nucleus	protein binding;RNA binding
3383	cell communication;cell organization and biogenesis;cellular component movement;cellular homeostasis;defense response;regulation	cell surface;membrane	protein binding;receptor activity;signal transducer activity
216	metabolic process;regulation of biological process	cytoplasm;cytosol	catalytic activity;enzyme regulator activity;nucleotide binding
5912	cell organization and biogenesis;regulation of biological process;response to stimulus;transport	cytosol;endosome;membrane	catalytic activity;nucleotide binding;protein binding
54205	cell death;cell organization and biogenesis;metabolic process;regulation of biological process;response to stimulus;transport	cytosol;membrane;mitochondrion;nucleus	catalytic activity;metal ion binding;protein binding
388	cell death;cell differentiation;development;regulation of biological process;response to stimulus;transport	cytosol;endosome;membrane;nucleus	catalytic activity;nucleotide binding;protein binding
2990	metabolic process;transport	extracellular;membrane;organelle lumen;vacuole	catalytic activity;protein binding
7336	cell proliferation;metabolic process;regulation of biological process;response to stimulus	cytoplasm;nucleus	catalytic activity;protein binding
3385	cell organization and biogenesis;regulation of biological process;response to stimulus;transport	membrane	protein binding
8607	cell division;cell organization and biogenesis;metabolic process;regulation of biological process;response to stimulus	cytoplasm;cytoskeleton;cytosol;membrane;nucleus	catalytic activity;nucleotide binding;protein binding
199	cell communication;cell organization and biogenesis;defense response;regulation of biological process;response to stimulus	cytoplasm;cytoskeleton;cytosol;membrane;nucleus	metal ion binding;protein binding
6187	cell organization and biogenesis;metabolic process;regulation of biological process;transport	cytosol;membrane;nucleus;ribosome	protein binding;RNA binding;structural molecule activity
10394	metabolic process;regulation of biological process;response to stimulus;transport	extracellular;organelle lumen	
11171	cell organization and biogenesis;metabolic process;regulation of biological process	cytoplasm;cytosol;nucleus	protein binding;RNA binding
6427	metabolic process;regulation of biological process;response to stimulus;transport	cytosol;nucleus;spliceosomal complex	protein binding;RNA binding
51382	cell organization and biogenesis;cellular homeostasis;regulation of biological process;response to stimulus;transport	cytosol;membrane	catalytic activity;protein binding;transporter activity
3895	cellular component movement;metabolic process	endoplasmic reticulum;membrane;organelle lumen	protein binding;RNA binding
5935	metabolic process;regulation of biological process	cytoplasm;nucleus	protein binding;RNA binding
6118	cell organization and biogenesis;cellular homeostasis;metabolic process;regulation of biological process;response to stimulus	chromosome;nucleus	DNA binding;protein binding
1968	metabolic process;transport	cytoplasm;cytosol;nucleus	catalytic activity;nucleotide binding;protein binding;RNA binding
2287	metabolic process	cytoplasm;nucleus	catalytic activity;protein binding;receptor activity;RNA binding
10197	cell death;metabolic process;regulation of biological process;response to stimulus;transport	cytoplasm;cytosol;membrane;nucleus;proteasome	enzyme regulator activity;protein binding
2079	metabolic process;regulation of biological process		DNA binding;nucleotide binding;protein binding;RNA binding
8241	metabolic process;regulation of biological process	nucleus	metal ion binding;protein binding;RNA binding
6117	cell organization and biogenesis;cellular homeostasis;metabolic process;regulation of biological process;response to stimulus	nucleus	DNA binding;metal ion binding;protein binding
57175	cell organization and biogenesis;cellular component movement;regulation of biological process;response to stimulus	cytoplasm;cytoskeleton;cytosol;membrane	protein binding

9527	cell organization and biogenesis;regulation of biological process;transport	cytosol;Golgi;membrane	protein binding
1340	metabolic process;transport	membrane;mitochondrion	catalytic activity;transporter activity
9459	cell death;cell organization and biogenesis;metabolic process;regulation of biological process;response to stimulus	cytosol	enzyme regulator activity;protein binding
10135	cell communication;metabolic process;regulation of biological process;response to stimulus	cytoplasm;cytosol;extracellular;nucleus	catalytic activity;protein binding
5111	cell differentiation;cell organization and biogenesis;cell proliferation;cellular homeostasis;metabolic process;regulation of biological pr	nucleus	catalytic activity;DNA binding;enzyme regulator activity;protein binding
92675	metabolic process;regulation of biological process	cytoplasm;cytosol;nucleus	catalytic activity;DNA binding;metal ion binding;RNA binding
3692	cell organization and biogenesis;metabolic process;regulation of biological process;response to stimulus;transport	cytoplasm;cytosol;nucleus	protein binding;RNA binding
960	cell organization and biogenesis;cellular component movement;metabolic process;regulation of biological process;response to stimul	cell surface;cytosol;Golgi;membrane	protein binding;receptor activity;signal transducer activity
51596	response to stimulus	membrane	metal ion binding;protein binding
9092	cell organization and biogenesis;metabolic process;regulation of biological process;response to stimulus	cytoplasm;cytosol;Golgi;nucleus;spliceosomal complex	protein binding;RNA binding
4835	metabolic process;regulation of biological process;response to stimulus	cytoplasm;cytosol	catalytic activity;metal ion binding;nucleotide binding;protein binding
5576	coagulation;metabolic process;regulation of biological process;response to stimulus	cytoplasm;cytosol;membrane	catalytic activity;enzyme regulator activity;nucleotide binding;protein binding
30001	cell death;cell differentiation;cell organization and biogenesis;cellular homeostasis;metabolic process;regulation of biological process	endoplasmic reticulum;membrane;organelle lumen	catalytic activity;protein binding
5723	metabolic process;response to stimulus	cytoplasm;cytosol	catalytic activity;metal ion binding;protein binding
6184	metabolic process	cytosol;endoplasmic reticulum;membrane	catalytic activity;protein binding;RNA binding
3109	cell organization and biogenesis;regulation of biological process;response to stimulus	membrane	protein binding
23234	regulation of biological process;response to stimulus	cytoplasm;cytosol;membrane;nucleus	protein binding
51690	metabolic process	cytosol;nucleus;spliceosomal complex	protein binding;RNA binding
131076		mitochondrion	
10015	cell death;cell division;cell organization and biogenesis;regulation of biological process;transport	cytoplasm;cytoskeleton;cytosol;extracellular;membrane	protein binding
2773	cell division;cell organization and biogenesis;metabolic process;regulation of biological process;response to stimulus;transport	cytoplasm;cytoskeleton;Golgi;membrane;nucleus	catalytic activity;metal ion binding;nucleotide binding;protein binding;signal transducer activity
3028	cell organization and biogenesis;metabolic process	cytoplasm;membrane;mitochondrion;organelle lumen	catalytic activity;protein binding;RNA binding
90952	cellular component movement	membrane	protein binding
6206	cell organization and biogenesis;metabolic process;transport	cytoplasm;cytosol;membrane;ribosome	RNA binding;structural molecule activity
670	metabolic process;response to stimulus	membrane;mitochondrion	catalytic activity
689	metabolic process;regulation of biological process;transport	cytoplasm;cytosol;nucleus	protein binding;RNA binding
1655	cell differentiation;cell growth;cell organization and biogenesis;metabolic process;regulation of biological process;response to stimulu	cytoplasm;membrane;nucleus;spliceosomal complex	catalytic activity;nucleotide binding;protein binding;RNA binding
6342	cell organization and biogenesis;metabolic process;regulation of biological process;transport	cytoplasm;membrane;mitochondrion;organelle lumen	catalytic activity;protein binding;transporter activity
84836	metabolic process;regulation of biological process	cytoplasm;cytosol;nucleus	catalytic activity;protein binding
10294	metabolic process;regulation of biological process;response to stimulus	cytosol;membrane	enzyme regulator activity;metal ion binding;nucleotide binding;protein binding
10291	cell organization and biogenesis;metabolic process;regulation of biological process	nucleus;spliceosomal complex	protein binding;RNA binding
9774	cell death;metabolic process;regulation of biological process;response to stimulus	cytoplasm;nucleus	DNA binding;protein binding;RNA binding
6191	cell organization and biogenesis;development;metabolic process;regulation of biological process;transport	cytoplasm;cytosol;membrane;ribosome	protein binding;RNA binding;structural molecule activity
9050	cell organization and biogenesis;cellular component movement	cytoplasm;cytoskeleton;cytosol;membrane	catalytic activity;motor activity;protein binding
4035	cell proliferation;development;metabolic process;regulation of biological process;response to stimulus;transport	cytoplasm;cytosol;endosome;membrane;nucleus	metal ion binding;protein binding;receptor activity;RNA binding;transporter activity
5295	cell death;cell differentiation;cellular component movement;cellular homeostasis;defense response;metabolic process;regulation of bi	cytoplasm;cytosol;membrane;nucleus	catalytic activity;enzyme regulator activity;nucleotide binding;protein binding
84717	regulation of biological process	nucleus	protein binding
6230	cell organization and biogenesis;metabolic process;transport	cytosol;nucleus;ribosome	catalytic activity;nucleotide binding;protein binding;RNA binding;structural molecule activity
6403	cellular component movement;defense response;regulation of biological process;response to stimulus;transport	membrane	protein binding
7265	metabolic process	cytosol;membrane	protein binding
6277	cell organization and biogenesis;regulation of biological process;response to stimulus;transport	cytoplasm;cytosol;extracellular;membrane;nucleus	metal ion binding;protein binding;transporter activity
2969	cellular homeostasis;metabolic process;regulation of biological process;response to stimulus	cytoplasm;membrane;nucleus	DNA binding;protein binding
5869	cell organization and biogenesis;regulation of biological process;response to stimulus;transport	endosome;membrane	catalytic activity;nucleotide binding;protein binding
27258	cell organization and biogenesis;metabolic process	cytosol;nucleus;spliceosomal complex	protein binding;RNA binding
5705	metabolic process;regulation of biological process;response to stimulus;transport	cytoplasm;cytosol;membrane;nucleus;proteasome	catalytic activity;nucleotide binding;protein binding;RNA binding
7419	cell communication;defense response;regulation of biological process;response to stimulus;transport	membrane;mitochondrion;nucleus	nucleotide binding;transporter activity
116496	regulation of biological process;response to stimulus	cytoplasm;cytosol;membrane	protein binding
3978	cell division;metabolic process;response to stimulus	mitochondrion;nucleus	catalytic activity;DNA binding;metal ion binding;nucleotide binding
51719	cell organization and biogenesis;metabolic process;regulation of biological process;response to stimulus;transport	cytoplasm;cytosol;extracellular;organelle lumen	catalytic activity;enzyme regulator activity;protein binding
10204	cell organization and biogenesis;regulation of biological process;transport	cytoplasm;cytosol;membrane;nucleus	protein binding;transporter activity
5531	metabolic process;regulation of biological process;response to stimulus	cytoplasm;cytoskeleton;cytosol;membrane;nucleus	catalytic activity;metal ion binding;protein binding;signal transducer activity
10657	cell organization and biogenesis;cell proliferation;metabolic process;regulation of biological process;response to stimulus	cytoplasm;cytosol;membrane;nucleus	DNA binding;protein binding;RNA binding
6727	cell organization and biogenesis;response to stimulus;transport	cytoplasm;cytosol;extracellular;nucleus;organelle lumen	protein binding;RNA binding
30011	cell communication;cell death;cell organization and biogenesis;cellular component movement;metabolic process;regulation of biologi	cytoplasm;cytoskeleton;cytosol;membrane	catalytic activity;protein binding
51138	cell organization and biogenesis;metabolic process;response to stimulus	cytoplasm;cytosol;nucleus	catalytic activity;protein binding
134510	metabolic process	nucleus	catalytic activity;protein binding
306	defense response;regulation of biological process;response to stimulus;transport	cytoplasm;cytosol;membrane	enzyme regulator activity;metal ion binding;protein binding
545	development;metabolic process;regulation of biological process;response to stimulus	chromosome;Golgi;nucleus	catalytic activity;DNA binding;nucleotide binding;protein binding
1459	cell communication;cell death;metabolic process;regulation of biological process;response to stimulus	chromosome;cytoplasm;cytosol;membrane;nucleus	catalytic activity;nucleotide binding;protein binding
51691	metabolic process	nucleus;spliceosomal complex	protein binding;RNA binding
3949	cell organization and biogenesis;metabolic process;regulation of biological process;response to stimulus;transport	cell surface;endosome;Golgi;membrane;vacuole	metal ion binding;protein binding;receptor activity
2280	cell organization and biogenesis;cell proliferation;cellular homeostasis;metabolic process;regulation of biological process;response to	cytoplasm;cytosol;endoplasmic reticulum;membrane	catalytic activity;protein binding;signal transducer activity
8402	metabolic process;transport	membrane;mitochondrion;nucleus	RNA binding;transporter activity
22913	metabolic process;regulation of biological process	nucleus;spliceosomal complex	protein binding;RNA binding
8942	metabolic process;response to stimulus	cytoplasm;cytosol;mitochondrion	catalytic activity;protein binding
350	metabolic process;regulation of biological process;response to stimulus;transport	cell surface;extracellular;organelle lumen	enzyme regulator activity;protein binding
9554	cell organization and biogenesis;regulation of biological process;transport	endoplasmic reticulum;Golgi;membrane	protein binding
873; 54093	cell differentiation;metabolic process	cytoplasm;cytosol	catalytic activity
54749		extracellular;vacuole	metal ion binding
4074	cell organization and biogenesis;regulation of biological process;response to stimulus;transport	endosome;membrane;vacuole	protein binding;receptor activity;signal transducer activity;transporter activity
3676	cell differentiation;cell organization and biogenesis;cellular component movement;regulation of biological process;response to stimulu	cell surface;membrane	metal ion binding;protein binding;receptor activity;signal transducer activity
3263	cellular homeostasis;metabolic process;regulation of biological process;transport	extracellular;organelle lumen	metal ion binding;protein binding;transporter activity
8878	cell communication;cell death;cell differentiation;cell organization and biogenesis;metabolic process;regulation of biological process;r	cytoplasm;cytosol;endoplasmic reticulum;endosome;nucleus;vacuole	catalytic activity;metal ion binding;protein binding
1615	cell organization and biogenesis;metabolic process	cytoplasm;cytosol;membrane	catalytic activity;nucleotide binding;protein binding;RNA binding
9689	metabolic process;regulation of biological process	cytoplasm;membrane	protein binding;RNA binding
10987	cell organization and biogenesis;metabolic process;regulation of biological process;response to stimulus;transport	chromosome;cytoplasm;cytosol;nucleus	catalytic activity;metal ion binding;protein binding;RNA binding
1665	metabolic process;response to stimulus	cytoplasm;nucleus;spliceosomal complex	catalytic activity;nucleotide binding;protein binding;RNA binding
2802	metabolic process;regulation of biological process;transport	cytoplasm;cytosol;Golgi;membrane;nucleus	catalytic activity;motor activity;protein binding;structural molecule activity;transporter activity
5236	metabolic process;transport	cytoplasm;cytoskeleton;cytosol;extracellular	catalytic activity;metal ion binding;protein binding
6627	metabolic process;regulation of biological process;response to stimulus	nucleus;spliceosomal complex	protein binding;RNA binding
51060	cell differentiation;cellular homeostasis;metabolic process;regulation of biological process	endoplasmic reticulum;organelle lumen	catalytic activity;protein binding
4676	cell organization and biogenesis	cytoplasm;nucleus	protein binding;RNA binding
9045	cell organization and biogenesis;metabolic process;transport	cytosol;membrane;ribosome	protein binding;RNA binding;structural molecule activity
25996	metabolic process	cytoplasm;mitochondrion;nucleus;organelle lumen	catalytic activity
3065	cell differentiation;cell organization and biogenesis;coagulation;metabolic process;regulation of biological process	chromosome;cytoplasm;cytosol;nucleus	catalytic activity;DNA binding;metal ion binding;protein binding
29979	cell communication;cell organization and biogenesis;metabolic process;regulation of biological process;response to stimulus	cytoplasm;cytosol;endoplasmic reticulum;membrane;nucleus;proteasome;vacuole	protein binding
11252	cell organization and biogenesis;regulation of biological process;transport	cytoplasm;cytoskeleton;cytosol;endosome;membrane	protein binding;transporter activity
55646		nucleus	metal ion binding;protein binding;RNA binding
5202	metabolic process;regulation of biological process	cytoplasm;cytosol;mitochondrion;nucleus	protein binding
11168	cell organization and biogenesis;metabolic process;regulation of biological process;response to stimulus;transport	cytosol;nucleus	DNA binding;metal ion binding;protein binding;RNA binding
23075	cell organization and biogenesis;cellular homeostasis;metabolic process;regulation of biological process	cytoplasm;cytoskeleton;membrane;nucleus	DNA binding;metal ion binding;nucleotide binding;protein binding
51493	metabolic process	cytoplasm;cytosol;membrane;nucleus	catalytic activity;metal ion binding;nucleotide binding;protein binding;RNA binding
3043	cell organization and biogenesis;coagulation;metabolic process;regulation of biological process;response to stimulus;transport	cytosol;extracellular;organelle lumen	antioxidant activity;catalytic activity;metal ion binding;protein binding;transporter activity
58513	cell differentiation;cell organization and biogenesis;cellular component movement;metabolic process;regulation of biological process;	cytoplasm;cytoskeleton;cytosol;Golgi;membrane;nucleus	catalytic activity;metal ion binding;motor activity;nucleotide binding;protein binding;structural molec
10094	cell organization and biogenesis;cellular component movement;regulation of biological process;response to stimulus	cytoplasm;cytoskeleton;cytosol;membrane	protein binding;structural molecule activity
2618	metabolic process;response to stimulus	cytoplasm;cytosol	catalytic activity;metal ion binding;nucleotide binding
10146	metabolic process;regulation of biological process;response to stimulus;transport	cytoplasm;cytosol;membrane;nucleus	catalytic activity;DNA binding;nucleotide binding;protein binding;RNA binding
1066	cell differentiation;metabolic process;response to stimulus	cytosol;endoplasmic reticulum;organelle lumen	catalytic activity
27306	metabolic process;regulation of biological process;response to stimulus	cytoplasm;cytosol	catalytic activity;metal ion binding;protein binding
3107	regulation of biological process;response to stimulus	cell surface;endoplasmic reticulum;Golgi;membrane	protein binding
80150	metabolic process	cytoplasm;cytosol;nucleus	catalytic activity

10541	cell organization and biogenesis;metabolic process;regulation of biological process	cytoplasm;nucleus	protein binding
51154	cell organization and biogenesis;metabolic process	cytoplasm;nucleus	protein binding;RNA binding
9138	cell proliferation;regulation of biological process;response to stimulus	cytoplasm;cytosol;membrane	enzyme regulator activity;protein binding;RNA binding
3113	regulation of biological process;response to stimulus	cell surface;endoplasmic reticulum;endosome;Golgi;membrane;vacuole	receptor activity;signal transducer activity
8520	cell organization and biogenesis;metabolic process;regulation of biological process;response to stimulus	cytoplasm;nucleus	catalytic activity;protein binding
9943	metabolic process;regulation of biological process;response to stimulus	cytoplasm;cytosol	catalytic activity;metal ion binding;nucleotide binding;protein binding;signal transducer activity
5859	metabolic process;regulation of biological process	cytoplasm;cytosol;organelle lumen	catalytic activity;enzyme regulator activity;nucleotide binding;protein binding
10694	cell organization and biogenesis;metabolic process;regulation of biological process;transport	cytoplasm;cytoskeleton;cytosol;extracellular;organelle lumen	catalytic activity;nucleotide binding;protein binding
23524	metabolic process	nucleus;spliceosomal complex	nucleotide binding;protein binding;RNA binding
5702	metabolic process;regulation of biological process;response to stimulus;transport	cytoplasm;cytosol;extracellular;membrane;nucleus;organelle lumen;proteasome	catalytic activity;enzyme regulator activity;nucleotide binding;protein binding
1654	cell death;cell organization and biogenesis;defense response;metabolic process;regulation of biological process;response to stimulus	cytoplasm;cytosol;extracellular;membrane;mitochondrion;nucleus;organelle lumen	catalytic activity;DNA binding;enzyme regulator activity;nucleotide binding;protein binding;RNA bin
3921	cell organization and biogenesis;metabolic process;transport	cytoplasm;cytosol;membrane;nucleus;ribosome	protein binding;receptor activity;RNA binding;structural molecule activity
5770	cell organization and biogenesis;metabolic process;regulation of biological process;response to stimulus	cytoplasm;cytosol;endoplasmic reticulum;endosome;membrane	catalytic activity;metal ion binding;protein binding;RNA binding
8467	cell organization and biogenesis;metabolic process;regulation of biological process	chromosome;nucleus	catalytic activity;DNA binding;nucleotide binding;protein binding
9601	cellular homeostasis;metabolic process;regulation of biological process;response to stimulus;transport	cell surface;endoplasmic reticulum;organelle lumen	catalytic activity;metal ion binding;protein binding;RNA binding
3315	cellular component movement;metabolic process;regulation of biological process;response to stimulus	cytoplasm;cytoskeleton;cytosol;membrane;nucleus;proteasome	enzyme regulator activity;protein binding;RNA binding
2782	cell death;cell organization and biogenesis;cell proliferation;cellular homeostasis;metabolic process;regulation of biological process;re	cytosol;membrane	catalytic activity;protein binding;signal transducer activity
4706	cell organization and biogenesis;metabolic process;transport	cytosol;membrane;mitochondrion;organelle lumen	catalytic activity;metal ion binding;protein binding;transporter activity
23450	cell organization and biogenesis;metabolic process;regulation of biological process	nucleus;spliceosomal complex	protein binding;RNA binding
50848	cell differentiation;cell organization and biogenesis;cellular component movement;defense response;regulation of biological process;r	membrane	protein binding;receptor activity
29941	cellular component movement;metabolic process;regulation of biological process;response to stimulus	cytoplasm;Golgi;nucleus	catalytic activity;nucleotide binding;protein binding
2992	metabolic process;transport	cytosol;extracellular;membrane;organelle lumen	catalytic activity;metal ion binding;protein binding
1478	metabolic process	nucleus	protein binding;RNA binding
5701	cell differentiation;metabolic process;regulation of biological process;response to stimulus;transport	cytoplasm;cytosol;extracellular;membrane;nucleus;organelle lumen;proteasome	catalytic activity;nucleotide binding;protein binding
5007	metabolic process;regulation of biological process;transport	cytoplasm;cytosol;endoplasmic reticulum;Golgi;membrane;nucleus	protein binding;transporter activity
6734	cell organization and biogenesis;transport	endoplasmic reticulum;membrane	catalytic activity;nucleotide binding;RNA binding
6742	cell organization and biogenesis;metabolic process;regulation of biological process	mitochondrion;nucleus;organelle lumen	DNA binding;protein binding;RNA binding
4282	cell organization and biogenesis;cell proliferation;cellular component movement;defense response;metabolic process;regulation of bi	cell surface;cytoplasm;cytosol;extracellular;organelle lumen	catalytic activity;protein binding
10856	cell organization and biogenesis;metabolic process;regulation of biological process;response to stimulus	cytoplasm;cytosol;membrane;nucleus	catalytic activity;DNA binding;nucleotide binding;protein binding
644	metabolic process	cytoplasm;cytosol	catalytic activity;metal ion binding;protein binding
58478	metabolic process	cytoplasm;cytosol;nucleus	catalytic activity;metal ion binding
1845	metabolic process;regulation of biological process	cytosol;nucleus	catalytic activity;protein binding
7345	cell communication;cell organization and biogenesis;cell proliferation;cellular component movement;metabolic process;regulation of b	cytoplasm;cytosol;endoplasmic reticulum;membrane	catalytic activity;protein binding
10250	metabolic process;transport	cytosol;nucleus;spliceosomal complex	DNA binding;protein binding;RNA binding
4849	cell differentiation;development;metabolic process;regulation of biological process;response to stimulus	cytoplasm;cytosol;nucleus	protein binding
8904	cell differentiation;cell growth;metabolic process;regulation of biological process;response to stimulus;transport	cytoplasm;cytosol;membrane;nucleus	catalytic activity;metal ion binding;protein binding;transporter activity
10053	regulation of biological process;transport	cytosol;Golgi;membrane	protein binding
4673	cell organization and biogenesis;metabolic process;regulation of biological process	membrane;nucleus	protein binding;RNA binding
57095	response to stimulus	nucleus	
8574	metabolic process	cytoplasm;cytosol;Golgi	catalytic activity;protein binding
1429	cell organization and biogenesis;metabolic process	cytoplasm;cytosol	catalytic activity;metal ion binding;nucleotide binding;RNA binding
6599	cell organization and biogenesis;metabolic process;regulation of biological process;response to stimulus	chromosome;cytoplasm;nucleus	DNA binding;protein binding
8766	cell organization and biogenesis;metabolic process;regulation of biological process;response to stimulus;transport	cytoplasm;cytosol;endosome;Golgi;membrane;mitochondrion	catalytic activity;nucleotide binding;protein binding
1611	cell communication;cell death;metabolic process;regulation of biological process;response to stimulus		protein binding
3501	cell organization and biogenesis;defense response;metabolic process;regulation of biological process;response to stimulus	extracellular;membrane	catalytic activity;protein binding
10450	metabolic process;regulation of biological process;response to stimulus;transport	cytosol;extracellular;nucleus;organelle lumen;spliceosomal complex	catalytic activity;protein binding;RNA binding
10380	metabolic process	cytosol	catalytic activity;metal ion binding
5694	metabolic process;regulation of biological process;response to stimulus;transport	cytoplasm;cytosol;nucleus;proteasome	catalytic activity;protein binding
10209	metabolic process;regulation of biological process;response to stimulus	cytoplasm;nucleus	protein binding;RNA binding
1460	cell communication;cell organization and biogenesis;metabolic process;regulation of biological process;response to stimulus;transpor	chromosome;cytoplasm;cytosol;extracellular;membrane;nucleus;organelle lumen	catalytic activity;enzyme regulator activity;metal ion binding;protein binding
7177; 64499	cell organization and biogenesis;defense response;metabolic process;response to stimulus	extracellular	catalytic activity;protein binding
25804	cell organization and biogenesis;metabolic process	cytosol;membrane;nucleus;spliceosomal complex	protein binding;RNA binding
55911	metabolic process;transport	membrane	receptor activity
3208	regulation of biological process;response to stimulus	cytoplasm;cytosol;membrane	metal ion binding;protein binding
10054	metabolic process;regulation of biological process	cytoplasm;nucleus	catalytic activity;enzyme regulator activity;metal ion binding;nucleotide binding;protein binding
81027	cell organization and biogenesis	cytoplasm;cytoskeleton	catalytic activity;nucleotide binding;structural molecule activity
2752	cell communication;cell organization and biogenesis;cell proliferation;metabolic process;regulation of biological process;response to s	cytoplasm;cytosol;endoplasmic reticulum;mitochondrion;nucleus	catalytic activity;metal ion binding;nucleotide binding;protein binding
1650	metabolic process;response to stimulus;transport	endoplasmic reticulum;membrane	catalytic activity;protein binding
9141	cell death;regulation of biological process;response to stimulus	cytoplasm;cytosol;nucleus	DNA binding;enzyme regulator activity;protein binding
495	transport	membrane	catalytic activity;metal ion binding;nucleotide binding;transporter activity
6194	cell death;cell differentiation;cell organization and biogenesis;cell proliferation;metabolic process;regulation of biological process;resp	cytoplasm;cytosol;membrane;nucleus;ribosome	protein binding;RNA binding;structural molecule activity
6286	cellular component movement;response to stimulus;transport	cytoplasm;extracellular;membrane;nucleus;organelle lumen	metal ion binding;protein binding
6726; 653226	cell organization and biogenesis;regulation of biological process;transport	cytoplasm;cytosol	protein binding;RNA binding
55707	transport	membrane	
7064	metabolic process	cytoplasm;cytosol	catalytic activity;metal ion binding
7319	cell organization and biogenesis;metabolic process;regulation of biological process;response to stimulus	chromosome;cytoplasm;cytosol	catalytic activity;nucleotide binding;protein binding
152007	regulation of biological process	extracellular;Golgi;membrane	protein binding
10480	metabolic process	cytoplasm;cytosol	protein binding;RNA binding
133619		Golgi	protein binding
7165	regulation of biological process	cytoplasm	protein binding;RNA binding
10051	cell division;cell organization and biogenesis;metabolic process	chromosome;cytoplasm;cytosol;nucleus	nucleotide binding;protein binding
972	cell organization and biogenesis;cell proliferation;cellular component movement;defense response;metabolic process;regulation of bi	cell surface;endoplasmic reticulum;endosome;Golgi;membrane;organelle lumen;vacuole	protein binding;receptor activity;signal transducer activity
57486	metabolic process;regulation of biological process	cytoplasm;extracellular;membrane;mitochondrion	catalytic activity;metal ion binding
6229	cell organization and biogenesis;metabolic process;transport	cytosol;membrane;nucleus;ribosome	protein binding;RNA binding;structural molecule activity
7812	metabolic process;regulation of biological process	cytoplasm;cytosol;Golgi;membrane	DNA binding;protein binding;RNA binding
8943	cell organization and biogenesis;cellular component movement;metabolic process;regulation of biological process;transport	cytoplasm;Golgi;membrane	transporter activity
307	cell differentiation;regulation of biological process;response to stimulus	cell surface;cytoplasm;membrane;nucleus	enzyme regulator activity;metal ion binding;protein binding
399687	cell differentiation;cell organization and biogenesis;cellular component movement;metabolic process;regulation of biological process;	cell surface;cytoplasm;cytoskeleton;Golgi;membrane	catalytic activity;DNA binding;motor activity;nucleotide binding;protein binding;RNA binding;structu
27339	cell organization and biogenesis;cell proliferation;metabolic process;regulation of biological process;response to stimulus	cytoplasm;cytoskeleton;membrane;nucleus;spliceosomal complex	catalytic activity;metal ion binding;protein binding
1656	cell organization and biogenesis;metabolic process;regulation of biological process	cytoplasm;cytosol;membrane;mitochondrion;nucleus	catalytic activity;nucleotide binding;protein binding;RNA binding
10946	cell organization and biogenesis;metabolic process	nucleus;spliceosomal complex	metal ion binding;protein binding;RNA binding
2734	cellular component movement;regulation of biological process	Golgi;membrane	protein binding
7153	cell differentiation;cell division;cell organization and biogenesis;metabolic process;regulation of biological process;response to stimul	chromosome;cytoplasm;nucleus	catalytic activity;DNA binding;metal ion binding;nucleotide binding;protein binding;RNA binding
475	cellular homeostasis;response to stimulus;transport	cytosol	metal ion binding;protein binding;transporter activity
528	cellular homeostasis;regulation of biological process;response to stimulus;transport	cytoplasm;cytosol;membrane	catalytic activity;protein binding;transporter activity
39	metabolic process	cytoplasm;cytosol;mitochondrion;nucleus	catalytic activity;protein binding
55313	metabolic process;transport	cytoplasm;cytosol;extracellular;membrane;organelle lumen	catalytic activity;metal ion binding
4728	cell organization and biogenesis;metabolic process;response to stimulus;transport	membrane;mitochondrion;organelle lumen	catalytic activity;metal ion binding
6122	cell organization and biogenesis;metabolic process;response to stimulus;transport	cytoplasm;cytosol;nucleus;ribosome	protein binding;RNA binding;structural molecule activity
3117	regulation of biological process;response to stimulus	endoplasmic reticulum;endosome;Golgi;membrane;vacuole	metal ion binding;receptor activity;signal transducer activity
998	cell communication;cell differentiation;cell division;cell organization and biogenesis;cellular component movement;coagulation;metabo	cytoplasm;cytoskeleton;cytosol;membrane;vacuole	catalytic activity;nucleotide binding;protein binding
127281	metabolic process	cytoplasm;cytosol;endoplasmic reticulum	antioxidant activity;catalytic activity;nucleotide binding
6448	metabolic process	organelle lumen;vacuole	catalytic activity;metal ion binding
10652	cell organization and biogenesis;transport	cytoplasm;cytosol;endoplasmic reticulum;endosome;Golgi;membrane;mitochondrion	catalytic activity;protein binding
29952	metabolic process;transport	cytosol;extracellular;Golgi;organelle lumen;vacuole	catalytic activity
57819	metabolic process	cytosol;nucleus;spliceosomal complex	protein binding;RNA binding
58527			
9798	cell division;cell organization and biogenesis;regulation of biological process;transport	chromosome;cytoplasm;cytoskeleton;cytosol;extracellular;nucleus;organelle lumen	protein binding
23760	cell organization and biogenesis;metabolic process;transport	cytoplasm;Golgi;membrane	transporter activity

22818	transport	cytoplasm;cytosol;Golgi;membrane	
58517	metabolic process;regulation of biological process	cytoplasm;nucleus;spliceosomal complex	protein binding;RNA binding
8530	regulation of biological process;response to stimulus	cytoplasm;extracellular	enzyme regulator activity;protein binding
1936	metabolic process;regulation of biological process;response to stimulus	cytoplasm;cytosol;endoplasmic reticulum;nucleus	DNA binding;protein binding;RNA binding;signal transducer activity
22934	metabolic process	cytosol	catalytic activity;protein binding
6136	cell organization and biogenesis;metabolic process;transport	cytosol;membrane;ribosome	protein binding;RNA binding;structural molecule activity
6201	cell organization and biogenesis;metabolic process;regulation of biological process;transport	cytoplasm;cytoskeleton;cytosol;membrane;nucleus;ribosome	protein binding;RNA binding;structural molecule activity
3248	metabolic process;regulation of biological process;response to stimulus	cytoplasm;cytosol;membrane	catalytic activity;nucleotide binding;protein binding;receptor activity;signal transducer activity
10326	defense response;regulation of biological process;response to stimulus;transport	membrane	protein binding
945	cell communication;regulation of biological process;response to stimulus;transport	membrane;nucleus	protein binding;receptor activity
6642	cell organization and biogenesis;metabolic process;regulation of biological process;transport	cytoplasm;cytosol;endosome;Golgi;membrane;vacuole	protein binding
26136	regulation of biological process	cytoplasm;cytosol;membrane;nucleus	metal ion binding;protein binding;RNA binding
	regulation of biological process;response to stimulus		catalytic activity;nucleotide binding;protein binding;signal transducer activity
3127	regulation of biological process;response to stimulus	endoplasmic reticulum;endosome;Golgi;membrane;vacuole	
29086	cell division;cell organization and biogenesis;cell proliferation;metabolic process;regulation of biological process;response to stimulus	cytoplasm;cytosol;nucleus	protein binding
4597	metabolic process;regulation of biological process	cytosol	catalytic activity;nucleotide binding;protein binding
11313	cellular component movement;metabolic process	cytoplasm;cytosol	catalytic activity;protein binding
10589	metabolic process;regulation of biological process	nucleus	DNA binding;protein binding
57727	metabolic process;regulation of biological process	cytoskeleton;nucleus	protein binding;RNA binding
30968	cell organization and biogenesis;cellular homeostasis;metabolic process;regulation of biological process;response to stimulus;transpo	cytoplasm;cytoskeleton;membrane;mitochondrion	protein binding
683	metabolic process;regulation of biological process;response to stimulus;transport	extracellular;membrane	catalytic activity
10907	cell division;cell organization and biogenesis;cellular homeostasis;metabolic process;regulation of biological process	cytosol;membrane;nucleus;spliceosomal complex	protein binding
9815	regulation of biological process		enzyme regulator activity;metal ion binding;protein binding
51765	cell communication;cell death;cell organization and biogenesis;metabolic process;regulation of biological process;response to stimulu	cytoplasm;cytosol;Golgi;membrane	catalytic activity;metal ion binding;nucleotide binding;protein binding;signal transducer activity
11332	metabolic process	cytoplasm;cytosol;mitochondrion	catalytic activity;protein binding
5695	metabolic process;regulation of biological process;response to stimulus;transport	cytoplasm;cytosol;extracellular;nucleus;organelle lumen;proteasome	catalytic activity;nucleotide binding;protein binding
6434	cell organization and biogenesis;cellular homeostasis;metabolic process;regulation of biological process;response to stimulus	membrane;nucleus;spliceosomal complex	protein binding;RNA binding
9114	cell organization and biogenesis;cellular homeostasis;regulation of biological process;response to stimulus;transport	endosome;membrane	catalytic activity;protein binding;transporter activity
7343	metabolic process;regulation of biological process	nucleus	DNA binding;protein binding;RNA binding
51667	metabolic process;regulation of biological process;response to stimulus	cytosol;nucleus	protein binding
9980	cell organization and biogenesis;development;transport	cytosol;endosome;membrane	
10849	metabolic process;regulation of biological process;response to stimulus	chromosome;cytosol;mitochondrion;nucleus	catalytic activity;RNA binding
9879	metabolic process;regulation of biological process	cytoplasm;membrane;nucleus	catalytic activity;nucleotide binding;RNA binding
23211			metal ion binding;RNA binding
10949	defense response;metabolic process;regulation of biological process;response to stimulus	nucleus	protein binding;RNA binding
51172	cell organization and biogenesis;metabolic process;transport	Golgi;membrane	catalytic activity;protein binding
10105	cell death;cell organization and biogenesis;metabolic process;regulation of biological process;response to stimulus	membrane;mitochondrion;organelle lumen	catalytic activity;protein binding
7076	cell organization and biogenesis;metabolic process;regulation of biological process;response to stimulus;transport	extracellular;organelle lumen	enzyme regulator activity;metal ion binding;protein binding
6817	metabolic process	cytoplasm;cytosol	catalytic activity;protein binding
51291	regulation of biological process;response to stimulus	cytosol;membrane	enzyme regulator activity;metal ion binding;protein binding
6168	cell organization and biogenesis;metabolic process;transport	cytosol;nucleus;ribosome	metal ion binding;protein binding;RNA binding;structural molecule activity
7913	cell organization and biogenesis;metabolic process;regulation of biological process;response to stimulus	nucleus	DNA binding;protein binding;RNA binding
3040; 3039	cell organization and biogenesis;metabolic process;regulation of biological process;response to stimulus;transport	cytosol;extracellular;membrane;organelle lumen	antioxidant activity;catalytic activity;metal ion binding;protein binding;transporter activity
5901	cell division;cell organization and biogenesis;metabolic process;regulation of biological process;response to stimulus;transport	chromosome;cytoplasm;cytosol;endosome;membrane;nucleus	catalytic activity;metal ion binding;nucleotide binding;protein binding;RNA binding;transporter activ
10981	cell organization and biogenesis;regulation of biological process;response to stimulus;transport	cytosol;endoplasmic reticulum;endosome;Golgi;membrane;mitochondrion;organelle lumen	catalytic activity;nucleotide binding;protein binding
1036	defense response;metabolic process;response to stimulus;transport	cytosol	catalytic activity;metal ion binding
515	cell organization and biogenesis;metabolic process;transport	membrane;mitochondrion;nucleus;organelle lumen	catalytic activity;protein binding;transporter activity
6301	metabolic process;regulation of biological process	cytoplasm;cytosol;nucleus	catalytic activity;DNA binding;nucleotide binding;protein binding;RNA binding
3852	cell death;cell organization and biogenesis;metabolic process	cytoplasm;cytosol;membrane;mitochondrion;nucleus	catalytic activity;motor activity;protein binding;structural molecule activity
51762	cell organization and biogenesis;regulation of biological process;response to stimulus;transport	endosome;membrane;mitochondrion	catalytic activity;nucleotide binding;protein binding
9040	metabolic process;regulation of biological process;response to stimulus	cytoplasm;cytosol	catalytic activity;nucleotide binding;protein binding
51608	regulation of biological process;transport	cytoplasm;cytosol	protein binding
9931		membrane;nucleus	catalytic activity;DNA binding;metal ion binding;nucleotide binding;protein binding;RNA binding
9246	metabolic process;regulation of biological process;response to stimulus	cytosol	catalytic activity;protein binding
645	metabolic process;transport	cytoplasm;cytosol;membrane	catalytic activity
3163	cellular homeostasis;metabolic process;response to stimulus;transport	endoplasmic reticulum;membrane	catalytic activity;metal ion binding;protein binding
57674	cell organization and biogenesis;metabolic process;regulation of biological process	cytoplasm;cytosol;membrane	catalytic activity;metal ion binding;protein binding
9749	regulation of biological process;transport	membrane	enzyme regulator activity;protein binding
23186	cell organization and biogenesis;coagulation;metabolic process;regulation of biological process	nucleus	catalytic activity;DNA binding;protein binding
4702	cell organization and biogenesis;metabolic process;transport	membrane;mitochondrion	catalytic activity;protein binding
10788	cell organization and biogenesis;regulation of biological process;response to stimulus;transport	cell surface;cytoplasm;cytoskeleton;cytosol;membrane	enzyme regulator activity;protein binding
10659	cell organization and biogenesis;metabolic process;regulation of biological process	cytoplasm;nucleus	RNA binding
79869	cell organization and biogenesis;metabolic process	membrane;nucleus	protein binding;RNA binding
5069	metabolic process;response to stimulus	extracellular	catalytic activity;metal ion binding
7150	cell death;cell division;cell organization and biogenesis;metabolic process;regulation of biological process;response to stimulus	chromosome;cytoplasm;nucleus	catalytic activity;DNA binding;nucleotide binding;protein binding;RNA binding
1646	cell differentiation;metabolic process;regulation of biological process;response to stimulus	cytoplasm	catalytic activity
22931	cell organization and biogenesis;development;regulation of biological process;response to stimulus;transport	cytosol;Golgi;membrane	catalytic activity;nucleotide binding;protein binding
3843	regulation of biological process;response to stimulus;transport	cytoplasm;membrane;nucleus	enzyme regulator activity;protein binding;RNA binding;transporter activity
29937	regulation of biological process	extracellular;membrane	metal ion binding;protein binding
23111	cell division;cell organization and biogenesis;regulation of biological process	cytoplasm;cytosol;membrane	protein binding
8514	cell differentiation;metabolic process;regulation of biological process;transport	cytoplasm;cytoskeleton;cytosol;membrane	catalytic activity;transporter activity
4340		membrane	receptor activity
55335		mitochondrion	
283742	metabolic process;regulation of biological process	cytoplasm;nucleus	catalytic activity;protein binding;RNA binding
9984	cell death;metabolic process;regulation of biological process;response to stimulus;transport	cytoplasm;cytosol;nucleus	DNA binding;protein binding;RNA binding
1892	metabolic process	mitochondrion;organelle lumen	catalytic activity;protein binding
27095	cell organization and biogenesis;transport	cytosol;endoplasmic reticulum;Golgi;membrane	protein binding
8078	metabolic process;regulation of biological process	cytosol;vacuole	catalytic activity;metal ion binding;protein binding
1495	cell organization and biogenesis;regulation of biological process;response to stimulus	cytoplasm;cytoskeleton;cytosol;Golgi;membrane	protein binding;RNA binding;structural molecule activity
5715	cell organization and biogenesis;metabolic process;regulation of biological process;response to stimulus;transport	cytoplasm;cytosol;nucleus	protein binding
51024	cell death;cell organization and biogenesis;cellular homeostasis;metabolic process;regulation of biological process;response to stimu	endoplasmic reticulum;membrane;mitochondrion	protein binding
23114	cell organization and biogenesis;cellular component movement;transport	membrane	protein binding
1432	cell death;cell differentiation;cell organization and biogenesis;cellular component movement;metabolic process;regulation of biologica	cytoplasm;cytosol;extracellular;mitochondrion;nucleus;organelle lumen	catalytic activity;nucleotide binding;protein binding;signal transducer activity
391059	metabolic process;transport	membrane	catalytic activity;metal ion binding
8773	cell organization and biogenesis;metabolic process;transport	cytoplasm;extracellular;membrane;vacuole	catalytic activity;metal ion binding;protein binding
6039	defense response;metabolic process;response to stimulus	extracellular	catalytic activity
146722	regulation of biological process;response to stimulus	membrane	protein binding
8539	cell death;regulation of biological process	cytoplasm;membrane;nucleus;spliceosomal complex	protein binding;RNA binding
10152	cell organization and biogenesis;cellular component movement;metabolic process;regulation of biological process;response to stimul	cytoplasm;cytoskeleton;cytosol	DNA binding;enzyme regulator activity;protein binding
81873	cell organization and biogenesis;cellular component movement;regulation of biological process	cytoplasm;cytoskeleton	protein binding
5714	cell organization and biogenesis;metabolic process;regulation of biological process;response to stimulus;transport	cytosol;nucleus;proteasome	catalytic activity
58155	cell organization and biogenesis;metabolic process;regulation of biological process	nucleus;spliceosomal complex	RNA binding
11333	cell proliferation;regulation of biological process;response to stimulus;transport	extracellular	RNA binding
9402	cell communication;cell differentiation;cellular component movement;defense response;metabolic process;regulation of biological pro	cytoplasm;cytosol;endosome;nucleus	catalytic activity;protein binding
9589	metabolic process;regulation of biological process	membrane;nucleus	protein binding
57446	cell differentiation;regulation of biological process;response to stimulus	cytoplasm	
57470	metabolic process		catalytic activity;protein binding;RNA binding
23033	transport	cytosol;endosome	
4671	cell death;cell organization and biogenesis;defense response;metabolic process;regulation of biological process;response to stimulus	cytoplasm;membrane;nucleus	catalytic activity;enzyme regulator activity;metal ion binding;nucleotide binding;protein binding
84955		cytoplasm;cytosol;nucleus	protein binding
10890	cell differentiation;cell organization and biogenesis;regulation of biological process;response to stimulus;transport	cytosol;endoplasmic reticulum;endosome;Golgi;membrane	catalytic activity;nucleotide binding;protein binding

3735	metabolic process;regulation of biological process;response to stimulus	cytoplasm;cytosol;extracellular;membrane;mitochondrion;nucleus;organelle lumen	catalytic activity;nucleotide binding;protein binding;RNA binding
23085	development;metabolic process;regulation of biological process;transport	cytoplasm;endoplasmic reticulum;Golgi;membrane	protein binding;transporter activity
5336	cell differentiation;cellular homeostasis;metabolic process;regulation of biological process;response to stimulus;transport	cytosol;membrane	catalytic activity;protein binding;signal transducer activity
5573	cell organization and biogenesis;cell proliferation;coagulation;metabolic process;regulation of biological process;response to stimulus	cytoplasm;cytosol;membrane	catalytic activity;enzyme regulator activity;nucleotide binding;protein binding
4482	metabolic process;response to stimulus	cytoplasm;cytoskeleton;cytosol;membrane;mitochondrion;nucleus	catalytic activity
1777	cell death;cell differentiation;development;metabolic process	vacuole	catalytic activity;DNA binding
2620	cell death;regulation of biological process	cytoplasm;cytoskeleton;cytosol;membrane	protein binding
3182	cell organization and biogenesis;regulation of biological process	cytoplasm;nucleus	DNA binding;protein binding;RNA binding
5203	metabolic process	cytoplasm;cytosol;mitochondrion;nucleus	protein binding
51155		cytoplasm;membrane;nucleus	
522	cell organization and biogenesis;metabolic process;transport	membrane;mitochondrion	catalytic activity;transporter activity
10432; 100526737	cell organization and biogenesis;defense response;metabolic process;regulation of biological process;response to stimulus	cytoplasm;nucleus	protein binding;RNA binding
345	cell organization and biogenesis;metabolic process;regulation of biological process;response to stimulus;transport	endosome;extracellular	enzyme regulator activity;protein binding
145165		cytoplasm	protein binding
6234	cell organization and biogenesis;metabolic process;transport	cytosol;ribosome	protein binding;RNA binding;structural molecule activity
9444	cell differentiation;development;metabolic process;regulation of biological process;transport	cytoplasm;nucleus	protein binding;RNA binding
93100	metabolic process;response to stimulus;transport	cytoplasm;cytosol;extracellular;organelle lumen	catalytic activity;protein binding
2879	cell organization and biogenesis;development;metabolic process;response to stimulus	cytoplasm;cytosol;mitochondrion;nucleus	antioxidant activity;catalytic activity;protein binding
23078		mitochondrion	catalytic activity;nucleotide binding
6646	cell differentiation;cell organization and biogenesis;metabolic process;regulation of biological process;transport	endoplasmic reticulum;membrane	catalytic activity;protein binding
1069	cell division;cell organization and biogenesis;metabolic process;regulation of biological process;response to stimulus	cytoplasm;cytoskeleton;cytosol;nucleus	metal ion binding;protein binding
1191	cell death;cell organization and biogenesis;cell proliferation;defense response;metabolic process;regulation of biological process;resp	cell surface;cytoplasm;cytosol;endoplasmic reticulum;extracellular;Golgi;membrane;mitochondrion;nucleus;org	catalytic activity;protein binding
8544	cell differentiation;metabolic process;regulation of biological process	cytoplasm;cytosol;nucleus	catalytic activity;metal ion binding;protein binding
63929	metabolic process	mitochondrion	catalytic activity;metal ion binding
79366	cell organization and biogenesis;metabolic process;regulation of biological process	chromosome;nucleus	DNA binding;protein binding;RNA binding
5303	metabolic process	cytoplasm;cytoskeleton;mitochondrion;nucleus;organelle lumen	catalytic activity;DNA binding;protein binding;RNA binding
10043	transport	cytoplasm;cytosol;endosome;membrane	protein binding
3145	metabolic process	cytoplasm;cytosol	catalytic activity
3845	cell communication;cell differentiation;cell organization and biogenesis;cellular component movement;metabolic process;regulation of	cytoplasm;cytosol;membrane;mitochondrion	catalytic activity;nucleotide binding;protein binding
1739	cell communication;cell organization and biogenesis;cell proliferation;metabolic process;regulation of biological process;response to s	cytoplasm;cytosol;endoplasmic reticulum;Golgi;membrane;nucleus	catalytic activity;protein binding;structural molecule activity;transporter activity
1522	cell organization and biogenesis;metabolic process;regulation of biological process;transport	cell surface;endoplasmic reticulum;extracellular;membrane;organelle lumen;vacuole	catalytic activity;protein binding
10592	cell division;cell organization and biogenesis;metabolic process;regulation of biological process	chromosome;cytoplasm;cytosol;nucleus	catalytic activity;motor activity;nucleotide binding;protein binding
4267	regulation of biological process	cytoplasm;membrane	protein binding
6728	cell organization and biogenesis;response to stimulus;transport	cytoplasm;cytosol	protein binding;RNA binding
1810	cell organization and biogenesis;metabolic process;regulation of biological process	nucleus	DNA binding;protein binding
5903	cell organization and biogenesis;metabolic process;regulation of biological process;response to stimulus;transport	cytoplasm;cytosol;membrane;mitochondrion;nucleus	catalytic activity;enzyme regulator activity;metal ion binding;protein binding;RNA binding
7706	defense response;metabolic process;regulation of biological process;response to stimulus	cytoplasm;cytosol	catalytic activity;metal ion binding;motor activity;protein binding;RNA binding
54939	metabolic process;regulation of biological process	cytoplasm;nucleus	protein binding
11314	regulation of biological process;response to stimulus;transport	membrane	protein binding;receptor activity;signal transducer activity
55660	cell division;cell organization and biogenesis;cellular component movement;metabolic process;regulation of biological process	cytosol;membrane;nucleus;spliceosomal complex	protein binding;RNA binding
10980	cell organization and biogenesis;metabolic process;response to stimulus	cytoplasm;cytosol;nucleus	protein binding
10254	cell communication;cell organization and biogenesis;metabolic process;regulation of biological process;response to stimulus;transpo	cytoplasm;cytosol;endosome;membrane	protein binding
4301	cell communication;cell organization and biogenesis;regulation of biological process;response to stimulus	cytosol;membrane	protein binding
2617	metabolic process	cytoplasm;cytosol;extracellular;mitochondrion;organelle lumen	catalytic activity;nucleotide binding;protein binding
8829	cell communication;cell differentiation;cell growth;cell organization and biogenesis;cellular component movement;development;regula	cell surface;cytosol;endosome;extracellular;membrane	catalytic activity;enzyme regulator activity;metal ion binding;protein binding;receptor activity;signal
414061			
3798	cell communication;cellular component movement;metabolic process;transport	cytoplasm;cytoskeleton;cytosol;membrane	catalytic activity;motor activity;nucleotide binding;protein binding
3612	metabolic process;regulation of biological process;response to stimulus	cytoplasm;cytosol	catalytic activity;metal ion binding;protein binding
4501	regulation of biological process;response to stimulus	cytoplasm;nucleus	metal ion binding;protein binding
5437	metabolic process;regulation of biological process;response to stimulus	cytosol;nucleus	catalytic activity;DNA binding
374454	cell death;cell organization and biogenesis	cytoskeleton;cytosol	structural molecule activity
7979			
30844	cell organization and biogenesis;regulation of biological process;response to stimulus;transport	endoplasmic reticulum;endosome;membrane;nucleus	metal ion binding;nucleotide binding;protein binding
58472	metabolic process	membrane;mitochondrion	catalytic activity
3857	cell death;cell organization and biogenesis	cytosol;membrane;nucleus	structural molecule activity
83592	metabolic process	cytoplasm;cytosol;Golgi	catalytic activity
84991	metabolic process;regulation of biological process;response to stimulus	nucleus;spliceosomal complex	protein binding;RNA binding
2195	cell communication;cell organization and biogenesis;cellular component movement	cell surface;cytoplasm;membrane;nucleus	metal ion binding;protein binding
5700	metabolic process;regulation of biological process;response to stimulus;transport	cytoplasm;cytosol;membrane;nucleus;proteasome	catalytic activity;nucleotide binding;protein binding;RNA binding
118611		cytoplasm;cytoskeleton;cytosol;membrane	protein binding
4507	metabolic process;regulation of biological process;response to stimulus	cytoplasm;cytosol;nucleus	catalytic activity;protein binding
51143	cell division;cell organization and biogenesis;cellular component movement;regulation of biological process;transport	chromosome;cytoplasm;cytoskeleton;cytosol;membrane	catalytic activity;motor activity;nucleotide binding;protein binding;RNA binding
3029	metabolic process	cytoplasm;cytosol;mitochondrion;organelle lumen	catalytic activity;metal ion binding;protein binding
23	defense response;metabolic process;regulation of biological process;response to stimulus;transport	cytoplasm;cytosol;membrane;nucleus;ribosome	catalytic activity;nucleotide binding;protein binding;RNA binding;translation regulator activity
87769	metabolic process		catalytic activity;protein binding
51206	cellular component movement;coagulation;regulation of biological process;response to stimulus	cell surface;membrane	protein binding;receptor activity;signal transducer activity
9590	regulation of biological process;response to stimulus;transport	cytoplasm;cytoskeleton;cytosol;membrane	protein binding
10075	cell differentiation;cell organization and biogenesis;metabolic process;response to stimulus;transport	cytoplasm;cytosol;extracellular;membrane;nucleus;organelle lumen	catalytic activity;DNA binding;protein binding;RNA binding
4134	cell division;cell organization and biogenesis;cellular component movement;regulation of biological process	cytoplasm;cytoskeleton	protein binding;RNA binding;structural molecule activity
645683	metabolic process	nucleus;ribosome	RNA binding;structural molecule activity
125950	metabolic process	cytoplasm;nucleus	protein binding;RNA binding
8895	metabolic process;regulation of biological process;response to stimulus;transport	cytoplasm;cytosol;membrane;mitochondrion;nucleus	catalytic activity;protein binding;RNA binding;transporter activity
	metabolic process	ribosome	RNA binding;structural molecule activity
7264	metabolic process	cytoplasm;cytosol	catalytic activity;protein binding
5965	metabolic process;response to stimulus	chromosome;cytoplasm;membrane;nucleus	catalytic activity;DNA binding;nucleotide binding;protein binding
1410	cell death;cell organization and biogenesis;metabolic process;regulation of biological process;response to stimulus	cell surface;cytoplasm;cytoskeleton;cytosol;Golgi;membrane;mitochondrion;nucleus	metal ion binding;protein binding;structural molecule activity
4925		cytoplasm;cytosol;endoplasmic reticulum;Golgi;membrane;nucleus	DNA binding;metal ion binding;protein binding
10540	cell organization and biogenesis;cell proliferation;regulation of biological process;transport	chromosome;cytoplasm;cytoskeleton;cytosol;membrane	catalytic activity;motor activity;protein binding
27161	cell organization and biogenesis;metabolic process;regulation of biological process;response to stimulus	cytoplasm;cytosol;membrane;nucleus	catalytic activity;DNA binding;metal ion binding;protein binding;RNA binding
8894	cell proliferation;metabolic process;transport	cytoplasm;cytosol;nucleus	metal ion binding;protein binding;RNA binding
5306	cell organization and biogenesis;metabolic process;regulation of biological process;response to stimulus;transport	cytoplasm;cytosol	transporter activity
11052	cell organization and biogenesis;metabolic process	membrane;nucleus	protein binding;RNA binding
51593	cell proliferation;metabolic process;regulation of biological process;response to stimulus	cytoplasm;nucleus	DNA binding;protein binding;RNA binding
9861	metabolic process;regulation of biological process;response to stimulus;transport	cytosol;extracellular;organelle lumen;proteasome	catalytic activity;enzyme regulator activity;protein binding
5250	transport	membrane;mitochondrion	protein binding;transporter activity
79165			protein binding
10801	cell division;cell organization and biogenesis;regulation of biological process	cytoplasm;cytoskeleton	catalytic activity;nucleotide binding;protein binding
669	metabolic process;regulation of biological process	cytosol	catalytic activity
9696	cell organization and biogenesis;regulation of biological process	cytoplasm;cytoskeleton;cytosol;membrane	protein binding;structural molecule activity
175	metabolic process;transport	endoplasmic reticulum;extracellular;organelle lumen;vacuole	catalytic activity;protein binding
2339	cell organization and biogenesis;metabolic process;regulation of biological process;response to stimulus	cytoplasm;cytosol;membrane	catalytic activity;protein binding
3054	cell organization and biogenesis;metabolic process;regulation of biological process;response to stimulus	cytoplasm;membrane;nucleus	catalytic activity;protein binding
6829	cell organization and biogenesis;development;metabolic process;regulation of biological process;response to stimulus	nucleus	DNA binding;protein binding;RNA binding
58477	regulation of biological process;response to stimulus	cytoplasm;endoplasmic reticulum;membrane	nucleotide binding
56301	cellular component movement;transport	membrane	transporter activity
33; 552900; 10798	cell organization and biogenesis;metabolic process;regulation of biological process;response to stimulus	cytoplasm;cytosol;nucleus	protein binding
3162	cell communication;cell death;cell organization and biogenesis;cell proliferation;cellular homeostasis;metabolic process;regulation of	cytosol;endoplasmic reticulum;membrane;nucleus	catalytic activity;metal ion binding;protein binding;signal transducer activity
25777	cell organization and biogenesis;cellular component movement;regulation of biological process;transport	chromosome;endosome;membrane;nucleus	protein binding
3512	cellular component movement;defense response;regulation of biological process;response to stimulus;transport	extracellular	DNA binding;protein binding

8832	cellular component movement;defense response;regulation of biological process;response to stimulus	membrane	protein binding
4801	metabolic process;regulation of biological process	nucleus	DNA binding;protein binding
7454	cell organization and biogenesis;cellular component movement;coagulation;defense response;regulation of biological process;respor	cytoplasm;cytoskeleton;cytosol;membrane	enzyme regulator activity;protein binding
145553	metabolic process		catalytic activity;metal ion binding;receptor activity;signal transducer activity
6217	cell organization and biogenesis;metabolic process;transport	cytosol;membrane;ribosome	protein binding;RNA binding;structural molecule activity
116984	regulation of biological process;response to stimulus	cytoplasm;cytosol	enzyme regulator activity;metal ion binding;protein binding
10992	metabolic process	nucleus;spliceosomal complex	protein binding;RNA binding
9524	metabolic process	cytoplasm;endoplasmic reticulum;membrane;nucleus	catalytic activity;protein binding
8455	defense response;regulation of biological process;response to stimulus	cytoplasm;extracellular;membrane	protein binding;receptor activity
2634	regulation of biological process;response to stimulus	cytoplasm;cytoskeleton;cytosol;Golgi;membrane	catalytic activity;nucleotide binding;protein binding
9343	cell organization and biogenesis;metabolic process;response to stimulus	cytosol;membrane;nucleus;spliceosomal complex	catalytic activity;nucleotide binding;protein binding;RNA binding
57523	metabolic process	membrane	
6166	metabolic process	cytoplasm;cytosol;membrane;nucleus;ribosome	protein binding;structural molecule activity
5870	cellular component movement;metabolic process;regulation of biological process;response to stimulus;transport	cytosol;Golgi;membrane	catalytic activity;nucleotide binding;protein binding
3430	regulation of biological process;response to stimulus	cytosol;nucleus	protein binding
408	cell death;cell organization and biogenesis;metabolic process;regulation of biological process;response to stimulus;transport	chromosome;cytoplasm;cytosol;membrane;nucleus	catalytic activity;DNA binding;enzyme regulator activity;protein binding
64077	metabolic process	cytoplasm;cytosol;nucleus	catalytic activity;metal ion binding;protein binding;receptor activity;signal transducer activity
341676	metabolic process;regulation of biological process		catalytic activity;metal ion binding;nucleotide binding
51428	cell death;cell differentiation;cell proliferation;defense response;metabolic process;regulation of biological process;response to stimul	cytoplasm;cytosol;endoplasmic reticulum;membrane;nucleus;spliceosomal complex	catalytic activity;DNA binding;metal ion binding;nucleotide binding;protein binding;RNA binding
23557	cell communication;cell organization and biogenesis;cellular component movement;cellular homeostasis;metabolic process;regulatio	cytoplasm;cytosol;Golgi;membrane;vacuole	protein binding
27074	regulation of biological process;response to stimulus	endosome;membrane;nucleus;vacuole	
23558	regulation of biological process;response to stimulus	cytoplasm;nucleus	DNA binding;protein binding
5634	metabolic process		catalytic activity;metal ion binding;nucleotide binding;protein binding
51506	metabolic process;response to stimulus	cytoplasm	catalytic activity;protein binding
8655	cell communication;cell death;cell organization and biogenesis;cellular component movement;metabolic process;regulation of biologic	chromosome;cytoplasm;cytoskeleton;cytosol;membrane;mitochondrion;nucleus	catalytic activity;motor activity;protein binding
2091	cell differentiation;cell organization and biogenesis;metabolic process	chromosome;membrane;nucleus	catalytic activity;protein binding;RNA binding
91298	cell differentiation		
28988	cell organization and biogenesis;regulation of biological process;response to stimulus;transport	cytoplasm;cytoskeleton;cytosol;endosome;extracellular;Golgi;membrane;organelle lumen	enzyme regulator activity;protein binding
100302736; 51014	transport	endoplasmic reticulum;Golgi;membrane	
10960	regulation of biological process;transport	cell surface;endoplasmic reticulum;Golgi;membrane	metal ion binding;protein binding
8436	cell organization and biogenesis	cytoplasm;cytosol;membrane	protein binding
6625	metabolic process;regulation of biological process;response to stimulus	cytoplasm;nucleus;spliceosomal complex	protein binding;RNA binding
293	cell death;cell organization and biogenesis;regulation of biological process;response to stimulus;transport	membrane;mitochondrion;nucleus	protein binding;transporter activity
8106	metabolic process;regulation of biological process;response to stimulus;transport	cytoplasm;nucleus	protein binding;RNA binding
2729	cell organization and biogenesis;cellular homeostasis;metabolic process;regulation of biological process;response to stimulus	cytosol	catalytic activity;metal ion binding;nucleotide binding;protein binding
6207	cell organization and biogenesis;metabolic process;regulation of biological process;transport	cytosol;membrane;nucleus;ribosome	protein binding;RNA binding;structural molecule activity
26472	defense response;regulation of biological process;response to stimulus	cytoplasm	enzyme regulator activity
51110	metabolic process	mitochondrion;organelle lumen	catalytic activity;metal ion binding;protein binding;RNA binding
126208	metabolic process;regulation of biological process	nucleus	DNA binding;metal ion binding
388946		endoplasmic reticulum;membrane	
10951	regulation of biological process	chromosome;nucleus	protein binding
6160	cell organization and biogenesis;metabolic process;transport	cytosol;membrane;ribosome	protein binding;RNA binding;structural molecule activity
1475	cell death;cell differentiation;metabolic process;regulation of biological process	cytoplasm;cytoskeleton;cytosol	enzyme regulator activity;protein binding;structural molecule activity
10910	regulation of biological process	chromosome;cytoplasm;cytosol;nucleus	protein binding
3281	cell differentiation;regulation of biological process	cytoskeleton;nucleus	protein binding
23526	regulation of biological process;response to stimulus;transport	cytoplasm;cytosol;extracellular;membrane;organelle lumen	enzyme regulator activity;metal ion binding;protein binding
91754	cell division;cell organization and biogenesis;metabolic process	cytoplasm;cytosol;nucleus	catalytic activity;metal ion binding;nucleotide binding;protein binding
			metal ion binding
115361		cytoplasm;Golgi;membrane;nucleus	catalytic activity;nucleotide binding
51237	cell death;cell organization and biogenesis;regulation of biological process	cytoplasm;endoplasmic reticulum;extracellular;organelle lumen	protein binding
283417	cell differentiation;development;metabolic process	membrane;nucleus	catalytic activity
8603			
23042; 102724985	metabolic process	Golgi	catalytic activity;protein binding
1486	metabolic process	vacuole	catalytic activity
6141	cell organization and biogenesis;metabolic process;transport	cytoplasm;cytosol;endoplasmic reticulum;membrane;nucleus;ribosome	RNA binding;structural molecule activity
10666	regulation of biological process;response to stimulus	cell surface;membrane	protein binding;receptor activity
29082	cell communication;cell organization and biogenesis;metabolic process;regulation of biological process;response to stimulus;transpor	cytoplasm;cytosol;endosome;membrane;nucleus	protein binding
3159	cell organization and biogenesis;metabolic process;regulation of biological process;response to stimulus;transport	chromosome;cytosol;nucleus	catalytic activity;DNA binding;protein binding
3431	metabolic process;regulation of biological process;response to stimulus	nucleus	DNA binding;metal ion binding;protein binding;signal transducer activity
54832	cell organization and biogenesis;transport	cytoplasm;cytosol;membrane;mitochondrion	
54414	metabolic process;regulation of biological process	extracellular;vacuole	catalytic activity
9482	cell organization and biogenesis;regulation of biological process;transport	cytosol;endoplasmic reticulum;endosome;membrane	protein binding
8548	cell organization and biogenesis;cell proliferation;regulation of biological process;transport	cytoplasm;Golgi;membrane;nucleus;organelle lumen	DNA binding;protein binding
84687	cell differentiation;cell organization and biogenesis;cellular component movement;development;metabolic process;regulation of biolog	cytoplasm;cytoskeleton;membrane;nucleus	enzyme regulator activity;protein binding;structural molecule activity
6133	cell organization and biogenesis;metabolic process;transport	cytosol;membrane;nucleus;ribosome	protein binding;RNA binding;structural molecule activity
3796	cell differentiation;cell division;cell organization and biogenesis;cellular component movement;development;transport	cytoplasm;cytoskeleton;cytosol;membrane;nucleus	catalytic activity;motor activity;nucleotide binding;protein binding
79002			protein binding
9320	metabolic process;regulation of biological process;response to stimulus	cytoplasm;cytosol;nucleus	catalytic activity;protein binding
1047	cell organization and biogenesis;metabolic process	endoplasmic reticulum;membrane	metal ion binding;protein binding
1445	cell differentiation;cell organization and biogenesis;defense response;metabolic process;regulation of biological process;response to	cytoplasm;cytosol;membrane	catalytic activity;metal ion binding;nucleotide binding;protein binding
25909	cell division;cell organization and biogenesis;regulation of biological process;transport	chromosome;cytoplasm;cytosol;membrane;nucleus	DNA binding;protein binding
5201	cell organization and biogenesis;metabolic process;regulation of biological process	cytoplasm	protein binding
23028	cell organization and biogenesis;coagulation;development;metabolic process;regulation of biological process;response to stimulus	nucleus	catalytic activity;DNA binding;nucleotide binding;protein binding
978	cell organization and biogenesis;metabolic process;regulation of biological process;response to stimulus;transport	cytosol;extracellular;organelle lumen	catalytic activity;metal ion binding;protein binding
1153	cell organization and biogenesis;regulation of biological process;response to stimulus	cytoplasm;nucleus	protein binding;RNA binding;translation regulator activity
10061	transport	membrane;mitochondrion	catalytic activity;nucleotide binding;transporter activity
4905	cell organization and biogenesis;metabolic process;regulation of biological process;response to stimulus;transport	cytoplasm;cytosol;membrane	catalytic activity;metal ion binding;nucleotide binding;protein binding;RNA binding
9531	cell death;metabolic process;regulation of biological process;response to stimulus	cytoplasm;cytosol;membrane;nucleus	enzyme regulator activity;nucleotide binding;protein binding
4033	cell organization and biogenesis;transport	chromosome;cytoplasm;cytoskeleton;endoplasmic reticulum;membrane;nucleus	
51560	regulation of biological process;response to stimulus;transport	cytosol;Golgi;membrane	catalytic activity;nucleotide binding;protein binding
969	regulation of biological process;response to stimulus	membrane	metal ion binding;protein binding;receptor activity;signal transducer activity
10092	cell organization and biogenesis;cellular component movement;regulation of biological process;response to stimulus;transport	cytoplasm;cytoskeleton;cytosol;endosome;extracellular;organelle lumen	protein binding;structural molecule activity
3646	metabolic process;regulation of biological process	chromosome;cytoplasm;cytosol;membrane;nucleus	protein binding;RNA binding
9818	cell organization and biogenesis;metabolic process;regulation of biological process;transport	membrane;nucleus	protein binding;structural molecule activity;transporter activity
132	metabolic process	cytoplasm;cytosol;nucleus	catalytic activity;metal ion binding;nucleotide binding;RNA binding
10542	cell organization and biogenesis;regulation of biological process;response to stimulus	cytoplasm;cytosol;membrane;vacuole	protein binding;structural molecule activity
81609	regulation of biological process;response to stimulus;transport	cytoplasm;cytosol;endosome;membrane	protein binding
5092	cell organization and biogenesis;metabolic process;regulation of biological process	cytoplasm;cytosol;nucleus	catalytic activity;protein binding
54453	regulation of biological process;response to stimulus;transport	cytoplasm;cytosol	enzyme regulator activity
60491	cell differentiation;regulation of biological process	cytoplasm;mitochondrion;nucleus	protein binding
51386	metabolic process	cytoplasm;cytosol;membrane	protein binding;RNA binding
10476	cell organization and biogenesis;metabolic process;transport	cytosol;membrane;mitochondrion	catalytic activity;transporter activity
64802	metabolic process;response to stimulus	nucleus	catalytic activity;nucleotide binding;protein binding
10855	metabolic process;regulation of biological process;response to stimulus;transport	extracellular;membrane;nucleus;organelle lumen;vacuole	catalytic activity;protein binding
1952	cell organization and biogenesis;development;regulation of biological process;response to stimulus	cytoplasm;membrane	metal ion binding;protein binding;receptor activity;signal transducer activity
10131	metabolic process;regulation of biological process;response to stimulus	membrane;mitochondrion;organelle lumen	nucleotide binding;protein binding;RNA binding
6249	cell differentiation;cell organization and biogenesis;metabolic process;regulation of biological process;transport	chromosome;cytoplasm;cytoskeleton;cytosol;endosome;membrane	catalytic activity;metal ion binding;motor activity;protein binding
2260	cell communication;cell differentiation;cell division;cellular component movement;metabolic process;regulation of biological process;re	cytoplasm;cytosol;extracellular;membrane;nucleus	catalytic activity;nucleotide binding;protein binding;receptor activity;signal transducer activity
261726	regulation of biological process;response to stimulus	cytoplasm	protein binding
4725	cell organization and biogenesis;metabolic process;transport	membrane;mitochondrion	catalytic activity
1803	cellular component movement;defense response;metabolic process;regulation of biological process;response to stimulus	cell surface;extracellular;membrane	catalytic activity;protein binding;receptor activity
9377	metabolic process;transport	membrane;mitochondrion	catalytic activity;metal ion binding;protein binding;transporter activity

6205	cell differentiation;cell organization and biogenesis;metabolic process;transport	cytoplasm;cytosol;membrane;ribosome	protein binding;RNA binding;structural molecule activity
112942		cytoplasm;nucleus	protein binding
1359	metabolic process;regulation of biological process	extracellular	catalytic activity;metal ion binding
7994	cell differentiation;cell organization and biogenesis;metabolic process;regulation of biological process;response to stimulus	chromosome;cytosol;nucleus	catalytic activity;DNA binding;metal ion binding;protein binding
53938	metabolic process	spliceosomal complex	catalytic activity;protein binding
83473	metabolic process;response to stimulus	cytoplasm;cytoskeleton;nucleus	catalytic activity;nucleotide binding;protein binding;RNA binding
79709		endoplasmic reticulum;membrane;organelle lumen	catalytic activity
3687	cell organization and biogenesis;cellular component movement;regulation of biological process;response to stimulus;transport	cell surface;membrane	metal ion binding;protein binding;receptor activity
8905	regulation of biological process;transport	cytosol;Golgi;membrane	protein binding;transporter activity
8450	cell organization and biogenesis;metabolic process;regulation of biological process;response to stimulus	cytosol;nucleus	catalytic activity;DNA binding;protein binding
2521	metabolic process;regulation of biological process;response to stimulus	cytoplasm;nucleus	DNA binding;metal ion binding;protein binding;RNA binding
6169	cell organization and biogenesis;metabolic process;regulation of biological process;transport	cytosol;ribosome	RNA binding;structural molecule activity
1891	metabolic process	membrane;mitochondrion	catalytic activity;protein binding
115098	cell division	cytoplasm;cytoskeleton	RNA binding
26092	cell organization and biogenesis;regulation of biological process	membrane;nucleus	enzyme regulator activity;protein binding
6745	cell organization and biogenesis;regulation of biological process;transport	endoplasmic reticulum;membrane	protein binding
6155	cell organization and biogenesis;metabolic process;transport	cytosol;membrane;nucleus;ribosome	RNA binding;structural molecule activity
3094	cell death;metabolic process;regulation of biological process;response to stimulus	cytoplasm;cytoskeleton;cytosol;membrane;nucleus	catalytic activity;nucleotide binding;protein binding
5657	cell differentiation;cell organization and biogenesis;cellular component movement;coagulation;metabolic process;regulation of biologi	cytosol;extracellular;membrane;organelle lumen	catalytic activity;protein binding
387921	transport	extracellular;organelle lumen	protein binding
833	metabolic process	cytoplasm;cytosol	catalytic activity;metal ion binding;nucleotide binding;protein binding;RNA binding
9360	metabolic process	cytosol;nucleus	catalytic activity;protein binding;RNA binding
55183	cell organization and biogenesis;metabolic process;regulation of biological process;response to stimulus	chromosome;cytoplasm;cytoskeleton;membrane;nucleus	protein binding
4124	cell organization and biogenesis;metabolic process;regulation of biological process	Golgi;membrane	catalytic activity;metal ion binding
9667	cell differentiation;metabolic process;regulation of biological process	cytoplasm;nucleus	DNA binding;protein binding;RNA binding
54732	cell organization and biogenesis;regulation of biological process;transport	endoplasmic reticulum;Golgi;membrane	protein binding
7385	metabolic process;transport	membrane;mitochondrion	catalytic activity;metal ion binding;protein binding
6810	cell organization and biogenesis;regulation of biological process;response to stimulus;transport	cell surface;cytoplasm;cytosol;endosome;membrane;vacuole	enzyme regulator activity;protein binding
56267	metabolic process	mitochondrion	catalytic activity;protein binding;RNA binding
9669	metabolic process;regulation of biological process	cytoplasm;cytosol;nucleus	catalytic activity;metal ion binding;nucleotide binding;protein binding;RNA binding
3635	cell death;cellular component movement;metabolic process;regulation of biological process;response to stimulus	cytoplasm;cytoskeleton;cytosol;membrane	catalytic activity;protein binding
56893	metabolic process;regulation of biological process	cytoplasm;cytosol;endoplasmic reticulum;membrane;nucleus;proteasome;vacuole	protein binding
26519	cell organization and biogenesis;transport	membrane;mitochondrion	metal ion binding;protein binding;transporter activity
5874	regulation of biological process;response to stimulus;transport	Golgi;membrane	catalytic activity;nucleotide binding;protein binding
10228	cell organization and biogenesis;regulation of biological process;transport	cytosol;endosome;Golgi;membrane	protein binding
6135	cell organization and biogenesis;metabolic process;regulation of biological process;transport	cytoplasm;cytosol;membrane;nucleus;ribosome	protein binding;RNA binding;structural molecule activity
6228	cell organization and biogenesis;metabolic process;transport	cytosol;membrane;ribosome	protein binding;RNA binding;structural molecule activity
51115		cytoplasm;cytoskeleton;mitochondrion	protein binding
7518	metabolic process;regulation of biological process;response to stimulus	chromosome;cytosol;nucleus	catalytic activity;DNA binding;motor activity;protein binding
201294	defense response;regulation of biological process;response to stimulus;transport	cytoplasm;cytosol;endoplasmic reticulum;endosome;extracellular;membrane;organelle lumen;vacuole	protein binding
1781	cell division;cell organization and biogenesis;cellular component movement;regulation of biological process;transport	cytoplasm;cytoskeleton;cytosol;extracellular;Golgi;mitochondrion;organelle lumen	catalytic activity;motor activity;protein binding
54472	cell communication;cell differentiation;defense response;metabolic process;regulation of biological process;response to stimulus;tran	cytoplasm;cytosol;extracellular;organelle lumen	protein binding;signal transducer activity
128240	cell organization and biogenesis;metabolic process	cytosol;extracellular;mitochondrion;nucleus;organelle lumen	catalytic activity;metal ion binding;nucleotide binding;protein binding
1788	cell death;cell differentiation;cell organization and biogenesis;metabolic process;regulation of biological process;response to stimulus	chromosome;cytoplasm;nucleus	catalytic activity;DNA binding;metal ion binding;protein binding
58511	metabolic process	cytoplasm;extracellular;vacuole	catalytic activity
55004	cell organization and biogenesis;regulation of biological process;response to stimulus;transport	endosome;membrane;vacuole	protein binding;structural molecule activity
9219	cell organization and biogenesis;metabolic process;regulation of biological process	membrane;nucleus	catalytic activity;DNA binding;metal ion binding;protein binding
4809	cell organization and biogenesis;metabolic process	nucleus;spliceosomal complex	protein binding;RNA binding
159	metabolic process;response to stimulus	cytoplasm;cytosol;membrane	catalytic activity;metal ion binding;nucleotide binding;protein binding
10211	cell organization and biogenesis;regulation of biological process;response to stimulus;transport	cytoskeleton;endosome;membrane	protein binding
274	cell differentiation;cell organization and biogenesis;cell proliferation;development;regulation of biological process;transport	cytoplasm;cytoskeleton;cytosol;membrane;nucleus	protein binding
9221	cell organization and biogenesis;metabolic process;regulation of biological process	cytoplasm;nucleus	nucleotide binding;protein binding;RNA binding
23118	metabolic process;regulation of biological process;response to stimulus	cytoplasm;cytosol;membrane	metal ion binding;protein binding
5917	metabolic process	cytoplasm;cytosol;membrane;mitochondrion;nucleus	catalytic activity;nucleotide binding;protein binding;RNA binding
5442	cell organization and biogenesis;metabolic process	mitochondrion;organelle lumen	catalytic activity;DNA binding;protein binding;RNA binding
23307	cell differentiation;cell organization and biogenesis;cellular component movement;metabolic process;regulation of biological process;	cytoplasm;cytoskeleton;endosome;extracellular;Golgi;membrane;nucleus	catalytic activity;motor activity;protein binding;structural molecule activity;transporter activity
2533	cell organization and biogenesis;metabolic process;regulation of biological process;response to stimulus;transport	cytoplasm;cytoskeleton;cytosol;membrane;nucleus	protein binding
4034	regulation of biological process;response to stimulus	cytoplasm;membrane	protein binding
84661	cell organization and biogenesis;metabolic process;regulation of biological process;transport	Golgi;nucleus	catalytic activity;protein binding
317749	metabolic process	extracellular	catalytic activity
58490	metabolic process;regulation of biological process	cytosol;nucleus	protein binding
11235	cell death;cell organization and biogenesis;cellular component movement;regulation of biological process;response to stimulus	cytoplasm;cytosol;Golgi;membrane	protein binding
6907	cell organization and biogenesis;metabolic process;regulation of biological process;response to stimulus	nucleus	DNA binding;protein binding
7879	cell organization and biogenesis;cellular homeostasis;metabolic process;regulation of biological process;response to stimulus;transpo	cytoplasm;cytosol;endosome;Golgi;membrane;vacuole	catalytic activity;nucleotide binding;protein binding
210	cell organization and biogenesis;metabolic process;regulation of biological process;response to stimulus;transport	cytosol;extracellular;nucleus;organelle lumen	catalytic activity;metal ion binding;protein binding
134492	metabolic process	chromosome;cytoplasm;cytoskeleton;cytosol	protein binding
5621	cell organization and biogenesis;cellular homeostasis;metabolic process;regulation of biological process;response to stimulus	cell surface;cytoplasm;cytosol;endoplasmic reticulum;Golgi;membrane;nucleus	metal ion binding;protein binding;receptor activity;signal transducer activity
83483	metabolic process;regulation of biological process;response to stimulus	cell surface;cytoplasm;membrane	protein binding
57187	cell differentiation;cell division;cell organization and biogenesis;metabolic process;regulation of biological process;transport	nucleus	protein binding;RNA binding
8737	cell death;cell organization and biogenesis;metabolic process;regulation of biological process;response to stimulus	cytoplasm;cytosol;membrane;mitochondrion	catalytic activity;nucleotide binding;protein binding;signal transducer activity
2286	metabolic process	endoplasmic reticulum;membrane	catalytic activity;protein binding
50861	cell organization and biogenesis;metabolic process;regulation of biological process	cytoplasm;Golgi	protein binding
2823	cell differentiation;cell organization and biogenesis;cellular component movement;regulation of biological process;transport	membrane	protein binding;transporter activity
131474	metabolic process;transport	mitochondrion	catalytic activity;protein binding
7965	cell death;cell differentiation;cell organization and biogenesis;development;metabolic process;regulation of biological process	cytoplasm;cytosol;membrane;nucleus	protein binding;structural molecule activity
1536	cellular homeostasis;defense response;metabolic process;regulation of biological process;response to stimulus;transport	cytoplasm;endoplasmic reticulum;Golgi;membrane;mitochondrion	catalytic activity;metal ion binding;nucleotide binding;protein binding;transporter activity
55623	metabolic process		protein binding;RNA binding
79751	transport	membrane;mitochondrion	transporter activity
7450	cell organization and biogenesis;coagulation;metabolic process;response to stimulus;transport	endoplasmic reticulum;extracellular;organelle lumen	protein binding
2207	cell differentiation;cell organization and biogenesis;cellular component movement;coagulation;defense response;metabolic process;re	cell surface;membrane	protein binding;receptor activity;signal transducer activity
57476		membrane	
54	defense response;metabolic process;regulation of biological process;response to stimulus	cytosol;vacuole	catalytic activity;metal ion binding
8545	metabolic process;regulation of biological process	nucleus	DNA binding;protein binding
6051	metabolic process	extracellular;membrane	catalytic activity;metal ion binding
7163	cell differentiation;transport	cytoplasm;endoplasmic reticulum	metal ion binding;protein binding
51389	metabolic process;regulation of biological process;response to stimulus	cytoplasm	protein binding
958	cell organization and biogenesis;cell proliferation;cellular homeostasis;defense response;development;regulation of biological proces	cell surface;cytoplasm;extracellular;membrane	protein binding;receptor activity;signal transducer activity
54976			
4218	cell organization and biogenesis;metabolic process;regulation of biological process;response to stimulus;transport	cytoplasm;cytoskeleton;cytosol;endosome;Golgi;membrane	catalytic activity;nucleotide binding;protein binding
2060	cell organization and biogenesis;cell proliferation;metabolic process;regulation of biological process;response to stimulus;transport	cytoplasm;cytoskeleton;cytosol;endosome;membrane;nucleus	catalytic activity;DNA binding;metal ion binding;motor activity;protein binding
23052	metabolic process;transport	cytosol;extracellular;membrane	catalytic activity;metal ion binding
11157	metabolic process	cytoplasm;cytosol;nucleus;spliceosomal complex	protein binding;RNA binding
51015	metabolic process	cytoplasm	catalytic activity;protein binding
65109	metabolic process;regulation of biological process;transport	cytoplasm;cytosol;nucleus	protein binding;RNA binding;transporter activity
6923	cell organization and biogenesis;metabolic process;regulation of biological process;response to stimulus	cytosol;nucleus	protein binding
5713	metabolic process;regulation of biological process;response to stimulus;transport	cytosol;extracellular;membrane;nucleus;organelle lumen;proteasome	catalytic activity;protein binding
8664	cell organization and biogenesis;metabolic process;regulation of biological process	cytoplasm;cytosol;membrane	protein binding;RNA binding
3135	defense response;regulation of biological process;response to stimulus	extracellular;membrane	protein binding
2098	metabolic process	cytoplasm;organelle lumen	catalytic activity;protein binding
5795	metabolic process;regulation of biological process;response to stimulus;transport	cell surface;membrane	catalytic activity;protein binding
29085	metabolic process;regulation of biological process	cytoplasm;cytosol	catalytic activity;protein binding

24139	regulation of biological process	cytoplasm;cytoskeleton	protein binding
2037	cell division;cell organization and biogenesis;transport	cytoplasm;cytoskeleton;cytosol;membrane;nucleus	protein binding;structural molecule activity
10920	cell organization and biogenesis;metabolic process;regulation of biological process;response to stimulus	cytoplasm;cytosol;nucleus	protein binding
9130		nucleus	RNA binding
4688	cellular homeostasis;defense response;metabolic process;regulation of biological process;response to stimulus;transport	cytoplasm;cytosol;vacuole	catalytic activity;enzyme regulator activity;protein binding
6176	cell organization and biogenesis;metabolic process;regulation of biological process;transport	cytosol;ribosome	enzyme regulator activity;protein binding;structural molecule activity
23102	regulation of biological process;transport		enzyme regulator activity;protein binding
374659			catalytic activity;metal ion binding;protein binding
9181	cell division;cell organization and biogenesis;defense response;regulation of biological process;response to stimulus;transport	cytoplasm;cytoskeleton;cytosol;Golgi;membrane	metal ion binding;protein binding
1471	cell death;cell organization and biogenesis;defense response;metabolic process;regulation of biological process;response to stimulus	cytoplasm;endoplasmic reticulum;endosome;extracellular;membrane;organelle lumen;vacuole	enzyme regulator activity;protein binding
92140	cell organization and biogenesis;regulation of biological process;response to stimulus	cytoplasm;endoplasmic reticulum;membrane;nucleus	protein binding;RNA binding
23475	cell organization and biogenesis;metabolic process	cytoplasm;cytosol	catalytic activity;protein binding
4174	cell division;metabolic process	membrane;nucleus	catalytic activity;DNA binding;nucleotide binding;protein binding
1635	metabolic process	cytoplasm;cytosol	catalytic activity;metal ion binding;protein binding
1652	metabolic process;regulation of biological process	cytoplasm	catalytic activity;protein binding
5707	metabolic process;regulation of biological process;response to stimulus;transport	cytosol;extracellular;membrane;nucleus;organelle lumen;proteasome	catalytic activity;enzyme regulator activity;protein binding
54951	metabolic process;regulation of biological process	cytoplasm;cytosol;nucleus	protein binding
3059	cell differentiation;cell organization and biogenesis;regulation of biological process;response to stimulus	cytoplasm;cytosol;membrane;mitochondrion;nucleus	protein binding
4716	cell organization and biogenesis;metabolic process;transport	membrane;mitochondrion	catalytic activity;protein binding
4067	cell communication;cell differentiation;cell organization and biogenesis;cellular component movement;coagulation;defense response;	cytoplasm;cytosol;Golgi;membrane;nucleus;vacuole	catalytic activity;nucleotide binding;protein binding;signal transducer activity
29925	metabolic process	cytoplasm;mitochondrion	catalytic activity;nucleotide binding;protein binding
3927	regulation of biological process;transport	cytoplasm;cytoskeleton	metal ion binding;protein binding;transporter activity
441549	cell differentiation;cell organization and biogenesis;regulation of biological process	endoplasmic reticulum;extracellular	protein binding
23673	cell organization and biogenesis;regulation of biological process;transport	endosome;Golgi;membrane	protein binding
4436	cell differentiation;cell organization and biogenesis;metabolic process;regulation of biological process;response to stimulus	membrane;nucleus	catalytic activity;DNA binding;metal ion binding;nucleotide binding;protein binding
			metal ion binding;protein binding
80273	metabolic process;regulation of biological process;transport	mitochondrion;nucleus;organelle lumen	enzyme regulator activity;nucleotide binding;protein binding
64422	cell communication;cell organization and biogenesis;metabolic process;regulation of biological process;response to stimulus;transpor	cytoplasm;cytosol	catalytic activity;protein binding
51347	metabolic process;regulation of biological process;response to stimulus	cytoplasm;membrane	catalytic activity;enzyme regulator activity;nucleotide binding;protein binding;signal transducer acti
9775	metabolic process;regulation of biological process;response to stimulus;transport	cytoplasm;cytosol;membrane;nucleus;spliceosomal complex	catalytic activity;nucleotide binding;protein binding;RNA binding
8615	cell organization and biogenesis;metabolic process;transport	cytoplasm;cytosol;endoplasmic reticulum;Golgi;membrane	protein binding;RNA binding;transporter activity
8682	cell death;metabolic process;regulation of biological process;response to stimulus;transport	cytoplasm;cytosol;nucleus	protein binding
4599	cell death;cell organization and biogenesis;defense response;regulation of biological process;response to stimulus	cytoplasm;cytosol;endoplasmic reticulum;membrane;nucleus	catalytic activity;nucleotide binding;protein binding
128	metabolic process;response to stimulus	cytoplasm;cytosol;mitochondrion	catalytic activity;metal ion binding;protein binding
10594	cell organization and biogenesis;metabolic process;response to stimulus	membrane;nucleus;spliceosomal complex	catalytic activity;protein binding;RNA binding
10528	metabolic process	cytoplasm;membrane;nucleus	protein binding;RNA binding
6693	cellular component movement;defense response;regulation of biological process;response to stimulus	cell surface;membrane	protein binding;receptor activity;signal transducer activity
3161	metabolic process;regulation of biological process	cell surface;cytoplasm;cytosol;membrane	protein binding
10484	cell organization and biogenesis;transport	cytoplasm;cytosol;endoplasmic reticulum;membrane	metal ion binding;protein binding
64328	regulation of biological process;transport	cytoplasm;cytosol;nucleus	protein binding;transporter activity
23196		cytoplasm;cytosol;membrane;nucleus	RNA binding
81631	cell communication;cell organization and biogenesis;metabolic process;response to stimulus	cytoplasm;cytoskeleton;cytosol;membrane;mitochondrion;vacuole	protein binding
642	metabolic process;response to stimulus	cytoplasm;cytosol;nucleus	catalytic activity;protein binding
83699	cellular homeostasis;regulation of biological process	cytoplasm;membrane;nucleus	catalytic activity;protein binding
51260		cytoplasm	
4179	defense response;metabolic process;regulation of biological process;response to stimulus	cell surface;cytoplasm;Golgi;membrane	catalytic activity;enzyme regulator activity;protein binding;receptor activity
9733	cell organization and biogenesis;cell proliferation;metabolic process;regulation of biological process	cytoplasm;nucleus	protein binding;RNA binding
152137		cytoplasm;cytosol	protein binding
5706	cell organization and biogenesis;metabolic process;regulation of biological process;response to stimulus;transport	cytoplasm;cytosol;membrane;nucleus;proteasome	catalytic activity;nucleotide binding;protein binding;RNA binding
103	cell differentiation;cell organization and biogenesis;defense response;metabolic process;regulation of biological process;response to	cytoplasm;membrane;nucleus;spliceosomal complex	catalytic activity;DNA binding;metal ion binding;protein binding;RNA binding
23517	metabolic process	nucleus;spliceosomal complex	catalytic activity;DNA binding;nucleotide binding;protein binding;RNA binding
51747	cell organization and biogenesis;metabolic process	nucleus;spliceosomal complex	DNA binding;protein binding;RNA binding
55844	cell division;metabolic process;regulation of biological process	cytoplasm;cytosol	catalytic activity;enzyme regulator activity;protein binding
22978	metabolic process	cytoplasm;cytosol	catalytic activity;metal ion binding;nucleotide binding;protein binding
79577	cell organization and biogenesis;metabolic process;regulation of biological process;response to stimulus	cytosol;nucleus	protein binding
9584	metabolic process;regulation of biological process	cytoskeleton;nucleus	protein binding;RNA binding
962	cellular component movement;defense response;regulation of biological process;response to stimulus	membrane	protein binding;receptor activity
3092	cell death;cell differentiation;cell organization and biogenesis;cellular component movement;metabolic process;regulation of biologica	cytoplasm;cytoskeleton;cytosol;Golgi;membrane;nucleus	catalytic activity;motor activity;protein binding;structural molecule activity
10434	metabolic process;regulation of biological process	cytoplasm;cytosol;mitochondrion	catalytic activity;protein binding
4660	regulation of biological process;response to stimulus	cytoplasm;cytosol	enzyme regulator activity;protein binding
6748		endoplasmic reticulum;membrane	
51071	metabolic process;transport	cytoplasm;cytosol;extracellular;nucleus;organelle lumen	catalytic activity
81611	cell organization and biogenesis;regulation of biological process;transport	cytoplasm;nucleus	enzyme regulator activity;protein binding
26999	cell death;cell growth;cell organization and biogenesis;metabolic process;regulation of biological process;response to stimulus	cytoplasm;cytosol;membrane;nucleus	protein binding
6708	cell organization and biogenesis;cellular component movement;metabolic process;regulation of biological process;response to stimul	cytoplasm;cytoskeleton;cytosol;membrane	metal ion binding;protein binding;structural molecule activity
5718	metabolic process;regulation of biological process;response to stimulus;transport	cytoplasm;cytosol;extracellular;membrane;organelle lumen;proteasome	catalytic activity;protein binding
2219	defense response;metabolic process;regulation of biological process;response to stimulus;transport	extracellular;membrane;organelle lumen	catalytic activity;metal ion binding;protein binding;receptor activity;signal transducer activity
2182	cell differentiation;metabolic process;regulation of biological process;response to stimulus;transport	cytoplasm;endoplasmic reticulum;membrane;mitochondrion	catalytic activity;nucleotide binding
7791	cell communication;cell organization and biogenesis;regulation of biological process;response to stimulus	cytoplasm;cytoskeleton;cytosol;membrane;nucleus	metal ion binding;protein binding;RNA binding
6717	metabolic process;regulation of biological process;response to stimulus;transport	cytoplasm;cytosol;endoplasmic reticulum;membrane;mitochondrion	catalytic activity;metal ion binding;protein binding
411	cell organization and biogenesis;cellular component movement;metabolic process;regulation of biological process;response to stimul	cell surface;endoplasmic reticulum;extracellular;Golgi;mitochondrion;organelle lumen;vacuole	catalytic activity;metal ion binding
55727	development	nucleus	protein binding
55737	cell organization and biogenesis;regulation of biological process;response to stimulus;transport	cytoplasm;cytosol;endosome;membrane;mitochondrion;vacuole	protein binding;transporter activity
84154	cell organization and biogenesis;metabolic process;regulation of biological process	nucleus	RNA binding
9146	cell communication;cell organization and biogenesis;metabolic process;regulation of biological process;response to stimulus;transpor	cytoplasm;cytosol;endosome;membrane;vacuole	catalytic activity;metal ion binding;protein binding
84898		membrane	protein binding
55665		cytoplasm;cytosol;nucleus	nucleotide binding
292	regulation of biological process;transport	membrane;mitochondrion;nucleus	protein binding;RNA binding;transporter activity
5130	metabolic process	cytoplasm;cytosol;endoplasmic reticulum;membrane	catalytic activity;protein binding
5604	cell differentiation;cell proliferation;cellular component movement;metabolic process;regulation of biological process;response to stim	cytoplasm;cytoskeleton;cytosol;endoplasmic reticulum;endosome;Golgi;membrane;mitochondrion;nucleus	catalytic activity;enzyme regulator activity;nucleotide binding;protein binding;signal transducer acti
55; 100526664; 99	defense response;regulation of biological process;response to stimulus;transport	membrane	protein binding;receptor activity;signal transducer activity
5198	metabolic process;response to stimulus	cytoplasm;cytosol	catalytic activity;metal ion binding;nucleotide binding
55651	cell organization and biogenesis;metabolic process	nucleus	protein binding;RNA binding
55577	metabolic process	cytosol	catalytic activity;nucleotide binding;protein binding
10653	cell organization and biogenesis;cellular component movement;regulation of biological process;response to stimulus	cytoplasm;extracellular;membrane	enzyme regulator activity
4542			catalytic activity;motor activity;nucleotide binding;protein binding
8666	metabolic process	cytoplasm;cytosol;nucleus	protein binding;RNA binding
164	cell organization and biogenesis;regulation of biological process;transport	cytoplasm;cytosol;endosome;Golgi;membrane	protein binding;transporter activity
4116	metabolic process;regulation of biological process;transport	cytoplasm;nucleus;spliceosomal complex	protein binding;RNA binding
2814	cell organization and biogenesis;coagulation;metabolic process;response to stimulus	membrane	protein binding
9368	cell differentiation;cell organization and biogenesis;cellular homeostasis;regulation of biological process;response to stimulus;transpo	cytoplasm;cytoskeleton;membrane;nucleus	protein binding;structural molecule activity
57103	cell death;metabolic process;regulation of biological process;response to stimulus	cytoplasm;cytosol;membrane;mitochondrion;nucleus	catalytic activity;protein binding
3416	cell organization and biogenesis;cellular homeostasis;metabolic process;regulation of biological process;response to stimulus	cell surface;cytoplasm;cytosol;extracellular;membrane;mitochondrion;nucleus;organelle lumen;proteasome	catalytic activity;metal ion binding;nucleotide binding;protein binding;receptor activity
23451	cell organization and biogenesis;metabolic process;regulation of biological process;response to stimulus	nucleus;spliceosomal complex	DNA binding;protein binding;receptor activity;RNA binding;signal transducer activity
961	cell organization and biogenesis;cellular component movement;regulation of biological process;response to stimulus;transport	membrane	protein binding;receptor activity;signal transducer activity
8301	cell organization and biogenesis;cell proliferation;metabolic process;regulation of biological process;response to stimulus;transport	cell surface;cytosol;endosome;Golgi;membrane;nucleus	protein binding
9647	cell death;cell organization and biogenesis;metabolic process;regulation of biological process;response to stimulus	cytosol	catalytic activity;metal ion binding;protein binding
3032	metabolic process	endoplasmic reticulum;membrane;mitochondrion	catalytic activity;protein binding;RNA binding
6921	metabolic process;regulation of biological process;response to stimulus	cytosol;nucleus	catalytic activity;protein binding;RNA binding
6193	cell organization and biogenesis;metabolic process;regulation of biological process;transport	cytosol;membrane;ribosome	protein binding;RNA binding;structural molecule activity
573	cell death;metabolic process;regulation of biological process;response to stimulus	cytoplasm;cytosol;nucleus	enzyme regulator activity;nucleotide binding;protein binding;signal transducer activity
11165	cell communication;metabolic process	cytoplasm;cytosol;nucleus	catalytic activity;metal ion binding

6637	cell organization and biogenesis;metabolic process;transport	cytoplasm;cytosol;nucleus;spliceosomal complex	protein binding;RNA binding;structural molecule activity
83641			
5552	cell death;cell organization and biogenesis;metabolic process;regulation of biological process;response to stimulus;transport	extracellular;Golgi;membrane;organelle lumen;vacuole	protein binding
125061	metabolic process	cytoplasm;cytosol;nucleus	catalytic activity
9525	cell communication;cell division;cell organization and biogenesis;metabolic process;regulation of biological process;response to stimu	cytoplasm;cytosol;endosome;membrane;nucleus	catalytic activity;nucleotide binding;protein binding;RNA binding
5019	metabolic process;regulation of biological process;response to stimulus	mitochondrion;organelle lumen	catalytic activity;protein binding
55573	cell proliferation	cytoplasm	
5331	cell communication;cell organization and biogenesis;metabolic process;regulation of biological process;response to stimulus	cytoskeleton;cytosol;membrane	catalytic activity;metal ion binding;motor activity;protein binding;signal transducer activity
509	cell organization and biogenesis;metabolic process;transport	membrane;mitochondrion;organelle lumen	catalytic activity;RNA binding;transporter activity
91526			protein binding
79031	cell death;metabolic process;regulation of biological process	cytoplasm;cytosol;nucleus	catalytic activity;protein binding
4204	cell communication;cell differentiation;cell organization and biogenesis;defense response;metabolic process;regulation of biological p	cytoplasm;cytosol;mitochondrion;nucleus	DNA binding;nucleotide binding;protein binding;RNA binding
23163	metabolic process;regulation of biological process;transport	endosome;Golgi;membrane	protein binding
5434	metabolic process;regulation of biological process;response to stimulus	cytosol;nucleus	catalytic activity;DNA binding;protein binding
9129	cell organization and biogenesis;metabolic process	cytosol;nucleus;spliceosomal complex	protein binding;RNA binding
150684	metabolic process;regulation of biological process;transport	cytoplasm;cytosol;endosome;membrane;nucleus	metal ion binding;protein binding
1785	cell organization and biogenesis;metabolic process;regulation of biological process;response to stimulus;transport	cytoplasm;cytoskeleton;cytosol;endosome;Golgi;membrane;nucleus	catalytic activity;nucleotide binding;protein binding
54927	cell organization and biogenesis;metabolic process;regulation of biological process	cytoplasm;membrane;mitochondrion;nucleus	protein binding;structural molecule activity
2801	cell division;cell organization and biogenesis;metabolic process;regulation of biological process;transport	cytoplasm;cytoskeleton;Golgi;membrane;nucleus	catalytic activity;motor activity;protein binding
26058	cell differentiation;metabolic process;regulation of biological process;response to stimulus	cytoplasm;cytosol;endoplasmic reticulum;endosome;Golgi;membrane	protein binding;RNA binding
